# Supplementary material for: Supply-side and demand-side factors influencing uptake of malaria testing services in the community: lessons for scale-up from a post-hoc analysis of a cluster randomised, community-based trial in western Kenya
Source: BMJ Open. 2023 Jun 26;13(6):e070482. doi: 10.1136/bmjopen-2022-070482 (PMC10410802; doi:10.1136/bmjopen-2022-070482)
Supplement: Supplementary data [file bmjopen-2022-070482supp002.pdf]

*Coupon R01 Aim 2 Household Survey Tool* *v. June 8, 2016*

A. *Household survey instrument* (Note: the survey will be administered electronically)

1. Date of interview (DD/MM/YY):

   ☐☐ / ☐☐ / 20 ☐☐

2. Name of interviewer: ______________

3. Interviewer ID: ☐☐

**CU – dropdown menu**

**Demographics**

4. Location: ______________

5. Sub-location: ______________

6. Village name: ______________

**Eligibility criteria**

7. Did any member of the household have fever or malaria-like illness in the last four weeks? *If no, stop.*
   1. Yes
   2. No
   3. Don't Know

8. How long ago did the most recent fever start?

   ☐☐ Days

INSTRUCTION: if the ill individual is 18 or older and not present, stop and save the interview now; recording the time and date saved on your notebook; return to the saved interview if the ill adult is present on a return visit, use the recorded time and date to locate the form.

9. Are you still ill today?
   1. Yes
   2. No

**Patient information**

10. Gender of patient:



Kirui J, *et al. BMJ Open* 2023; 13:e070482. doi: 10.1136/bmjopen-2022-070482

1. Male
2. Female

11. Relationship of patient to household head
    1. Head
    2. Spouse of head
    3. Child
    4. Parent
    5. Grandparent
    6. Grandchild
    7. Aunt /Uncle
    8. Brother / Sister
    9. Cousin
    10. Mother
    11. Father
    12. Employee
    13. OTHER____________________

12. Age of patient: [ | ] Years (if less than 18, skip to respondent information, question 15)

13. Highest school level:
    1. None
    2. Some Primary (not completed Class 8)
    3. Primary completed
    4. Primary completed and some secondary (secondary not completed)
    5. Secondary completed
    6. College
    7. University
14. Occupation
    1. Farming/Livestock keeping
    2. Home maker
    3. Student
    4. Paid employee
    5. Casual employee
    6. Self-employed (not agriculture, livestock) without employees
    7. Self-employed (not agriculture, livestock) with employees
    8. Unpaid family helper in a business
    9. Not working
    10. Unable to work/disabled
    11. Other (Specify)

**Respondent (Answer if age of patient is <18 years. If patient is >18 years, skip to question 19.)**

15. Age of respondent

☐☐ Years

16. Highest school level:
    1. None
    2. Some Primary (not completed Class 8)
    3. Primary completed
    4. Primary completed and some secondary (secondary not completed)
    5. Secondary completed
    6. College
    7. University

17. Gender of respondent:
    1. Male
    2. Female

18. Relationship of respondent to child:
    1. Parent
    2. Aunt/Uncle
    3. Grandparent
    4. Sibling
    5. Other

**Endpoints** *Note: The wording of the questions in this section are directed at a respondent 18 or over; when administered as an electronic survey, there will be two parallel sets of questions that branch based on whether a child or adult was the patient.*

19. How severe was the illness?
    1. Very severe
    2. Severe
    3. Moderately severe
    4. Mild
    5. Very mild

20. Did you take any action for your illness? *Note: taking drugs at home is an action. If "Don't remember" or "No", skip to COVARIATES section, question 56.)*
    1. Yes
    2. No
    3. Don't remember

21. What did you do FIRST for your illness? *Only one answer*
    1. Contact CHW

2. Go to a government health centre
3. Go to a government dispensary
4. Go to a government hospital
5. Go to a private clinic or mission facility
6. Go to a shop or pharmacy
7. Take some drugs at home
8. visit traditional healers
9. visit religious/cultural healers
10. OTHER: ____________

22. What were the MAIN reasons for deciding to do that? *Multiple answers OK*
    1. I already had the drugs at home
    2. It is the nearest place (facility or drug shop)
    3. The services are free
    4. Money- this was most affordable
    5. Wanted to see a Doctor
    6. Illness very serious
    7. Illness not very serious
    8. Time was limited / this was the fastest
    9. Good quality service
    10. There are no drugs in the facility
    11. There is laboratory testing
    12. OTHER: ____________

23. How long after the illness began did you do that?
    1. same day
    2. next day
    3. after two days
    4. more than two days later
    5. OTHER: ____________

24. Did you take any other action? *If no, skip to question 26.*
    1. Yes
    2. No
    3. Don't remember

25. If yes, what did you do? A*llow multiple answers*

    1. Contact CHW
    2. Go to a government health centre
    3. Go to a government dispensary
    4. Go to a government hospital
    5. Go to a private clinic or mission facility
    6. Go to a shop or pharmacy

7. Take some drugs at home
8. visit traditional healers
9. visit religious/cultural healers
10. OTHER: ____________

26. How long did you take to fully recover?

[ | ] Days (99=not yet recovered)

27. Did you have any laboratory test done during your illness? *PROBE. Ask about blood, stool or urine taken to help find out about the illness*

1. Yes
2. No
3. Don't remember

28. Did you have a malaria test? *If no or don't remember, skip to question 42.*

1. Yes
2. No
3. Don't remember
4. Don't know

29. How long after the start of the illness did you have a malaria test (the first test, if more than one)?

1. same day
2. next day
3. after two days
4. OTHER: ____________

30. Was it a microscopy test or a rapid diagnostic test? (If both, record in 'OTHER')

1. RDT
2. microscopy
3. Don't remember or don't know
4. OTHER (specify): ______________

31. Where was the malaria test done?

1. CHW (go to Question 32, else skip to 35.)
2. shop or pharmacy
3. private clinic/hospital
4. government dispensary
5. government hospital
6. government health center
7. private lab
8. OTHER: ____________

32. If a CHW did the test, where was it done?
    1. In my village
    2. In another village but the same CU (in which the survey is being conducted)
    3. Another CU (write name: ____________ )
    4. Don't know/don't remember

33. Did the CHW give you a voucher to purchase a drug?
    1. Yes (Ask question 34 else skip to 35.)
    2. No
    3. Don't know/don't remember

34. Did you use the voucher to purchase a drug?
    1. Yes
    2. No
    3. Don't know/ don't remember

35. How much did the malaria test cost (Ksh.)? If more than one test, list both
    _______________**Free text**

36. What were the results of the malaria test? (If more than one test, use OTHER)
    1. Positive
    2. Negative
    3. Don't remember / Don't know
    4. OTHER__________________

37. How likely is it that the result of your malaria test was correct?
    1. Very likely
    2. Likely
    3. 50-50
    4. Unlikely
    5. Very unlikely
    6. Don't know
    7. OTHER (use if have different beliefs about multiple tests)

38. Record observed for test? *Interviewer question (this can include study forms)*
    1. Yes
    2. No (Skip to question 42.)

*If yes (record observed), interviewer should answer the next 3 questions:*

39. Where does the record report the test was done? (multiple answers possible)

1. CHW
2. shop or pharmacy
3. private clinic/hospital
4. government dispensary
5. government hospital
6. government health center
7. private lab
8. Not specified / Cannot be determined
9. OTHER: _____________

40. What type of malaria test does the record report was done? (if more than one test, use OTHER)

1. RDT
2. microscopy
3. Not specified / Cannot be determined
4. OTHER (specify): _______________

41. What were the results of the malaria test according to the record? (if more than one test, use OTHER)

1. Positive
2. Negative
3. Not specified / Cannot be determined
4. OTHER _____________________

42. How likely is it that the illness was malaria?

1. Very likely
2. Likely
3. 50-50
4. Unlikely
5. Very unlikely
6. Don't know
7. No response

43. Were any drugs taken for the illness? *If no or don't remember, skip to question 55.*

1. Yes
2. No
3. Don't remember

44. Which medicines did you take? (Multiple answers possible) SKIP PATTERN: If 1 or 4 (ACT), continue with next question. Else, skip to 53

1. Coartem/Artefan/other AL
2. Quinine

3. SP/Fansidar/Metakelfin/Moladar
4. Other ACT (e.g Duocotexin)
5. Amoxyl
6. Septrin
7. Ciprofloxaccin/Norfloxaccin
8. Panadol/Brufen/Hedex/Painkiller/Action/Maramoja
9. Don't know/Don't Remember
10. Other (specify: __________ )

45. Packaging observed for ACT? *Interviewer question. If YES, ask question 46. If NO, skip to question 47.*

1. Yes
2. No

46. If ACT package observed, what brand? *Interviewer question*.

1. Coartem (AL)
2. Aretefan (AL)
3. Duocotexin
4. P-Alaxin
5. AL (IPCA brand)
6. AQ AS
7. Lonart (AL)
8. Artequick
9. Lumartem (AL)
10. Combiart (AL)
11. L-artem (AL)
12. OTHER: __________

47. How long after the start of the illness did you start taking the ACT?

1. same day
2. next day
3. after two days
4. OTHER: __________

48. Did you start taking the ACT before or after the malaria test? *Only ask this if YES to 28.*

1. Before
2. After
3. Don't remember
4. Not Applicable

If respondent answered "1-Coartem/Artefan/other AL" to question 44, ask questions 49-52, else skip to question 53.

49. If AL, how many pills were given? *Only ask if 44 = 1 (AL ONLY)*

1. 6
2. 12
3. 18
4. 24
5. Don't know/remember
6. OTHER: ___________

50. If AL, did you / patient take all of the tablets? *Only ask if 44 = 1 (AL ONLY)*

1. Yes
2. No
3. Not sure
4. Don't remember
5. Not yet—still taking them

51. Where was the AL obtained? MULTIPLE RESPONSES POSSIBLE
*(If the drugs were already in the home at the onset of illness, record this but find out from where they were originally obtained) Only ask if 44 = 1, 4 ie any ACT.*

1. Used drugs at home
2. Pharmacy, chemist or duka la dawa with a prescription
3. Pharmacy, chemist or duka la dawa; no prescription
4. CHW
5. Neighbor
6. Private health facility
7. Government health facility
8. general store
9. OTHER: _______________
10. Don't remember/don't know

52. Did you get AL at the first place you sought treatment? *Only ask if 44 = 1,4 (ACT ONLY)*

1. Yes
2. No
3. Yes and another place
4. Don't know/don't remember

53. How likely do you think the malaria drugs you took were effective? (ask if respondent answered 1, 2, 3 or 4 to question 44.)

1. Very likely
2. Likely
3. 50-50
4. Unlikely

5. Very unlikely
6. Don't know
7. No response

54. How much did you spend for all the drugs you purchased for this illness? (-1 should be used for 'I don't know/don't remember')
    1. ___________________________

55. How much did you spend in total to take care of this illness, including testing, drugs, transportation, admission fees, registration and consultation fees? (-1 should be used for 'I don't know/don't remember')
    1. ___________________________

**Covariates** *THESE QUESTIONS DIRECTED AT RESPONDENT, EVEN IF NOT THE PATIENT*

56. GPS coordinates: _____ (PLACED AT THE END OF THE ELECTRONIC SURVEY)

57. Does your household have any mosquito nets? (*If no or don't know, skip to question 60.*)
    1. Yes
    2. No
    3. DK

58. Did THE PATIENT sleep under the net last night?
    1. Yes
    2. Has a net for sleeping space, but did not use it last night.
    3. Does not have net for sleeping space
    4. Do not Remember/Don't Know

59. Was the net pre-treated with insecticide when you got it?
    1. Yes
    2. No
    3. Not Applicable (no net for sleeping space)
    4. Do not Remember/Don't Know

60. Is there a CHW in your village that provides any services or health education? *If no or do not know skip to question 66.*
    1. Yes
    2. No
    3. Don't Know

61. Which services does the CHW provide? *Multiple responses possible*
    1. Information

2. Referral
3. Polio immunization
4. Advice
5. Testing for HIV
6. Testing for malaria
7. Testing for diabetes
8. Testing for hypertension
9. Testing for pregnancy
10. Coupon for antimalarial drug
11. Bednets
12. Water treatment (ie Lifestraw)
13. Other (specify) ______________

62. Does your CHW provide malaria diagnostic testing?
    1. Yes
    2. No
    3. Don't know/Don't remember

63. Does your CHW provide a discount on a malaria drug in case the test is positive for malaria?
    1. Yes
    2. No
    3. Don't know/Don't remember

64. Have you ever contacted a CHW near you in case of illness for yourself or someone in your household?
    1. Yes
    2. No (*skip to question 66*)
    3. DR/DK (*skip to question 66*)

65. What did the CHW do? (multiple responses possible)
    1. Refer
    2. Give medicine
    3. Accompany to shop
    4. Give advice
    5. Perform a malaria test
    6. Give a coupon for an antimalarial drug
    7. Other (specify)______________

66. Do you know what a rapid diagnostic test for malaria is? *Show example of cassette*
    1. Yes
    2. No → skip to 71

67. Have you ever had an RDT test for malaria?
    1. Yes
    2. No
    3. Don't know/don't remember

68. Do you know anyone besides one of your household members who has had an RDT?
    1. Yes
    2. No

3. Don't know/don't remember

69. If you have fever and your malaria RDT is negative, how likely is it that the test is correct?

1. Very likely
2. Likely
3. 50-50
4. Unlikely
5. Very unlikely
6. Don't know
7. No response

70. If you have fever and your malaria RDT is positive, how likely is it that the test is correct?

1. Very likely
2. Likely
3. 50-50
4. Unlikely
5. Very unlikely
6. Don't know
7. No response

71. Is malaria common in your village

1. Yes
2. No
3. Don't know/not sure

72. If ten people (watu) from your village have fever, how many of them are likely to have malaria? ______________(number 0-10 or 99 for don't know)

73. If you have malaria, and you take AL, how likely is it that you will be completely better in 3 days?

1. Very likely
2. Likely
3. 50-50
4. Unlikely
5. Very unlikely
6. Don't know
7. No response

**SES Questions**
(Source:pilot)

74. Does your household have the following items

1. Electricity
2. Television
3. Refrigerator
4. Radio

5. Mobile phone
6. Motorcycle
7. car/truck
8. Bank account
9. None

75. How many of the following livestock does your household have
    1. Cows
    2. Sheep
    3. Goats
    4. Pigs
    5. Donkeys

76. What kind of toilet does your household have
    1. Flush or pour flush toilet
    2. VIP/Ventilated improved pit
    3. Pit latrine WITH slab
    4. Pit latrine without slab
    5. Composting toilet
    6. Bucket toilet
    7. No facility/bush/field
    8. Other

77. Main source of drinking water for your household
    1. Piped water/public tap/borehole
    2. Unprotected well
    3. Protected well
    4. Protected spring
    5. Unprotected spring
    6. Rain Water
    7. River Water
    8. Other:______________

78. Main material of the floor in your house
    1. Earthen
    2. Cement
    3. Floor tiles
    4. Wood planks
    5. Polished wood
    6. Other

79. Main material of the walls in your house
    1. Stone
    2. Brick
    3. Timber
    4. Iron Sheet
    5. Mud

6. Wood
7. Cement
8. Other

80. Main material of the roof of your house
    1. Iron sheets
    2. Roof tiles
    3. Grass thatched
    4. Wood

81. How many acres/hectares/feet of land for farming does your household own
    1. None
    2. Acres: ___________
    3. Hectares: ________
    4. Square feet(xx by xx):_________
