# Supplementary material for: Supply-side and demand-side factors influencing uptake of malaria testing services in the community: lessons for scale-up from a post-hoc analysis of a cluster randomised, community-based trial in western Kenya
Source: BMJ Open. 2023 Jun 26;13(6):e070482. doi: 10.1136/bmjopen-2022-070482 (PMC10410802; doi:10.1136/bmjopen-2022-070482)
Supplement: Supplementary data [file bmjopen-2022-070482supp001.pdf]

Coupon R01 CHV Participation Assessment

April 13, 2016

Note on administration: This survey is intended to be administered orally by an enumerator to a CHV.

I. Background Information

1) Sub-county:
    1. Bungoma East
    2. Kiminini

2) Name of CU that the CHV serves: ________________________

3) CHV's gender:
    1. Male
    2. Female

4) Age: ________ Years

5) Highest school level:
    1. None
    2. Some Primary (not completed Class 8)
    3. Primary completed
    4. Primary completed and some secondary (secondary not completed)
    5. Secondary completed
    6. College
    7. University

6) For how long have you served as a CHV?
    1. < 1
    2. > 1-3 Years
    3. > 3-5 Years
    4. > 5 Years

7) Apart from serving as a CHV, what other occupation(s) or role(s) do you have?

1. *Write-in response:* ________________________
2. None

8) How many trainings for CHV activities of any kind have you had since the RDT training for this malaria project?
    1. None
    2. One
    3. Two
    4. Three
    5. Four
    6. Five
    7. More than five

9) How long ago was your most recent training for CHV activities of any kind?
    1. Within the past week
    2. Between one week and one month ago
    3. Between one and three months ago
    4. Between three months and 1 year ago
    5. More than one year ago

10) What was the topic of your most recent training?
    1. Malaria (this project)
    2. Malaria (another project)
    3. Maternal health
    4. Polio campaign
    5. Other vaccination program
    6. Other child health topic not mentioned above

    7. Other: _______

II. Self-description of Role

11) In the past month, what activities have you carried out as a CHV overall?
    (Enumerator: check all that are mentioned; do not read the list)
    1. Malaria testing
    2. Provide vouchers for malaria drugs
    3. Mosquito net distribution
    4. Provide other malaria-related services/counseling

5. Polio campaign participation
6. Other vaccination campaign(s)
7. Child health services not mentioned above
8. Maternal health services not mentioned above
9. Giving health education to households
10. None
11. Other: ______________________________
____
____

12) How many times have you met with your CHEW in the past 3 months?
1. Once
2. Twice
3. Three times
4. More than three times
5. None

13) What activities do you perform specifically for the malaria project?
(Enumerator: check all that are mentioned; do not read the list)
1. Perform malaria RDTs
2. Provide vouchers for malaria drugs
3. Provide malaria drugs
4. Provide referrals
5. Provide counseling / advice
6. Other: ______________________________

14) For how many clients have you performed an RDT in the past month?
1. 1 client
2. 2-5 clients
3. 5 clients and above

15) What are the requirements for a client to receive an RDT under this malaria project?
(Enumerator: check all that are mentioned; do not read the list)
1. No requirements – anyone who asks can be tested
2. Must live in my CU
3. Has a documented fever (i.e., as measured by me)

4. Has a documented fever or reports a history of recent fever
5. Has a documented fever or reports a history of recent fever or reports any symptoms of malaria
6. Consent must be provided by the client (or by the parent/guardian of a minor)
7. Must not be visibly pregnant

8. Other: ________________________________

16) What motivates you to participate in this malaria project?
1. Personal desire
2. Previous experience
3. Family
4. Organization (partner and GOK)
5. Monetary incentive (money given to group after 6 months and transport to attend supervision and supplies replenishment)
6. In-kind incentive (e.g., airtime for your phone)
7. Support from CHEWs
8. Support from link facilities in attending to clients referred for care
9. Other:__________________________

III. Satisfaction with Role

17) What are the bad things about participating in this project?
(Enumerator: check all that are mentioned; do not read the list)

(Open-ended response): ____

____

____

18) What are the good things about participating in this project?
(Enumerator: check all that are mentioned; do not read the list)

(Open-ended response): ____

____

Please indicate your level of agreement with the following statements with regards to the malaria project **only.**

Question	Strongly Agree	Somewhat Agree	50-50	Somewhat Disagree	Strongly Disagree
19) Overall, I am confident in my ability to perform my role in this malaria project.					
20) I receive enough supervision in my work from the malaria project study staff.					
21) I believe the clients that test positive for malaria follow the advice that I give them					
22) I believe the clients that test negative for malaria follow the advice that I give them					
23) The people in my community appreciate my work on the malaria project.					
24) I have gained more recognition in my community since I began testing for malaria.					
25) I am proud of the work that I do in the malaria project.					
26) I have gained valuable knowledge as a result of my role in this malaria project.					
27) Overall, I think the malaria project is an important activity for me to be involved in.					
28) Overall, I enjoy carrying out my role in this malaria project.					
29) I would like to continue with my role in this malaria project.					

30) *If the CHV answers "50-50", somewhat disagree" or "slightly disagree" to continuing their role in the project*, why?
(Enumerator: check all that are mentioned; do not read the list)

1. Requires too much time
2. Requires too much effort
3. Don't receive enough appreciation
4. Don't receive compensation
5. Frustrated that they can't provide drugs directly to clients
6. Conflicts with other CHV activities
7. Conflicts with non-CHV activities / responsibilities
8. Other ____

IV. Logistics & Challenges of Role

A. Implementation

i. Community awareness & trust of services

Question	Always aware	Usually aware	Sometimes aware	Rarely aware	Never aware
31) Of all of the clients that you provide services to for any reason, how often are the clients already aware that you provide RDT testing?					
32) Of the clients that visit you with a fever or other symptoms of malaria, how often are these clients already aware that you provide RDT testing?					
33) Of the clients that visit you with a fever or other symptoms of malaria, how often are these clients already aware that you provide vouchers for malaria drugs?					

34) How do you think your clients learn about the malaria services (testing and vouchers) that you provide?
(Enumerator: check all that are mentioned; do not read the list)

1. From me
2. From family members
3. From neighbors / friends
4. From village leaders
5. From health facility staff
6. From malaria project staff

7. Other: ____________________________

Please indicate your level of agreement with the following statements.

Question	Strongly Agree	Somewhat Agree	50-50	Somewhat Disagree	Strongly Disagree
35) The malaria RDT that I do can be trusted just as much as a malaria RDT done in a health facility.					
36) The clients that test positive for malaria trust the results of the test					
37) The clients that test negative for malaria trust the results of the test					
38) People I test for malaria believe I am actually testing for HIV.					

39) Do any of your clients refuse an RDT when you offer it?
1. Yes
2. No

40) *If Yes to above,* What are the reasons that you think these clients refuse?
(Enumerator: check all that are mentioned; do not read the list)
3. They don't think it's necessary
4. They don't trust me
5. They don't trust the test
6. They are already convinced they have malaria
7. They plan to buy antimalarial drugs regardless of test result
8. They don't want to be pricked
9. They think it may really be an HIV test

10. Other: ______________________

ii. Logistics & Challenges

41) What are all of the ways that clients find you to receive a malaria RDT?
(Enumerator: check all that are mentioned; do not read the list)
1. Clients visit me at my home
2. Clients visit me at a health facility

3. Clients visit me at church
4. I visit clients at their homes
5. Client visit me at another public location
6. Client visit me at another private location
7. Clients call my mobile phone
8. The study staff contacts me
9. The health facility contacts me

10. Other:_____________________

42) Where do you provide testing for malaria? *(Enumerator: check all that are mentioned; do not read the list)*
    1. At my home
    2. At a health facility
    3. At church
    4. At client's home
    5. At another public location
    6. At another private location
    7. At CHV Office
    8. In Church
    9. In Barazas (public meeting)

    10. Other:_____________________

43) How often do you lack all of the supplies you need to perform the malaria services of testing and providing vouchers when necessary?
    1. Always
    2. Sometimes
    3. 50-50
    4. Rarely
    5. Never

44) Which, if any, supplies related to the malaria services you perform for this project have you had a stock-out of in the past month?
    (Enumerator: check all that are mentioned; do not read the list)
    1. RDTs
    2. Buffer
    3. Lancets
    4. Alcohol swabs
    5. Cotton wool
    6. Gloves
    7. Safety disposal box
    8. Yellow waste bags
    9. Plastic (zip-loc) bags
    10. Thermometer
    11. Thermometer batteries

12. Pen
13. Encounter forms
14. Voucher forms

15. Other:____________________________

45) How would prefer your consumption of malaria project supplies be monitored?
   1. CHV counts the supplies physically
   2. CHV references filled encounter forms
   3. CHV uses inventory card to document consumption of supplies
   4. CHV relies on the supervision team to monitor the supplies

46) What are the main challenges and barriers you face in implementing the malaria project? *(Enumerator: check all that are mentioned; do not read the list)*
   1. Community is unaware that I offer malaria RDTs
   2. Community is unaware that I offer vouchers for malaria drugs
   3. The clients don't always trust the test results I give them
   4. The clients are unhappy that I can't provide the malaria drugs to them directly
   5. Transport
   6. Not enough training
   7. Not enough supervision from study staff
   8. Not enough supervision from CHEWs
   9. Lack of inventory systems to monitor supplies
   10. Competing activities within the community
   11. Other roles by other NGOs interfere with Malaria project
   12. My other main occupation does not give me time to participate in Malaria project
   13. Very few clients coming for test
   14. Nature of the community not willing to interact with CHVs
   15. Cultural practices e.g use of herbal medicine, seeking help from traditional healers
   16. Level of education of community member (the less educated don't understand the importance of malaria test and the very educated not willing to be tested by the community health volunteer
   17. Lack of mobilization at the community
   18. Family relationships are affected
   19. Other: ________________________________

47) Does frequent supervision improve your ability to provide quality malaria testing?
   1. Yes
   2. No

48) *If yes above*, how frequently would you prefer supervision to be carried out?
   1. Once a week
   2. Once every two weeks
   3. Once a month
   4. Once every two months

49) What is the main purpose of supervision activities conducted by the malaria project team?
(Enumerator: check only one)
1. To monitor whether I make any mistakes
2. To provide additional training
3. To ensure data quality

50) What would help you perform your role in the malaria project better?
(Enumerator: check all that are mentioned; do not read the list)
1. More training
2. More supervision from study team
3. More supervision from CHEWs
4. More regular restocking of supplies
5. Raising community awareness about the project
6. Compensation for time spent
7. Sharing my CHV duties with another CHV
8. Other: ___________________________________

iii. Time required

51) On average, how long does it take you to complete a visit with a patient complaining of malaria symptoms?

____________________ Minutes

52) In the last week, what portion of your CHV activities have been related to the malaria project?
1. Less than half
2. About half
3. More than half

53) How many hours do you think you spend on the malaria project activities in an average week (including seeing clients, filling forms, meeting with malaria study staff, etc.)?

____________________ Hours

54) How many hours do you think you spend on all of your other CHV activities and responsibilities in an average week (including seeing clients, reporting, restocking, trainings, etc.)?

____________________ Hours

55) Do you think the total time you spend on all CHV activities has decreased, increased, or stayed about the same since you began the malaria project activities?

1. Decreased
2. Stayed about the same
3. Increased

B. <u>Relation to other CHV activities</u>

56) Has participating in the malaria project had an effect on your other CHV activities?
    1. Yes
    2. No

57) If yes, in what ways:
    (Enumerator: check all that are mentioned; do not read the list)
    1. Improved skills applicable to other CHV activities
    2. More clients visit me for non-malaria services
    3. Less time to complete other CHV activities

    4. Other: ____________________

58) Please indicate to what extent you agree with the statement: Due to my involvement with the malaria project, I now provide more non-malaria health services than before the malaria project.
    1. Strongly agree – I provide much more non-malaria health services than before
    2. Somewhat agree
    3. Neither agree nor disagree – it has stayed the same
    4. Somewhat disagree
    5. Strongly disagree – I provide much less non-malaria health services than before

(Only ask the next 2 questions if "strongly agree" or "somewhat agree" above):

59) If "strongly agree" or "somewhat agree" above: What are the other (non-malaria) services you provide more of?
    (Enumerator: check all that are mentioned; do not read the list)
    1. Maternal health services
    2. Vaccinations
    3. Providing health information
    4. *… (Other typical CHV activities to include in this list?)*

    5. Other: ___________________________

60) To whom do you provide more non-malaria services than before the malaria project started? (select all that apply):
    (Enumerator: check all that are mentioned; do not read the list)
    1. Men
    2. Women

3. Children

61) Please indicate to what extent you agree with the statement: There are other CHV activities or services I would normally have performed in the last month that I did not because the malaria project took too much of my time.
    1. Strongly agree
    2. Somewhat agree
    3. 50-50
    4. Somewhat disagree
    5. Strongly disagree

62) If yes, what activities / services?
    (Enumerator: check all that are mentioned; do not read the list)
    1. Maternal health services
    2. Vaccinations
    3. Providing health information
    4. *… (Other typical CHV activities to include in this list?)*

    5. Other: ____________________________

63) Please indicate to what extent you agree with the statement: There are other responsibilities outside of my work as a CHV that I would normally have performed in the last month that I did not because my work as a CHV took too much of my time.
    1. Strongly agree
    2. Somewhat agree
    3. 50-50
    4. Somewhat disagree
    5. Strongly disagree

64) *If "strongly agree" or "somewhat agree" above:* What responsibilities outside of your role as a CHV have been affected? (childcare, other volunteering, church, education/study, family, etc.)
    (Enumerator: check all that are mentioned; do not read the list)
    1. Childcare
    2. Family responsibilities
    3. Other work
    4. Other volunteering / community activities
    5. Religious activities

    6. Other: ____________________________

65) What, if an, relationships have been affected by your work as a CHV on this malaria project? *(Enumerator: check all that are mentioned; do not read the list)*
    1. Children
    2. Spouse
    3. Siblings
    4. Parents
    5. Other family members not mentioned above

6. Neighbors
7. Friends
8. Other: ___________________________

66) What do you think are the most important services you provide as a CHV?
(Enumerator: check all that are mentioned; do not read the list)
1. Malaria testing
2. Provide vouchers for malaria drugs
3. Mosquito net distribution
4. Provide other malaria-related services/counseling
5. Polio campaign participation
6. Other vaccination campaign(s)
7. Child health services not mentioned above
8. Maternal health services not mentioned above
9. Giving health education to households
10. None
11. Other: ___________________________
____
____

67) What do you think is the most important service you provide as a CHV?
(Enumerator: Only allow one answer)
1. Malaria testing
2. Provide vouchers for malaria drugs
3. Mosquito net distribution
4. Provide other malaria-related services/counseling
5. Polio campaign participation
6. Other vaccination campaign(s)
7. Child health services not mentioned above
8. Maternal health services not mentioned above
9. Giving health education to households
10. None
11. Other: ___________________________
____
____

68) In order to monitor the quality of the tests done, some CHVs in this project were randomly chosen to use a device called a DekiReader that automatically reads the RDT cassette and displays the results. Did you use this device?
1. Yes
2. No
3. I was selected but I couldn't use it (too difficult)
4. Not sure

If 1 or 3, ask next 3 questions:

69) To what extent would you agree with the statement, "The DekiReader was easy to use correctly"?

1. Strongly agree
2. Somewhat agree
3. 50-50
4. Somewhat disagree
5. Strongly disagree

70) To what extent would you agree with the statement, “The Deki Reader helped me improve my ability to perform RDTs correctly”?
    1. Strongly agree
    2. Somewhat agree
    3. 50-50
    4. Somewhat disagree
    5. Strongly disagree

71) If you had the option to use the Deki Reader for every test as part of your routine work, would you want to have it or not?
    1. Yes
    2. No
    3. Don’t Know / Unsure

72) Do you have any concerns or suggestions to improve the implementation of the malaria project?
    (Open-ended response): ______________________________

    ____

    ____

    ____

Thank you for your participation and feedback!

Checklist for performing Rapid Diagnostic Testing correctly

Name of CHV ____________________ Community Unit ____________________

Name of observer____________________________ Date _____________________

No.	Task	Task Category	Correctly done No = 0 Yes =1	Observer's comments
**A. RDT preparation**				
1.	Assembles RDT packet, buffer, swab, lancet			
2.	Read expiration date			
3.	Remove contents of test packet			
4.	Write patient's name on cassette			
**B. Patient preparation**				
5.	Identify patient's details and record patient's name and date on the RDT cassette			
6.	Explain procedure to patient. Provide reassurance, as needed			
7.	Wear gloves	Safety		
**C. Blood collection + dispensing**				
8.	Select 4th finger of left hand for blood collection			
9.	Clean finger with alcohol swab and allow it to dry	Safety		
10.	Prick finger firmly with sterile lancet	Safety		
11.	Discard lancet in sharps bin immediately after pricking finger. Do not set it down first	Safety		
12.	DO NOT squeeze finger excessively			
13.	Collect an adequate volume of blood with pipette	Accuracy		
14.	Dispense blood in correct well	Accuracy		
15.	Discards the pipette in the sharps box	Safety		
16.	Dispose of gloves, wrappers, alcohol swab, loop, desiccant and cassette in non-sharps container	Safety		
**D. RDT procedure and reading results**				
17.	Dispense correct volume of buffer	Accuracy		
18.	Wait for twenty (20) minutes	Accuracy		
19.	Verify internal test control			
20.	Read results correctly	Accuracy		
**E. Recording results**				
21.	Records date and time correctly on encounter form	Treatment		
**F. Deliver the results**				
22.	Explain to the patient the results and what they mean	Treatment		
