# Supplementary material for: Supply-side and demand-side factors influencing uptake of malaria testing services in the community: lessons for scale-up from a post-hoc analysis of a cluster randomised, community-based trial in western Kenya
Source: BMJ Open. 2023 Jun 26;13(6):e070482. doi: 10.1136/bmjopen-2022-070482 (PMC10410802; doi:10.1136/bmjopen-2022-070482)
Supplement: Author's manuscript [file bmjopen-2022-070482.draft_revisions.pdf]

BMJ Open

BMJ Open is committed to open peer review. As part of this commitment we make the peer review history of every article we publish publicly available.

When an article is published we post the peer reviewers' comments and the authors' responses online. We also post the versions of the paper that were used during peer review. These are the versions that the peer review comments apply to.

The versions of the paper that follow are the versions that were submitted during the peer review process. They are not the versions of record or the final published versions. They should not be cited or distributed as the published version of this manuscript.

BMJ Open is an open access journal and the full, final, typeset and author-corrected version of record of the manuscript is available on our site with no access controls, subscription charges or pay-per-view fees (http://bmjopen.bmj.com).

If you have any questions on BMJ Open's open peer review process please email info.bmjopen@bmj.com

Supply and demand-side factors influencing uptake of malaria testing services in the community; lessons for scale-up from a cluster-randomized, community-based trial in western Kenya

Journal:	*BMJ Open*
Manuscript ID	bmjopen-2022-070482
Article Type:	Original research
Date Submitted by the Author:	28-Nov-2022
Complete List of Authors:	Kipkoech, Joseph; Academic Model Providing Access to Healthcare<br>Malinga, Josephine; Duke Global Health Institute<br>Sang, Edna; Duke Global Inc, Kenya<br>Ambani, George; Academic Model Providing Access to Healthcare<br>Abel, Lucy; Academic Model Providing Access to Healthcare<br>Nalianya, Erick; Duke Global Inc, Kenya<br>Namae, Jane; Moi University College of Health Sciences<br>Boyce, Matthew; Georgetown University<br>Laktabai, Jeremiah; Academic Model Providing Access to Healthcare; Moi University College of Health Sciences<br>Menya, Diana; Moi University School of Public Health<br>O'Meara, Wendy; Duke Global Health Institute, ; Moi University,
Keywords:	Public health < INFECTIOUS DISEASES, Community child health < PAEDIATRICS, International health services < HEALTH SERVICES ADMINISTRATION & MANAGEMENT

SCHOLARONE™
Manuscripts

I, the Submitting Author has the right to grant and does grant on behalf of all authors of the Work (as defined in the below author licence), an exclusive licence and/or a non-exclusive licence for contributions from authors who are: i) UK Crown employees; ii) where BMJ has agreed a CC-BY licence shall apply, and/or iii) in accordance with the terms applicable for US Federal Government officers or employees acting as part of their official duties; on a worldwide, perpetual, irrevocable, royalty-free basis to BMJ Publishing Group Ltd ("BMJ") its licensees and where the relevant Journal is co-owned by BMJ to the co-owners of the Journal, to publish the Work in this journal and any other BMJ products and to exploit all rights, as set out in our licence.

The Submitting Author accepts and understands that any supply made under these terms is made by BMJ to the Submitting Author unless you are acting as an employee on behalf of your employer or a postgraduate student of an affiliated institution which is paying any applicable article publishing charge ("APC") for Open Access articles. Where the Submitting Author wishes to make the Work available on an Open Access basis (and intends to pay the relevant APC), the terms of reuse of such Open Access shall be governed by a Creative Commons licence – details of these licences and which Creative Commons licence will apply to this Work are set out in our licence referred to above.

Other than as permitted in any relevant BMJ Author's Self Archiving Policies, I confirm this Work has not been accepted for publication elsewhere, is not being considered for publication elsewhere and does not duplicate material already published. I confirm all authors consent to publication of this Work and authorise the granting of this licence.

For peer review only

Supply and demand-side factors influencing uptake of malaria testing services in the community; lessons for scale-up from a cluster-randomized, community-based trial in western Kenya

Authors and affiliations:

Joseph Kipkoech[1*], Josephine Malinga[2], Edna Sang[3], George Ambani[1], Lucy Abel[1], Erick Nalianya[3], Jane Namae[4], Matthew Boyce[5], Jeremiah Laktabai[1,4], Diana Menya[6], Wendy Prudhomme O'Meara[2,6]

[1]Academic Model Providing Access to Healthcare, Eldoret, Kenya

[2]Duke Global Health Institute, Durham, North Carolina, USA.

[3]Duke Global Inc, Kenya.

[4]School of Medicine, Moi University College of Health Sciences, Eldoret Kenya.

[5]Georgetown University, Washington DC, USA.

[6]School of Public Health, Moi University College of Health Sciences, Eldoret, Kenya.

[*]Corresponding author

Keywords: Community-based programs, demand and supply, health interventions, malaria, diagnostic testing, Community Health Workers

List of abbreviations: Artemisinin Combination Therapy (ACT), Community Health Extension Worker(CHEW), Community Health Worker (CHW), Community Unit (CU), Global Positioning System (GPS), Ministry of Health (MOH), Rapid Diagnostic Test (RDT), World Health Organization (WHO)

For peer review only

Corresponding Author: Joseph Kipkoech, Email: josepheddykipkoech@gmail.com

Abstract

Objectives: Maximizing the impact of community-based programs requires understanding how the supply of, and demand for, the intervention interact at the point of delivery. We present results from a large-scale community health worker (CHW) study designed to increase the uptake of malaria diagnostic testing in a rural, malaria-endemic region.

Methods: CHWs provided free malaria Rapid Diagnostic Test(mRDT). Those with a positive test received a discounted first-line antimalarial over-the-counter. We conducted a survey of household members with fever in the last four weeks to collect individual outcomes at 12- and 18-months post-implementation. We collected monthly testing data from 244 participating CHWs and conducted semi-structured interviews with a random sample of 70 CHWs.

Results: From the survey, 55% (n=948/1738) reported having a malaria test for their recent illness with 38% having been tested by a CHW. Being aware of a local CHW (Adj.OR=1.50, 95%CI:1.10-2.04) and belonging to a wealthy household (Adj.OR=1.53, 95%CI:1.14-2.06) were associated with higher testing uptake. Poorer households were more likely to receive a test from a CHW. Confidence in AL treatment (Adj.OR=**2.75,** 95%CI:1.54-4.92) and perceived accuracy of an RDT performed by a CHW (Adj.OR **=2.43,** 95%CI:1.12-5.27) were positively associated with testing by a CHW. Specific CHW attributes were associated with higher testing rates including formal employment (Adj.OR= 1.73, 95%CI:0.05-2.70), those serving more than 50 households (Adj.OR = 1.73, 95%CI:0.70-2.74) and those serving areas with a higher test positivity (Adj.OR, 2.14, 95%CI:1.05, 3.22). On both the supply side and the demand side,

For peer review only - http://bmjopen.bmj.com/site/about/guidelines.xhtml

confidence in a test performed by a CHW was strongly associated with the success of the intervention.

Conclusion: Scale-up of community-based malaria testing is feasible and effective at increasing access among the poorest households. To maximize the impact of such interventions, it is important to recognize factors that may restrict both delivery and demand for such services.

Strengths and Limitations

1. **This analysis draws on a large and multidimensional dataset describing uptake of community-based services and delivery of services in the context of a cluster-randomized implementation trial**
2. **These data shed light on the population groups that were successfully reached with the intervention and those that may require alternative approaches in order to achieve adequate coverage of diagnostic testing before treatment with antimalarials. They also suggest that optimal service delivery by CHWs requires attention to their workload and community awareness about the quality of services provided.**
3. **The results also show that household beliefs about the intervention signficantly influence how people interact with the intervention.**
4. **As a secondary analysis of trial data, we were limited in the scope of the data available and not able to fully explore the interaction between the CHWs and households nor the complex determinants of health seeking behavior.**

For peer review only

Background

Despite decades of control efforts, malaria remains a major public health problem. In 2019, an estimated 229 million cases of malaria occurred worldwide. Most malaria cases in 2019 were in the World Health Organization (WHO) African Region (215 million or 94%). In the same year, there were an estimated 409 000 deaths from malaria globally, with Africa again accounting for 94%. Children under 5 years are the most vulnerable group affected by malaria and in 2019, 67% of malaria deaths were in this group (1).

Prompt malaria diagnosis either by microscopy or rapid diagnostic tests (RDTs) is recommended by WHO for all patients with suspected malaria before they are given treatment (2). Already, 95 countries have adopted the policy of testing all patients with suspected malaria before being treated, and this number is increasing. Diagnostic testing is free of charge in the public sector of almost 90 countries(3). Early and accurate diagnosis is essential both for effective management of the disease and for strong malaria surveillance. Parasite-based diagnostic testing significantly reduces illness and death by enabling health providers to swiftly distinguish between malarial and non-malarial fevers and select the most appropriate treatment(4).

The Kenya National Control Program adopted the Test and Treat policy in (2010) and this has been cascaded to the community level. RDTs are recommended for parasitological diagnosis in public health facilities where microscopy is not available and through Community Health Workers in some regions. Despite these efforts, most people in rural Kenya still opt for self-treatment and seek care in the retail health sector(5,6). Point-of-care diagnostic testing is not available in pharmacies and chemists, therefore patients who purchase antimalarials in the retail sector are not diagnosed before treatment. Studies have shown that targeting of antimalarials to true malaria

For peer review only

For peer review only - http://bmjopen.bmj.com/site/about/guidelines.xhtml

cases in the retail sector is very low; as few as 20% of people purchasing antimalarials are suffering from malaria (7,8) and as high as 70% of people with malaria do not purchase an ACT(9).

The deployment of RDTs through trained community health workers could improve access to diagnosis for populations with limited access to health facilities. We evaluated an intervention that targeted individuals who seek care in the retail sector by offering free community-based malaria diagnostic testing through CHWs. Following testing, those with a positive test result received a voucher to obtain first-line antimalarials at a nearby pharmacy at a heavily subsidized price. The intervention demonstrated a significant improvement in testing before treatment (6).

Ideally, there should be adequate demand for the commodity or opportunity, and supply should be able to meet demand. In our previous study, only 55 percent of those with recent illness reported having a malaria test before taking drugs and 21% still purchased antimalarials over-the-counter without a test (6), representing substantial scope for further improving the reach and impact of this intervention.

Here we report a secondary analysis of data collected during a large cluster randomized trial of community-based diagnostic testing in western Kenya. We sought to understand what factors might increase or decrease demand for testing from the community, compared to factors that may have limited supply of testing by CHWs. A comparison of constraints on the demand side and supply side could help understand the conditions under which the intervention operated well or was hindered by a mismatch in supply and demand. The results will be instrumental for improving the design of community-based interventions delivered at scale.

Methods

Description of the main trial

For peer review only - http://bmjopen.bmj.com/site/about/guidelines.xhtml

The purpose of this study was to encourage clients to receive testing for malaria before seeking treatment. This was accomplished by training and equipping community health workers (CHWs) to perform rapid-diagnostic tests (RDTs) for malaria and to provide clients who test positive for malaria with a voucher that allows them access to subsidized high-quality antimalarial drugs through participating pharmacies. (6)

This was a cluster-randomized controlled trial in 32 community clusters in western Kenya (population approximately 160,000). Eligible clusters had retail outlets selling ACTs and existing community health worker (CHW) programs and were randomly assigned 1:1 to control and intervention arms. In intervention areas (comprised of 16 community units), CHWs were available in their villages to perform malaria rapid diagnostic tests (RDTs) on demand for any individual >1 year of age experiencing a malaria-like illness. Malaria RDT-positive individuals received a voucher for a discount on a quality-assured ACT, redeemable at a participating retail medicine outlet. In control cluster (composed of 16 community units), CHWs offered a standard package of health education, prevention, and referral services. We conducted 4 population-based surveys—at baseline, 6 months, 12 months, and 18 months—of a random sample of households with fever in the last 4 weeks to evaluate predefined, individual-level outcomes. The primary outcome was uptake of malaria diagnostic testing at 12 months. The main secondary outcome was rational ACT use, defined as the proportion of ACTs used by test-positive individuals.

CHWs work on a volunteer basis, while CHEWs are employees of Kenya's Ministry of Health (MOH). Though there are no formal education requirements for CHWs, the ability to read and write are criteria for selection by the MOH. The role of CHWs as defined by the MOH is to promote good health through providing health education, basic treatments (such as first-aid), and referrals to health care facilities. The CHWs are not paid for their involvement in this malaria study, but

For peer review only - http://bmjopen.bmj.com/site/about/guidelines.xhtml

receive some compensation via mobile phone airtime and a small performance-based biannual bonus given to the cluster-specific CHW association.

CareStart HRP2-based malaria RDTs were used for testing. CareStart HRP2 detects *Plasmodium falciparum* which is the main species of parasite responsible for the majority of malaria cases in Kenya (10). In addition to malaria RDT, the study team provided CHWs with the necessary supplies to conduct the RDT testing. CHWs were free to perform tests in the location(s) of their choice (e.g., in their own homes, at clients' homes, etc.), as long as the location allowed for proper and sufficiently private performance of the procedure.

The CHWs tested febrile patients at community level and issued ACT vouchers to patients with positive results. The voucher redemption period was three days where by after that the Medicine shops were not allowed to collect the voucher. Patients with positive malaria results bought ACTs from medicine shops by redeeming their vouchers and leaving the voucher at the shop. The study team collected the redeemed vouchers from the medicine shops on a bi-weekly basis and reimbursed the difference in costs.

Community surveys

A community-based survey was conducted to collect individual-level study outcomes based on a population-based survey sampling strategy. This was a repeated cross-sectional household survey at 4 time points: baseline (pre-intervention), 6 months, 12 months, and 18 months post baseline. The survey was conducted in all the 32 community units, 16 Intervention and 16 control. Households were randomly selected by systematic random sampling and one person with fever in the last four weeks was interviewed in any household. Details of community surveys sample

For peer review only

For peer review only - http://bmjopen.bmj.com/site/about/guidelines.xhtml

size calculation are described elsewhere(11) . Briefly, the target sample size of eligible individuals with a fever in the last 4 weeks was 640 per arm (i.e., 40 per CU) at each time point, which—assuming 22.2% of households would have an eligible member—would require contacting 5,766 households. The starting point (index house) and sampling interval were different in each survey round in order to minimize repeat surveys across multiple rounds. We restrict our analysis here to the 12 and 18 month surveys in order to evaluate the effects of interest in the time-period when the intervention effect could be measured (12).

Basic demographic information about the participants and the households were collected. Socioeconomic scores were calculated from an asset index and then grouped into quintiles as described previously. Euclidean distance from a household to the nearest health facility was calculated from GPS coordinates. In the final survey, participants beliefs about their illness, malaria risk, diagnostic testing, and treatment were elicited by asking them about the severity of their illness, their confidence in positive test result and a negative test result, and how certain they were that they would recover from malaria after treatment with AL. "Perceived prevalence" was defined as the proportion of fevers that the respondent believed would be due to malaria among 10 people with fever from their village. High perceived prevalence was assigned to those who responded 8-10 out of 10, medium prevalence to those who responded 4-7 out of 10 and low to those who responded 0-3 out of 10.

Midpoint CHW Survey

As part of the process evaluation, at the midpoint of this study, interviews were conducted with a random sample of 70 CHWs who were trained to conduct RDTs (intervention CUs). The primary objective of these interviews were to determine the CHWs' satisfaction with their role in the malaria project and the feasibility of their continued involvement. Secondary objectives included

For peer review only

eliciting the main challenges the CHWs have faced in implementing the study, determining unanticipated burdens on the CHWs' other activities (including their responsibilities as CHWs outside of the study as well as their non-CHW responsibilities) and identifying changes in the types of services they provide and the recipients of their services(13).

Likert-scale questions were also asked, in which the interviewers read a statement aloud and the CHWs were asked to respond how much they agreed with the statement on a five-point scale ("strongly agree", "somewhat agree," "neutral," "somewhat disagree," "strongly disagree"). For this analysis, several variables were derived from the survey data. The client score was constructed as a composite score based on four likert scale questions – whether CHWs agreed that; clients trust the results of a positive RDT by the CHW, they trust a negative RDT, they follow the CHWs advice if the test was positive, they follow advice if the test was negative. A response of 'strongly agree'=2, somewhat agree='1' and neutral/somewhat disagree/strongly disagree=0. If the sum of these four responses was 6 or greater, the score was 'high'. If less than 6, the score was 'low'.

These same CHWs also performed an RDT under observation and were scored on each step. Those who completed at least 17 of 20 steps correctly had a 'high' score for RDT performance and <17 had a 'low' score.

Demand and Supply

To investigate factors that might have influenced the success of the intervention, the analysis was divided into demand and supply factors. On the demand side, the analysis investigates factors that might influence the community member's outcome of having a test, first from any source and then from the CHW. Only respondents with complete data on the variables of interest were included. On the supply side, the analysis investigates the factors associated with the testing

For peer review only - http://bmjopen.bmj.com/site/about/guidelines.xhtml

output (supply) at the CHW level, measured as the monthly number of tests conducted by each CHW. These factors include; socio-demographic characteristics of the CHW, number of households under the CHWs care, years of experience of the CHW, the number of other activities the CHW engages in under their role as a CHW (under the assumption that an increasing number of activites may compete for their time, the CHW provided a list of activities and these were summed together), the perception of the intervention by the CHW, the skill of the CHW in conducting RDTs and the overall prevalence of malaria in their community.

Outcome Measures

This analysis looks at the clusters in the intervention arms. The primary outcome is the uptake of testing, defined as the proportion of fevers in the previous 4 weeks that receive a diagnostic test from any source. The second primary outcome is the uptake of testing from a community health worker among those with any test. We present descriptive results for individuals getting tested from any source and from a CHW across the intervention clusters.

The secondary outcome was to investigate the testing volume and the factors influencing this. This was measured by the mean monthly tests for each CHW. Monthly number of tests per CHW was available from program data collected at monthly supervision visits.

Statistical Analysis

A mixed-effects logistic regression analysis was used to evaluate the primary outcome measures on the uptake of testing and investigate risk factors that might have contributed to the outcomes of the intervention reported in (12). The hierarchical nature of the data informed the adoption of multi-level modelling, since we include both individual-, and CHW- level risk factors in the analysis

For peer review only - http://bmjopen.bmj.com/site/about/guidelines.xhtml

For peer review only

and account for clustering at the CU-level. The outcomes were evaluated only for the CUs in the intervention arm and summaries provided.

For the primary outcome measures to evaluate the uptake of testing, two logistic models were generated; the first model included all individuals tested at any source as the outcome and the second model included a subset of this population, those tested by a CHW as the outcome. For both models, we accounted for clustering at the CU level, and then included potential individual-level covariates like respondent's age, gender, level of educational, occupation, socio-economic status, geographic proximity to health care provider, the time it took to obtain the malaria test, the level of knowledge and opinions about malaria and malaria diagnosis.

The logistic regression model is as shown below:

$$log\left(\frac{p_{ij}}{1-p_{ij}}\right) = \beta_0 + \beta_1 X_{1j} + \ldots + \beta_m X_{mj} + u_j$$

where p_{ij} is the probability of the outcome for each individual i in cluster j, β_0 is the model intercept, β_{1-m} are the coefficients for each potential risk factor X_{1-m}, and u_j represents the random effects for cluster j.

Fixed effects are measured using an Odds Ratio (OR) and included the 95% confidence intervals. Independent categorical variables were screened for multicollinearity with a Cramér's V statistic. In case two variables showed signs of correlation (V > 0.7), the variable least associated with the response variable was omitted (14).

For the secondary outcome measure, a linear regression model adjusted for clustering at the CU level was used. This model investigates the outcome of testing volume by the CHWs and includes

For peer review only - http://bmjopen.bmj.com/site/about/guidelines.xhtml

potential CHW-level covariates like CHW age, years of experience/workload, number of households responsible, number with previous CHW experience, number of hours spent on community health, and training in malaria case management or RDTs.

The linear regression model is as shown below:

$$Y_{ij} = \beta_0 + \beta_1 X_{1j} + \ldots + \beta_m X_{mj} + u_j + \varepsilon_{ij}$$

where Y_{ij} is the outcome for each individual i in cluster j, β_0 is the model intercept, β_{1-m} are the co-efficients for each potential risk factor X_{1-m}, u_j represents the random effects for cluster j, and ε_{ij} is the error term.

All statistical analysis was done in STATA version 15 (StataCorp).

Patient and Public Involvement

No patients involved

For peer review only - http://bmjopen.bmj.com/site/about/guidelines.xhtml

Results

A total of 292 CHWs in 16 Intervention CUs were trained to perform RDTs and 36 retail shops were enrolled to redeem vouchers. The intervention was launched on July 21, August 17, and September 22, 2015 in Bokoli, Kiminini, and Ndivisi sub-counties western Kenya, respectively and continued until May 5, 2017.

Demand side factors for use of malaria diagnostic testing

The community-based demand analysis relies on 1738 complete surveys from participants in randomly selected households in intervention clusters with an acute illness in the last four weeks (**Table 1**). Community members were interviewed at 12 or 18 months (47.2% and 52.8% respectively) following the initiation of the intervention. 55% (n= 948) of participants reported having a malaria test for their recent illness while 38.4% (n=364/948) of those tested reported having had the test at CHW. Among all surveyed participants, 41% (n= 710) were male and 59% (n= 1028) were female. 21.2% (n= 368) of the recent fevers were aged <5 years, 43.3% (n= 752) were 5 -17 years while 36% (n= 618) were 18> years. Only 21.4% (n= 371) of the participants had completed their secondary education while 45% (n= 778) had completed their primary education. On average, the distance to the nearest public health facility was approximately 2km while the nearest drug shop was approximately 1.3km away.

Table 1: Characteristics of community survey participants who reported malaria test in 12 months and 18 months.

Participant characteristics (N=1738)	(n)	%
Tested at any location	948	54.6
Tested at CHW	364	20.0
Patient gender		

For peer review only - http://bmjopen.bmj.com/site/about/guidelines.xhtml

Male	710	40.9
Female	1028	59.1
Patient age		
Under 5	368	21.1
5-17 y	752	43.3
18+y	618	35.6
Education level of respondent		
Not completed primary	589	33.9
Completed primary	778	44.8
Completed secondary	371	21.3
Survey timepoint		
12 months	821	47.2
18 months	917	52.8
Household characteristics (N=1738)	Mean	Standard deviation
Distance to the nearest public health facility, km	2.1	1.2
Distance to nearest drug shop, km	1.3	0.7

Demand-side factors that were associated with uptake of malaria testing for their most recent illness are examined (**Table 2**). Respondents who delayed seeking care beyond the first 24 hours had higher odds of receiving a test. Being aware of your local CHW increased the odds of having a test from any source, facility or CHW (Adj. OR=1.50, 95% CI: 1.10-2.04). Respondents from the wealthiest households were 53% more likely to have a test (Adj. OR=1.53 95%CI: 1.14-2.06). Being an adult at least 18 years old was negatively associated to taking a test (Adj. OR=0.59, 95% CI:0.43-0.82). Distance to a health facility greater than 2km (Adj. OR=0.86, 95% CI:0.67-0.11) and use of ITN (Adj. OR=1.27, 95%CI:0.93-1.74) were not significantly associated with testing uptake.

Table 2. Association between participant characteristics and malaria diagnostic testing. Adjusted and unadjusted odds ratios for receiving a malaria diagnostic test, either microscopy or RDT, from any source for the most recent febrile illness.

For peer review only - http://bmjopen.bmj.com/site/about/guidelines.xhtml

	Univariate analysis		Multivariate analysis	
Participant characteristics (N=1738)	Unadjusted Odds Ratio (95%CI)	P-value	Adjusted Odds Ratio (95%CI)	P-value
Time to seek care (*Reference=same day*)				
Next day	**1.21[1.05,1.42]**	**0.01**	**1.23[1.06,1.43]**	**0.01**
After two days	1.01[0.78,1.32]	0.94	1.13[0.86,1.48]	0.38
More than two days	0.94[0.73,1.22]	0.66	1.12[0.87,1.44]	0.37
Use an ITN *(Reference=does not use an ITN)*	1.37[0.99,1.92]	0.06	1.27[0.93,1.74]	0.13
Distance to public health facility >2km (*Reference = <2km*)	0.83[0.64,1.06]	0.14	0.86[0.67,1.11]	0.26
Aware of a CHW in their village *(Reference=is not aware of a CHW in their village)*	**1.43[1.01,2.05]**	**0.04**	**1.50[1.10,2.04]**	**0.01**
Patient age (*Reference=1-4y*)			-	-
5 to 17y	0.76[0.54,1.08]	0.12	0.79[0.56,1.12]	0.18
18+y	**0.59[0.43,0.81]**	**<0.01**	**0.59[0.43,0.82]**	**<0.01**
Patient is male (*Reference = female*)	1.02[0.86,1.23]	0.78	0.94[0.80,1.10]	0.43
Respondent education level (*Reference=none*)			-	-
Completed primary	1.12[0.93,1.36]	0.23	1.08[0.87,1.33]	0.48
Completed secondary	**1.41[1.04,1.90]**	**0.03**	1.25[0.93,1.70]	0.14
Wealth quintile (*Reference=0-20th*)			-	-
20 - 40th	1.04[0.68,1.62]	0.83	1.02[0.65,1.59]	0.94
40 - 60th	1.14[0.86,1.50]	0.36	1.08[0.82,1.43]	0.59
60 - 80th	1.10[0.79,1.53]	0.56	1.03[0.73,1.46]	0.86
>80th	**1.59[1.15,2.21]**	**<0.01**	**1.53[1.14,2.06]**	**<0.01**

The analysis further focused on those who were tested for malaria by a CHW **(Table 3).** Out of 948 participants who reported having a test, 38.4% reported having taken a test from a CHW (364/948). It is noted that wealthier participants prefer being tested at the health facility (Adj. OR

For peer review only

for testing at a CHW=0.32, 95%CI:0.17-0.60), and those with education above secondary school have lower odds of taking a test at the CHW (Adj. OR=0.68, 95%CI:0.44-1.07). Delaying testing beyond two days also reduced the odds of testing by a CHW. On the other hand, school-aged children between 5-17 years were more than twice as likely to be tested by a CHW compared to those below 5 years old (Adj. OR=2.39, 95%CI: 1.43-4.01).

Table 3: Association between patient characteristics and having an mRDT from a CHW before taking any treatment.

	Univariate analysis		Multivariate analysis	
Participant Characteristics of those who took a test from CHW (n=948)	Unadjusted Odds Ratio (95%CI)	P-value	Adjusted Odds Ratio (95%CI)	P-value
Time to malaria test (*Reference=same day*)			-	-
Next day	0.96[0.71,1.31]	0.80	0.96[0.69,1.33]	0.81
After two days	0.73[0.44,1.21]	0.22	0.68[0.42,1.10]	0.12
More than two days	0.81[0.49,1.34]	0.41	0.87[0.50,1.52]	0.62
Use an ITN *(Reference=does not use an ITN)*	**0.57[0.41,0.78]**	**<0.01**	0.77[0.55,1.06]	0.11
Distance to public health facility>2km (*Reference = <2km*)	1.32[0.80,2.20]	0.28	1.29[0.82,2.03]	0.27
Patient age (*Reference=1-4y*)			-	-
5 to 17y	**2.43[1.49,3.95]**	**<0.01**	**2.39[1.43,4.01]**	**<0.01**
18+y	0.98[0.67,1.43]	0.91	1.09[0.72,1.66]	0.68
Patient is male (*Reference = female*)	1.23[0.98,1.54]	0.07	1.02[0.82,1.26]	0.85
Respondent education level (*Reference=none*)			-	-
Completed primary	0.86[0.69,1.09]	0.22	0.96[0.75,1.22]	0.74
Completed secondary	**0.45[0.29,0.68]**	**<0.01**	**0.68[0.44,1.07]**	**0.10**
Wealth quintile (*Reference=0-20th*)			-	-
20 - 40th	1.13[0.65,1.94]	0.671	1.11[0.63,1.96]	0.72
40 - 60th	0.83[0.63,1.09]	0.175	0.79[0.58,1.05]	0.11

For peer review only - http://bmjopen.bmj.com/site/about/guidelines.xhtml

60 - 80th	0.67[0.47,0.96]	0.028	0.66[0.45,0.97]	0.03
>80th	**0.26[0.13,0.53]**	**<0.001**	**0.32[0.17,0.62]**	**<0.01**

To understand how individual perceptions about malaria testing and treatment, malaria risk, and illness severity affected uptake of testing or testing at a CHW, the analysis focused on the final survey at 18 months survey **(Table 4)**. After adjusting for demographic characteristics, patient perceptions were differentially related to testing overall and testing specifically with a CHW. Both confidence in AL treatment (Adj. OR=2.75, 95% CI: .1.54-4.92) and confidence in the accuracy of an RDT performed by a CHW (Adj. OR=2.43, 95% CI: 1.12-5.27) were strongly positively associated with testing at a CHW. Those who reported their illness as severe were more likely to be tested (Adj. OR=2.37, 95% CI: 1.58-3.58) but had a lower odd of testing at a CHW (Adj. OR=0.44, 95% CI: 0.22-0.87). Confidence in an RDT result, either positive or negative was not significantly associated with receiving a test for a recent illness, but those who reported a high confidence in a negative RDT result had substantially higher odds of testing with a CHW than in a facility, although this did not reach statistical significance at the 95% level (Adj OR=1.58, 95%CI: 0.89-2.83). Finally, those who reported a moderate perceived prevalence in their village (ie 4-7 fevers out of 10 would be due to malaria) or high perceived prevalence (ie 8-10 out of 10 fevers would be due to malaria) had increasingly higher odds of receiving a test from a CHW (medium prevalence Adj. OR=2.52, 95%CI: 1.26-5.06, high prevalence Adj. OR=2.70, 95% CI: 1.17-6.22).

Table 4: Relationship between participant beliefs and perceptions and uptake of testing from any source or from a CHW. Models are adjusted for patient age, gender, household wealth quintile and the education level of the respondent in addition to the variables in the table.

For peer review only - http://bmjopen.bmj.com/site/about/guidelines.xhtml

Had a malaria test (N=703)	Univariate analysis		Multivariate analysis	
	Unadjusted Odds Ratio (95%CI)	P-value	Adjusted Odds Ratio (95%CI)	P-value
Confidence in a positive RDT result	1.02[0.67,1.54]	0.93	0.87[0.53,1.41]	0.57
Confidence in a negative RDT result	1.34[0.87,2.03]	0.19	1.24[0.72,2.14]	0.43
Confidence in AL treatment	1.27[0.94,1.71]	0.12	1.06[0.81,1.40]	0.63
Trust an RDT by a CHW as much as one at a facility	0.85[0.55,1.32]	0.48	**0.66[0.44,0.97]**	**0.03**
Illness is severe	**2.36[1.64,3.39]**	**<0.001**	**2.37[1.58,3.58]**	**<0.001**
Prevalence (*Reference= low*)			-	-
Medium	1.51[0.89,2.57]	0.13	1.48[0.85,2.58]	0.16
High	1.12[0.70,1.80]	0.64	1.05[0.63,1.77]	0.85
**Had a test with a CHW (N=428)**	Univariate analysis		Multivariate analysis	
	Unadjusted Odds Ratio (95%CI)	P-value	Adjusted Odds Ratio (95%CI)	P-value
Confidence in a positive RDT result	1.45[0.89,2.34]	0.13	1.04[0.64,1.71]	0.86
Confidence in a negative RDT result	1.61[0.99,2.64]	0.06	1.58[0.89,2.83]	0.12
Confidence in AL treatment	**2.67[1.52,4.70]**	**<0.01**	**2.75[1.54,4.92]**	**<0.01**
Trust an RDT by a CHW as much as one at a facility	**2.91[1.29,6.58]**	**0.01**	**2.43[1.12,5.27]**	**0.03**
Illness is severe	0.60[0.35,1.03]	0.06	**0.44[0.22,0.87]**	**0.01**
Prevalence (*Reference= low*)			-	-
Medium	**2.87[1.48,5.56]**	**<0.01**	**2.52[1.26,5.06]**	**0.01**
High	**2.85[1.31,6.23]**	**0.01**	**2.70[1.17,6.22]**	**0.02**
Confidence in a positive RDT result	1.45[0.89,2.34]	0.13	1.04[0.64,1.71]	0.86

Diagnostic testing by CHWs

In total, 32,404 RDTs were conducted by the CHWs over the intervention period and 33.7% (n= 10,870) percent were positive. All RDT interpretations were counterchecked by the study team.

For peer review only

Those with a positive test received a voucher for a discounted quality-assured ACT. These vouchers were redeemed at a participating outlet by 93.9% of voucher recipients. There was no instances of stock out of RDTs among our CHWs. To explored factors that may be associated with the CHWs ability to conduct RDTs, we examined the association between testing volume, defined as the mean number of RDTs conducted per month, and the different CHW demographic characteristics (**Table 5**). Of the 244 trained CHWs, 147 (60.3%) were above 40 years of age, 72 (29.5%) were male. There were 107 (43.8%) CHWs who had not completed secondary education.

Table 5: Association between mean number of tests performed per month and CHW characteristics

Mean number of tests per month	4.60 (SD=3.07)	**Univariate Analysis**		**Multivariate Analysis**	
		**N = 244**		**N = 244**	
CHW characteristics	n (sample proportion)	Coeff (95%CI)	P-value	Coeff (95%CI)	P-value
Male	72 (0.295)	-0.30 [-1.28,0.68]	0.52	-0.28 [-1.24,0.68]	0.54
Age <40 years *(Reference)*	97 (0.397)	-	-	-	-
> = 40 years	147 (0.603)	-0.21 [-1.06,0.63]	0.60	-0.42 [-1.12,0.28]	0.23
Married *(Reference = unmarried/divorced)*	202 (0.828)	0.37 [-0.57,1.30]	0.42	0.22 [-0.92,1.35]	0.69
Education Below secondary *(Reference)*	107 (0.438)	-	-	-	-
Completed Secondary	137 (0.562)	0.45 [-0.33,1.23]	0.24	0.23 [-0.40,0.87]	0.44
Formal Employment *(Reference = no formal employment)*	33 (0.135)	**1.13 [-0.01,2.28]**	**0.05**	**1.37 [0.05,2.70]**	**0.04**
Previously trained in mRDT	46 (0.188)	-0.46 [-1.64,0.72]	0.42	**-1.40 [-2.44, -0.37]**	**0.01**
Households[1] <50 *(Reference)*	134 (0.549)	-	-	-	-
50-100	66 (0.271)	**2.14[0.63,3.64]**	**<0.01**	**1.73[0.70,2.74]**	**<0.01**
>100	44 (0.180)	1.18[-0.40,2.76]	0.13	**1.49[0.74,2.24]**	**<0.01**

For peer review only - http://bmjopen.bmj.com/site/about/guidelines.xhtml

Cluster-level test positivity[2] Low (≦25%; *Reference*)	141 (0.588)	-	-	-	-
High (>25%)	103 (0.422)	**2.54[0.97,4.10]**	**<0.01**	**2.14[1.05,3.22]**	**<0.01**

[1] Number of households for which the CHW reported being responsible

[2] Percentage of mRDTs conducted by the CHWs in that cluster which turned positive over the study period.

In a univariate and multivariate analysis, formal employment, previous RDT training, number of households and RDT-positivity rate at the cluster-level were associated with volume of tests done. Sex, age, being married and education level were not significantly associated with testing volume. CHWs who were formally employed performed on average 1.37 more tests per month (Adj coefficient=1.37, 95% CI: CI: 0.05-2.70) but those who reported being trained previously on RDTs performed 1.4 fewer tests per month (Adj coefficient=1.4, 95%CI: -2.44,-0.37). CHWs serving areas with a high proportion of positive tests (proportion RDT +ve >25%) tested on average 2 more clients per month than those in lower prevalence areas (Adj coefficient= 2.14, 95%CI: 0.63-3.64). CHWs who were responsible for at least 50 households tested more clients (Adj coefficient=1.73, 95%CI: 0.70,2.74), although this did not increase further with when the number of households exceeded 100 (Adj coefficient = 1.49, 95%CI: 0.74-2.24).

A univariate and multivariate analyses to test the association between testing volume and CHW perceptions about their role at the midpoint survey (**Table 6**). High client score was significantly associated with higher testing volume performed by CHWs who perceived that their clients trusted their tests (Adj. coefficient=1.37, 95%CI: 0.11-2.62). The CHWs competency at conducting all 20 steps of the RDT process (RDT score), the number of different activities to which CHWs were committed, whether they named RDT testing as their most important activity, or whether they

For peer review only

For peer review only - http://bmjopen.bmj.com/site/about/guidelines.xhtml

cited extrinsic motivation (money, airtime, or other) as important reasons for continuing their work were all not significantly associated with average tests performed per month.

Table 6: Association between testing volume and CHW perceptions about their role at the midpoint survey. Multivariate odds ratios are adjusted for age, gender, education level, formal employment. The analysis includes a random sample of CHWs selected for midpoint evaluation.

	n (sample proportion)	**Univariate Analysis**		**Multivariate Analysis[1]**	
CHW characteristics	N=70	Coeff (95%CI)	P-value	Coeff (95%CI)	P-value
RDT Score[2] (*Reference=Low*)	-	-	-	-	-
High	54 (0.771)	0.19[-1.25,1.64]	0.78	0.11[-1.34,1.57]	0.87
Number of different activities last month (*Reference*=<=3)	-	-	-	-	-
> 3 activities	19 (0.271)	0.35[-1.56,2.26]	0.70	-0.45[-2.04,1.15]	0.56
Most important activity is malaria testing	48 (0.686)	0.88[-0.25,2.01]	0.12	0.07[-1.16,1.32]	0.90
Cited extrinsic motivators as important[3]	28 (0.400)	0.33[-1.52,2.17]	0.71	0.67[-0.92,2.25]	0.38
Client Score[4] (*Reference= Low*)	-	-	-	-	-
High	48 (0.686)	**1.46[0.10,2.83]**	**0.04**	**1.37[0.11,2.62]**	**0.04**
Years of experience as a CHW (*Reference = <=5y*)	-	-	-	-	-
>5 years	38 (0.543)	-0.35[-2.68,1.98]	0.75	-0.51[-2.56,1.55]	0.61

[1] Multivariable models are also adjusted for CHW demographic factors (age, gender, education, employment, and cluster-level prevalence)
[2]RDT score - CHW scored > 17 correct steps on a 20-step checklist for RDT preparation and interpretation conducted 6 months after training
[3] CHW cited money, non-monetary incentives or desire for recognition as important motivators for their role as a CHW
[4] Client score was derived from four questions where the CHW reported how confident clients were in the testing they provided.

For peer review only - http://bmjopen.bmj.com/site/about/guidelines.xhtml

Discussion

This study adds to the evidence that it is feasible for CHWs to offer malaria testing using RDTs in the community. With the use of CHWs, large-scale malaria testing intervention can reach the underserved population who cannot afford to visit the health facilities for care and those who seek care in the retail sector. The existence of CHWs with the ability to test for malaria using RDT provides a great opportunity, however, various factors may affect the demand and supply of testing using RDT by CHWs.

Here we examine the outcomes of a community-based malaria testing to understand how to improve intervention design and maximize the impact of such programs. The analysis was organized around the target group (community members) and their uptake or demand for the diagnostic testing intervention, and the supply of testing by the CHWs responsible for delivering the intervention. We were able to separate the determinants of uptake of malaria diagnostic testing generally from the determinants of uptake of malaria rapid diagnostic test performed by a CHW. These contrasting but complementary analyses helped us identify bottlenecks to intervention coverage at the community level.

Analysis of community survey data revealed that increased demand for malaria diagnostic testing before treating a fever is positively associated to education level and wealth, but lower among adults, findings which are consistent with other published studies (15–18). We also find that being aware of the local CHW increased demand for testing. This is similar to other studies that have evaluated the contribution of CHWs to population health-seeking behavior(17,19). In the context of our program, this is especially important because the testing was initiated by the community

For peer review only - http://bmjopen.bmj.com/site/about/guidelines.xhtml

member seeking out a CHW rather than a door-to-door campaign initiated by the CHW. Malaria testing by a CHW provides an opportunity for reducing health disparities by ensuring the poor and underserved population get access to testing and are able to make an informed choice on how to manage their illness. Our results show that community members with a lower socioeconomic score were more likely to receive a test from a CHW. Malaria testing by the CHW was free and this finding therefore aligns well with the intention of the intervention. Testing by a CHW was also more common among those with high confidence in the reliability of a test done by a CHW and also among those with high confidence in artemether-lumefantrine, the treatment offered through the CHW testing program. Taken together, these results suggest that the community awareness of and perception of the CHW is key in improving demand. In contrast, although testing was high among those who considered their illness to be very severe, these individuals were less likely to be tested by a CHW. This result is appropriate and consistent with the goals of the program which included immediate referral of more severe cases.

From the perspective of CHWs delivering malaria testing, a strong association was not observed between demographic characteristics and testing volume, but we did note that CHWs with concurrent formal employment tested more patients on average than those without which could indicate greater visibility or mobility in the community. It was also observed that the monthly testing volume increased when the number of households under a CHWs purview exceeded 50, but did not increase further when households exceeded 100, suggesting a threshold effect whereby more households created more demand for testing but the ability to meet the demand is saturated after some threshold beyond 100 households. Finally, we report that testing rates were higher in clusters with high RDT-positivity rates, possible reflecting a greater awareness of malarial illness or higher demand for malaria services in areas with higher malaria burden. This could also reflect

For peer review only - http://bmjopen.bmj.com/site/about/guidelines.xhtml

higher confidence in a test among CHWs or communities members who have experience with some tests turning positive as has been seen in the healthcare setting (20).

A deeper analysis of CHW perception of the intervention was undertaken in a smaller random sample of participating CHWs. We show that testing volume is not affected by competing CHW-related priorities, but is associated with how they perceive their clients' confidence in testing and the likelihood of the client accepting the RDT result and advice. This aligns well with the picture from the demand-side where we observe that confidence in an RDT performed by a CHW is strongly associated to testing with a CHW.

This study had several limitations. First, we are not able to estimate the effect of free testing on the uptake of testing. Considering the study utilized diagnosis dependent subsidies to test the rational use of ACT, a similar study that deploys free testing in the absence of a conditional voucher would be required to resolve this. Second, although the survey data has provided valuable insight on understanding drivers to demand and supply of malaria testing, seeking provider (CHW) and patient perspectives could provide a richer understanding of the facilitating factors and barriers to scaling up such an intervention, particularly the ways in which preferences and changes in behavior may influence uptake of the intervention.. Finally, though our survey design used repeated cross-sectional sampling, it is possible that the same household and/or febrile individual was surveyed at multiple survey time points and any resulting association was not explicitly modeled in our regression analysis.

Conclusion

For peer review only - http://bmjopen.bmj.com/site/about/guidelines.xhtml

In summary, scale up of health interventions through CHWs is feasible and effective at reaching the poorest households. However, in order to maximize the impact of community-based interventions, it is important to recognize factors that may restrict both delivery and demand for such services. It is critical to not only create awareness of CHW services but foster trust in both the CHWs ability to deliver services and the health intervention itself. Engendering confidence in the CHWs proficiency is likely to have a synergistic effect on both demand for services and commitment to delivering services.

Declarations

Ethics approval and consent to participate: Ethical approval was granted by Moi University Institutional Research and Ethics Committee (*Formal Approval No. 0001403*) and Duke University Institutional Review Board (*Pro00063384*). Written informed consent was obtained from participants during household interviews and from CHWs prior to collecting midpoint evaluation data. All study methods were carried out in accordance with ethical guidelines and regulations set out by both Moi University and Duke Ethical Review Boards.

Consent to publish: Not applicable

Availability of data and materials: The datasets used and/or analysed during the current study are available from the corresponding author on reasonable request.

Competing interests: The authors declare that they have no competing interests

Funding: This study was supported from the National Institute of Allergy and Infectious Diseases of the National Institutes of Health (US) (grant number R01AI110478). The funders had no role in study design, data collection and analysis, decision to publish, or preparation of this manuscript.

For peer review only

The content is solely the responsibility of the authors and does not necessarily represent the official views of the National Institute of Allergy and Infectious Diseases or the National Institutes of Health.

Authors' contributions:

Joseph Kipkoech – Conceptualization, writing original draft, revision and editing final draft

Josephine Malinga – Data curation, writing original draft, revision and editing final draft

Edna Sang - writing original draft, writing original draft, revision and editing final draft

George Ambani – Data collection, writing original draft, revision and editing final draft

Lucy Abel - Data collection, writing original draft, revision and editing final draft

Erick Nalianya - Data collection, writing original draft, revision and editing final draft

Jane Namae - Data collection, writing original draft, revision and editing final draft

Matthew Boyce - Review and editing final draft

Jeremiah Laktabai – Conceptualization, review and editing final draft

Diana Menya - Conceptualization, review and editing final draft

Wendy Prudhomme-O'Meara – Conceptualization, funding acquisition, writing original draft, revision and editing final draft

Acknowledgements: This study could not have been carried out without the exceptional attention to detail and commitment of our field team that include: I. Khaoya, L. Marango, E. Mukeli,

For peer review only - http://bmjopen.bmj.com/site/about/guidelines.xhtml

L. Nukewa, E. Wamalwa, and A. Wekesa. We are grateful to the study participants and the community health workers as well as the local community leadership that supported this work. We would also like to acknowledge the partnership and support of our Health Management Team colleagues in each subcounty and in particular V. Kiplagat, A. Kyalo, S. Malenya, and P. Musita.

For peer review only

For peer review only - http://bmjopen.bmj.com/site/about/guidelines.xhtml

References

1. World Health Organization. World malaria report 2020: 20 years of global progress and challenges. Geneva; 2020.

2. World Health Organization, Global Malaria Programme. Guidelines for the treatment of malaria. Geneva;. 2015. Available from: http://www.ncbi.nlm.nih.gov/books/NBK294440/ (accessed Jan 20, 2021)

3. World Health Organization. World malaria report 2019. Geneva; 2019.

4. WHO Guidelines for malaria. Geneva: World Health Organization; 2021. Available from: https://www.ncbi.nlm.nih.gov/books/NBK568494/

5. ACTwatch Group, Musuva A, Ejersa W, Kiptui R, Memusi D, Abwao E. The malaria testing and treatment landscape in Kenya: results from a nationally representative survey among the public and private sector in 2016. Malar J. 2017 21;16(1):494.

6. Prudhomme O'Meara W, Menya D, Laktabai J, Platt A, Saran I, Maffioli E, et al. Improving rational use of ACTs through diagnosis-dependent subsidies: Evidence from a cluster-randomized controlled trial in western Kenya. PLoS Med. 2018;15(7):e1002607.

7. Cohen J, Dupas P, Schaner S. Price Subsidies, Diagnostic Tests, and Targeting of Malaria Treatment: Evidence from a Randomized Controlled Trial. Am Econ Rev. 2015 Feb;105(2):609–45.

8. Briggs MA, Kalolella A, Bruxvoort K, Wiegand R, Lopez G, Festo C, et al. Prevalence of Malaria Parasitemia and Purchase of Artemisinin-Based Combination Therapies (ACTs)

For peer review only

For peer review only - http://bmjopen.bmj.com/site/about/guidelines.xhtml

among Drug Shop Clients in Two Regions in Tanzania with ACT Subsidies. PLOS ONE. 2014 Apr 14;9(4):e94074.

9. Mbonye AK, Lal S, Cundill B, Hansen KS, Clarke S, Magnussen P. Treatment of fevers prior to introducing rapid diagnostic tests for malaria in registered drug shops in Uganda. Malar J. 2013 Apr 16;12(1):131.

10. World Health Organization, Special Programme for Research and Training in Tropical Diseases, Foundation for Innovative New Diagnostics, Centers for Disease Control (U.S.). Malaria rapid diagnostic test performance: results of WHO product testing of malaria RDTs : 4 (2012). [Internet]. Geneva, Switzerland: World Health Organization on behalf of the Special Programme for Research and Training in Tropical Diseases; 2012 [cited 2021 Jun 7]. Available from: http://apps.who.int/iris/bitstream/10665/77748/1/9789241504720_eng.pdf

11. Laktabai J, Lesser A, Platt A, Maffioli E, Mohanan M, Menya D, et al. Innovative public-private partnership to target subsidised antimalarials: a study protocol for a cluster randomised controlled trial to evaluate a community intervention in Western Kenya. BMJ Open. 2017 20;7(3):e013972.

12. O'Meara WP, Menya D, Laktabai J, Platt A, Saran I, Maffioli E, et al. Improving rational use of ACTs through diagnosis-dependent subsidies: Evidence from a cluster-randomized controlled trial in western Kenya. PLOS Med. 2018 Jul 17;15(7):e1002607.

13. Winn LK, Lesser A, Menya D, Baumgartner JN, Kipkoech Kirui J, Saran I, et al. Motivation and satisfaction among community health workers administering rapid diagnostic tests for malaria in Western Kenya. J Glob Health. 2018 Jun;8(1):010401.

For peer review only

For peer review only - http://bmjopen.bmj.com/site/about/guidelines.xhtml

14. Burnham KP, Anderson DR, Huyvaert KP. AIC model selection and multimodel inference in behavioral ecology: some background, observations, and comparisons. Behav Ecol Sociobiol. 2011 Jan 1;65(1):23–35.

15. Chibwana AI, Mathanga DP, Chinkhumba J, Campbell CH. Socio-cultural predictors of health-seeking behaviour for febrile under-five children in Mwanza-Neno district, Malawi. Malar J. 2009 Sep 24;8(1):219.

16. Cohen J, Cox A, Dickens W, Maloney K, Lam F, Fink G. Determinants of malaria diagnostic uptake in the retail sector: qualitative analysis from focus groups in Uganda. Malar J. 2015 Dec;14(1):89.

17. Kisia J, Nelima F, Otieno DO, Kiilu K, Emmanuel W, Sohani S, et al. Factors associated with utilization of community health workers in improving access to malaria treatment among children in Kenya. Malar J. 2012 Jul 30;11:248.

18. Mukanga D, Tibenderana JK, Peterson S, Pariyo GW, Kiguli J, Waiswa P, et al. Access, acceptability and utilization of community health workers using diagnostics for case management of fever in Ugandan children: a cross-sectional study. Malar J. 2012 May 24;11(1):121.

19. Win Han Oo, Gold L, Moore K, Agius PA, Fowkes FJI. The impact of community-delivered models of malaria control and elimination: a systematic review. Malar J. 2019 Aug 6;18(1):269.

For peer review only

20. Faust C, Zelner J, Brasseur P, Vaillant M, Badiane M, Cisse M, et al. Assessing Drivers of Full Adoption of Test and Treat policy for Malaria in Senegal. Am J Trop Med Hyg. 2015 Jul 8;93(1):159–67.

For peer review only

For peer review only - http://bmjopen.bmj.com/site/about/guidelines.xhtml

STROBE Statement—Checklist of items that should be included in reports of ***cross-sectional studies***

	Item No	Recommendation	Page No
**Title and abstract**	1	(*a*) Indicate the study's design with a commonly used term in the title or the abstract	1
		(*b*) Provide in the abstract an informative and balanced summary of what was done and what was found	2-3
**Introduction**			
Background/rationale	2	Explain the scientific background and rationale for the investigation being reported	4-5
Objectives	3	State specific objectives, including any prespecified hypotheses	5
**Methods**			
Study design	4	Present key elements of study design early in the paper	6
Setting	5	Describe the setting, locations, and relevant dates, including periods of recruitment, exposure, follow-up, and data collection	6-7
Participants	6	(*a*) Give the eligibility criteria, and the sources and methods of selection of participants	8
Variables	7	Clearly define all outcomes, exposures, predictors, potential confounders, and effect modifiers. Give diagnostic criteria, if applicable	10
Data sources/ measurement	8*	For each variable of interest, give sources of data and details of methods of assessment (measurement). Describe comparability of assessment methods if there is more than one group	*9-10*
Bias	9	Describe any efforts to address potential sources of bias	
Study size	10	Explain how the study size was arrived at	8
Quantitative variables	11	Explain how quantitative variables were handled in the analyses. If applicable, describe which groupings were chosen and why	10
Statistical methods	12	(*a*) Describe all statistical methods, including those used to control for confounding	11-12
		(*b*) Describe any methods used to examine subgroups and interactions	
		(*c*) Explain how missing data were addressed	
		(*d*) If applicable, describe analytical methods taking account of sampling strategy	11
		(*e*) Describe any sensitivity analyses	
**Results**			
Participants	13*	(a) Report numbers of individuals at each stage of study—eg numbers potentially eligible, examined for eligibility, confirmed eligible, included in the study, completing follow-up, and analysed	14
		(b) Give reasons for non-participation at each stage	
		(c) Consider use of a flow diagram	
Descriptive data	14*	(a) Give characteristics of study participants (eg demographic, clinical, social) and information on exposures and potential confounders	14
		(b) Indicate number of participants with missing data for each variable of interest	
Outcome data	15*	Report numbers of outcome events or summary measures	15, Table 1

For peer review only - http://bmjopen.bmj.com/site/about/guidelines.xhtml

Main results	16	(*a*) Give unadjusted estimates and, if applicable, confounder-adjusted estimates and their precision (eg, 95% confidence interval). Make clear which confounders were adjusted for and why they were included	Table 2-6
		(*b*) Report category boundaries when continuous variables were categorized	Tables
		(*c*) If relevant, consider translating estimates of relative risk into absolute risk for a meaningful time period	
Other analyses	17	Report other analyses done—eg analyses of subgroups and interactions, and sensitivity analyses	
**Discussion**			
Key results	18	Summarise key results with reference to study objectives	18
Limitations	19	Discuss limitations of the study, taking into account sources of potential bias or imprecision. Discuss both direction and magnitude of any potential bias	20
Interpretation	20	Give a cautious overall interpretation of results considering objectives, limitations, multiplicity of analyses, results from similar studies, and other relevant evidence	21
Generalisability	21	Discuss the generalisability (external validity) of the study results	20
**Other information**			
Funding	22	Give the source of funding and the role of the funders for the present study and, if applicable, for the original study on which the present article is based	22

*Give information separately for exposed and unexposed groups.

Note: An Explanation and Elaboration article discusses each checklist item and gives methodological background and published examples of transparent reporting. The STROBE checklist is best used in conjunction with this article (freely available on the Web sites of PLoS Medicine at http://www.plosmedicine.org/, Annals of Internal Medicine at http://www.annals.org/, and Epidemiology at http://www.epidem.com/). Information on the STROBE Initiative is available at www.strobe-statement.org.

For peer review only

Supply and demand-side factors influencing uptake of malaria testing services in the community: lessons for scale-up from a cluster-randomised, community-based trial in western Kenya

Journal:	*BMJ Open*
Manuscript ID	bmjopen-2022-070482.R1
Article Type:	Original research
Date Submitted by the Author:	16-Mar-2023

Complete List of Authors:	Kirui, Joseph; Academic Model Providing Access to Healthcare, AMPATH<br>Malinga, Josephine; Duke Global Health Institute<br>Sang, Edna; Duke Global Inc, Kenya<br>Ambani, George; Academic Model Providing Access to Healthcare<br>Abel, Lucy; Academic Model Providing Access to Healthcare<br>Nalianya, Erick; Duke Global Inc, Kenya<br>Namae, Jane; Moi University College of Health Sciences<br>Boyce, Matthew; Georgetown University<br>Laktabai, Jeremiah; Academic Model Providing Access to Healthcare; Moi University College of Health Sciences<br>Menya, Diana; Moi University School of Public Health<br>O'Meara, Wendy; Duke Global Health Institute, ; Moi University,

**Primary Subject Heading**:	Global health
Secondary Subject Heading:	Health services research, Infectious diseases
Keywords:	Public health < INFECTIOUS DISEASES, Community child health < PAEDIATRICS, International health services < HEALTH SERVICES ADMINISTRATION & MANAGEMENT

SCHOLARONE™
Manuscripts

I, the Submitting Author has the right to grant and does grant on behalf of all authors of the Work (as defined in the below author licence), an exclusive licence and/or a non-exclusive licence for contributions from authors who are: i) UK Crown employees; ii) where BMJ has agreed a CC-BY licence shall apply, and/or iii) in accordance with the terms applicable for US Federal Government officers or employees acting as part of their official duties; on a worldwide, perpetual, irrevocable, royalty-free basis to BMJ Publishing Group Ltd ("BMJ") its licensees and where the relevant Journal is co-owned by BMJ to the co-owners of the Journal, to publish the Work in this journal and any other BMJ products and to exploit all rights, as set out in our licence.

The Submitting Author accepts and understands that any supply made under these terms is made by BMJ to the Submitting Author unless you are acting as an employee on behalf of your employer or a postgraduate student of an affiliated institution which is paying any applicable article publishing charge ("APC") for Open Access articles. Where the Submitting Author wishes to make the Work available on an Open Access basis (and intends to pay the relevant APC), the terms of reuse of such Open Access shall be governed by a Creative Commons licence – details of these licences and which Creative Commons licence will apply to this Work are set out in our licence referred to above.

Other than as permitted in any relevant BMJ Author's Self Archiving Policies, I confirm this Work has not been accepted for publication elsewhere, is not being considered for publication elsewhere and does not duplicate material already published. I confirm all authors consent to publication of this Work and authorise the granting of this licence.

For peer review only

Supply and demand-side factors influencing uptake of malaria testing services in the community: lessons for scale-up from a cluster-randomised, community-based trial in western Kenya

Authors and affiliations:

Joseph Kipkoech[1*], Josephine Malinga[2], Edna Sang[3], George Ambani[1], Lucy Abel[1], Erick Nalianya[3], Jane Namae[4], Matthew Boyce[5], Jeremiah Laktabai[1,4], Diana Menya[6], Wendy Prudhomme O'Meara[2,6]

[1]Academic Model Providing Access to Healthcare, Eldoret, Kenya

[2]Duke Global Health Institute, Durham, North Carolina, USA.

[3]Duke Global Inc, Kenya.

[4]School of Medicine, Moi University College of Health Sciences, Eldoret Kenya.

[5]Georgetown University, Washington DC, USA.

[6]School of Public Health, Moi University College of Health Sciences, Eldoret, Kenya.

[*]Corresponding author

Keywords: Community-based programs, demand and supply, health interventions, malaria, diagnostic testing, Community Health Workers

List of abbreviations: Artemisinin Combination Therapy (ACT), Artemether Lumefantrince (AL) Community Health Extension Worker(CHEW), Community Health Worker (CHW), Community Unit (CU), Global Positioning System (GPS), Ministry of Health (MOH), Malaria Rapid Diagnostic Test (mRDT), World Health Organization (WHO)

For peer review only - http://bmjopen.bmj.com/site/about/guidelines.xhtml

Corresponding Author: Joseph Kipkoech, Email: josepheddykipkoech@gmail.com

Abstract

Objectives: Maximizing the impact of community-based programs requires understanding how the supply of, and demand for, the intervention interact at the point of delivery.

Design: We present a post-hoc analysis from a large-scale community health worker (CHW) study designed to increase the uptake of malaria diagnostic testing.

Setting: Survey respondents were identified during a household survey and interviewed at home.

Participants: Household members with fever in the last four weeks were interviewed at 12- and 18-months post-implementation. We collected monthly testing data from 244 participating CHWs and conducted semi-structured interviews with a random sample of 70 CHWs.

Primary and secondary outcome measures: The primary outcome measure was diagnostic testing before treatment for a recent fever. The secondary outcomes were receiveing a test from a CHW and tests done per month by each CHW.

Results: 55% (n=948/1738) reported having a malaria diagnostic test for their recent illness of which 38% were tested by a CHW. Being aware of a local CHW (Adj.OR=1.50, 95%CI:1.10-2.04) and belonging to the wealthiest households was associated with higher testing uptake compared to belonging to the least wealthy/poorest households (Adj.OR=1.53, 95%CI:1.14-2.06). Wealthier households were *less* likely to receive their test from a CHW compared to

For peer review only - http://bmjopen.bmj.com/site/about/guidelines.xhtml

poorer households (Adj.OR=0.32, 95%CI:0.17-0.62). Confidence in artemether-lumefantrine(AL) to cure malaria (Adj.OR=**2.75,** 95%CI:1.54-4.92) and perceived accuracy of an mRDT (Adj.OR=2.43**,** 95%CI:1.12-5.27) were positively associated with testing by a CHW. Specific CHW attributes were associated with higher testing rates including formal employment, serving more than 50 households and serving areas with a higher test positivity. On both the supply side and the demand side, confidence in a test performed by a CHW was strongly associated with higher mRDT testing rates.

Conclusion: Scale-up of community-based malaria testing is feasible and effective at increasing access among the poorest households. To maximize the impact of such interventions, it is important to recognize factors that may restrict both delivery and demand for such services.

Strengths and Limitations

1. **This analysis draws on a large multidimensional dataset describing uptake of community-based services and delivery of services in the context of a cluster-randomised implementation trial**
2. **These data include population groups that were successfully reached with the intervention as well as those that may require alternative approaches in order to achieve adequate coverage of diagnostic testing before treatment with antimalarials.**
3. **Triangulating data from the community (intervention recipient) and Community Health Workers (intervention delivery) allowed us to examine strengths and shortcomings from two unique perspectives**

For peer review only

63 **4. As a secondary analysis of trial data, we were limited in the scope of the data available and not able to fully explore the interaction between the CHWs and households nor the complex determinants of health seeking behavior.**

For peer review only

For peer review only - http://bmjopen.bmj.com/site/about/guidelines.xhtml

Background

Despite decades of control efforts, malaria remains a major public health problem. In 2019, an estimated 229 million cases of malaria occurred worldwide. Most malaria cases in 2019 were in the World Health Organization (WHO) African Region (215 million or 94%). In the same year, there were an estimated 409 000 deaths from malaria globally, with Africa again accounting for 94%. Children under 5 years are the most vulnerable group affected by malaria and in 2019, 67% of malaria deaths were in this group (1).

Prompt malaria diagnosis either by microscopy or rapid diagnostic tests (mRDTs) is recommended by WHO for all patients with suspected malaria before they are given treatment (2). Already, 95 countries have adopted the policy of testing all patients with suspected malaria before being treated, and this number is increasing. Diagnostic testing is free of charge in the public sector of almost 90 countries(3). Early and accurate diagnosis is essential both for effective management of the disease and for strong malaria surveillance. Parasite-based diagnostic testing significantly reduces illness and death by enabling health providers to swiftly distinguish between malarial and non-malarial fevers and select the most appropriate treatment(4).

The Kenya National Control Program adopted the Test and Treat policy in (2010) and this has been cascaded to the community level. The mRDTs are recommended for parasitological diagnosis in public health facilities where microscopy is not available and through Community Health Workers in some regions. Despite these efforts, most people in rural Kenya still opt for self-treatment and seek care in the retail health sector(5,6). In our study setting, mRDT are not available in pharmacies and chemists, therefore patients who purchase antimalarials in the retail sector are not diagnosed before treatment. Studies have shown that targeting of antimalarials to true malaria cases in the retail sector is very low; as few as 20% of people purchasing

For peer review only

antimalarials are suffering from malaria (7,8) and as high as 70% of people with malaria do not purchase an ACT(9).

The deployment of mRDTs through trained community health workers could improve access to diagnosis for populations with limited access to health facilities. We evaluated an intervention that targeted individuals who seek care in the retail sector by offering free community-based malaria diagnostic testing through CHWs. Following testing, those with a positive test result received a voucher to obtain first-line antimalarials at a nearby pharmacy at a heavily subsidized price. The intervention demonstrated a significant improvement in testing before treatment (6).

Ideally, there should be adequate demand for the commodity or opportunity, and supply should be able to meet demand. In our previous study, only 55 percent of those with recent illness reported having a malaria test before taking drugs and 21% still purchased antimalarials over-the-counter without a test (6), representing substantial scope for further improving the reach and impact of this intervention.

Here we report a secondary analysis of data collected during a large cluster randomised trial of community-based diagnostic testing in western Kenya. We sought to understand what factors might increase or decrease demand for testing from the community, compared to factors that may have limited supply of testing by CHWs. A comparison of constraints on the demand side and supply side could help understand the conditions under which the intervention operated well or was hindered by a mismatch in supply and demand. The results will be instrumental for improving the design of community-based interventions delivered at scale.

Methods

Description of the main trial

For peer review only - http://bmjopen.bmj.com/site/about/guidelines.xhtml

This is a post-hoc analysis of data collected during a large community based cluster randomised trial. Results of the study have been reported elsewhere (6). The purpose of parent trial was to encourage clients to receive testing for malaria before seeking treatment. This was accomplished by training and equipping community health workers (CHWs) to perform rapid-diagnostic tests (mRDTs) for malaria and to provide clients who test positive for malaria with a voucher that allows them access to subsidized high-quality antimalarial drugs through participating pharmacies. (6) The trial was conducted in 32 community clusters in western Kenya (population approximately 160,000). Eligible clusters had retail outlets selling AL and existing community health worker (CHW) programs and were randomly assigned 1:1 to control and intervention arms. In intervention areas (comprised of 16 community units), CHWs were available in their villages to perform malaria rapid diagnostic tests (mRDTs) on demand for any individual >1 year of age experiencing a malaria-like illness. The mRDT-positive individuals received a voucher for a discount on a quality-assured AL, redeemable at a participating retail medicine outlet. In control cluster (composed of 16 community units), CHWs offered a standard package of health education, prevention, and referral services. We conducted 4 population-based surveys—at baseline, 6 months, 12 months, and 18 months—of a random sample of households with fever in the last 4 weeks to evaluate predefined, individual-level trial outcomes.

In Kenya, CHWs work on a volunteer basis and there are no formal education requirements.The ability to read and write are criteria for selection by the MOH. The role of CHWs as defined by the MOH is to promote good health through providing health education, basic treatments (such as first-aid), and referrals to health care facilities. The CHWs report to Community Health Extension Workers (CHEWs) who are employees of MOH and are trained community health nurses

For peer review only - http://bmjopen.bmj.com/site/about/guidelines.xhtml

mandated to supervise the activities of CHWs in the community units. In intervention areas, existing CHWs were trained to carry out the intervention.

CareStart HRP2-based malaria mRDTs were used for testing. CareStart HRP2 detects *Plasmodium falciparum* which is the main species of parasite responsible for the majority of malaria cases in Kenya (10). In addition to malaria mRDT, the study team provided CHWs with the necessary supplies to conduct the mRDT testing. CHWs were free to perform tests in the location(s) of their choice (e.g., in their own homes, at clients' homes, etc.), as long as the location allowed for proper and sufficiently private performance of the procedure.

The CHWs tested febrile patients at community level and issued AL vouchers to patients with positive results. This CHW model differs from other settings implementing community case management of malaria where the CHW is permitted to both perform the mRDT and provide antimalarials directly to the client. The voucher redemption period was three days where by after that the Medicine shops were not allowed to collect the voucher. Patients with positive malaria results bought AL from medicine shops by redeeming their vouchers and leaving the voucher at the shop. The study team collected the redeemed vouchers from the medicine shops on a bi-weekly basis and reimbursed the difference in costs.

Community surveys

A community-based survey was conducted to collect individual-level study outcomes based on a population-based survey sampling strategy. This was a repeated cross-sectional household survey at 4 time points: baseline (pre-intervention), 6 months, 12 months, and 18 months post baseline. The survey was conducted in all the 32 community units, 16 Intervention and 16 control.

For peer review only

Households were randomly selected by systematic random sampling and one person with fever in the last four weeks was interviewed in any household. If more than one eligible fever was found in a household, one participant would be selected based on alphabetical order of the given names. Details of community surveys sample size calculation are described elsewhere(11) . Briefly, the target sample size of eligible individuals with a fever in the last 4 weeks was 640 per arm (i.e., 40 per CU) at each time point, which—assuming 22.2% of households would have an eligible member—would require contacting 5,766 households. The starting point (index house) and sampling interval were different in each survey round in order to minimize repeat surveys across multiple rounds. We restrict our analysis here to the 12 and 18 month surveys in order to evaluate the effects of interest in the time-period when the intervention effect could be measured (6).

Basic demographic information about the participants and the households were collected. Socioeconomic scores were calculated from an asset index and then grouped into quintiles as described previously. Euclidean distance from a household to the nearest health facility was calculated from GPS coordinates. In the final survey, participants beliefs about their illness, malaria risk, diagnostic testing, and treatment were elicited by asking them about the severity of their illness, their confidence in positive test result and a negative test result, and how certain they were that they would recover from malaria after treatment with AL. "Perceived prevalence" was defined as the proportion of fevers that the respondent believed would be due to malaria among 10 people with fever from their village. High perceived prevalence was assigned to those who responded 8-10 out of 10, medium prevalence to those who responded 4-7 out of 10 and low to those who responded 0-3 out of 10. The survey tool can be found in supplemental materials.

Midpoint CHW Survey

For peer review only - http://bmjopen.bmj.com/site/about/guidelines.xhtml

As part of the process evaluation, at the midpoint of this study, interviews were conducted with a random sample of 70 CHWs who were trained to conduct mRDTs (intervention CUs). The primary objective of these interviews were to determine the CHWs' satisfaction with their role in the malaria project and the feasibility of their continued involvement. Secondary objectives included eliciting the main challenges the CHWs have faced in implementing the study, determining unanticipated burdens on the CHWs' other activities (including their responsibilities as CHWs outside of the study as well as their non-CHW responsibilities) and identifying changes in the types of services they provide and the recipients of their services(12).

Likert-scale questions were also asked, in which the interviewers read a statement aloud and the CHWs were asked to respond how much they agreed with the statement on a five-point scale ("strongly agree", "somewhat agree," "neutral," "somewhat disagree," "strongly disagree"). For this analysis, several variables were derived from the survey data. The client score was constructed as a composite score based on four likert scale questions – whether CHWs agreed that; clients trust the results of a positive mRDT by the CHW, they trust a negative mRDT, they follow the CHWs advice if the test was positive, they follow advice if the test was negative. A response of 'strongly agree'=2, somewhat agree='1' and neutral/somewhat disagree/strongly, disagree=0. If the sum of these four responses was 6 or greater, the score was 'high'. If less than 6, the score was 'low'. The survey tool can be found in supplemental materials.

These same CHWs also performed an mRDT under observation of the study supervision team and were scored on each step using a 20-step checklist that classified steps as relating to safety, accuracy, and treatment. The checklist was developed by the study team based on previous research studies to guide training and support supervision. Those who completed at least 17 of 20 steps correctly had a 'high' score for mRDT performance and <17 had a 'low' score.

For peer review only - http://bmjopen.bmj.com/site/about/guidelines.xhtml

200 **Demand and Supply**

201 To investigate factors that might have influenced the success of the intervention, the analysis was divided into demand and supply factors. On the demand side, the analysis investigates factors that might influence the community member's outcome of having a test, first from any source and then from the CHW. Only respondents with complete data on the variables of interest were included. On the supply side, the analysis investigates the factors associated with the testing output (supply) at the CHW level, measured as the monthly number of tests conducted by each CHW. These factors include; socio-demographic characteristics of the CHW, number of households under the CHWs care, years of experience of the CHW, the number of other activities the CHW engages in under their role as a CHW (under the assumption that an increasing number of activites may compete for their time, the CHW provided a list of activities and these were summed together), the perception of the intervention by the CHW, the skill of the CHW in conducting mRDTs and the overall prevalence of malaria in their community.

213 **Outcome Measures**

214 This analysis looks at the clusters in the intervention arms. The primary outcome is the uptake of testing, defined as the proportion of fevers in the previous 4 weeks that receive a diagnostic test from any source. The second primary outcome is the uptake of testing from a community health worker among those with any test. We present descriptive results for individuals getting tested from any source and from a CHW across the intervention clusters.

219 The secondary outcome was to investigate the testing volume and the factors influencing this. This was measured by the mean monthly tests for each CHW. Monthly number of tests per CHW was available from program data collected at monthly supervision visits.

For peer review only - http://bmjopen.bmj.com/site/about/guidelines.xhtml

Statistical Analysis

A mixed-effects logistic regression analysis was used to evaluate the primary outcome measures on the uptake of testing and investigate risk factors that might have contributed to the outcomes of the intervention reported in (6). The hierarchical nature of the data informed the adoption of multi-level modelling, since we include both individual-, and CHW- level risk factors in the analysis and account for clustering at the CU-level. The outcomes were evaluated only for the CUs in the intervention arm and summaries provided.

For the primary outcome measures to evaluate the uptake of testing, two logistic models were generated; the first model included all individuals who reported fever in the last 4 weeks. The second model includes a subset of those in the first model, specifically those with a fever and were tested at any source. For both models, we accounted for clustering at the CU level, and then included potential individual-level covariates like respondent's age, gender, level of educational, occupation, socio-economic status, geographic proximity to health care provider, the time they waited after falling ill before they sought a test, the level of knowledge and opinions about malaria and malaria diagnosis.

The logistic regression model is as shown below:

$$log\left(\frac{p_{ij}}{1-p_{ij}}\right) = \beta_0 + \beta_1 X_{1j} + \ldots + \beta_m X_{mj} + u_j$$

where p_{ij} is the probability of the outcome for each individual i in cluster j, β_0 is the model intercept, β_{1-m} are the coefficients for each potential risk factor X_{1-m}, and u_j represents the random effects for cluster j. The structures for both models are the same, however, for model 1, any test=1 and no test=0 and for or model 2, tested at CHW=1 and tested elsewhere=0.

For peer review only - http://bmjopen.bmj.com/site/about/guidelines.xhtml

Fixed effects are measured using an Odds Ratio (OR) and included the 95% confidence intervals. Independent categorical variables were screened for multicollinearity with a Cramér's V statistic. In case two variables showed signs of correlation (V > 0.7), the variable least associated with the response variable was omitted (13).

For the secondary outcome measure, a linear regression model adjusted for clustering at the CU level was used. This model investigates the outcome of mean RDTs performed each month by the specific CHW and includes potential CHW-level covariates like CHW age, marital status, education level, number of households responsible, previous training on malaria case management or mRDTs, years of experience as a CHW, and formal employment status.

The linear regression model is as shown below:

$$Y_{ij} = \beta_0 + \beta_1 X_{1j} + \dots + \beta_m X_{mj} + u_j + \varepsilon_{ij}$$

where Y_{ij} is the outcome for each individual i in cluster j, β_0 is the model intercept, β_{1-m} are the co-efficients for each potential risk factor X_{1-m}, u_j represents the random effects for cluster j, and ε_{ij} is the error term.

All statistical analysis was done in STATA version 15 (StataCorp).

Patient and Public Involvement

No patients involved

For peer review only - http://bmjopen.bmj.com/site/about/guidelines.xhtml

260 **Results**

261 A total of 292 CHWs in 16 Intervention CUs were trained to perform mRDTs and 36 retail shops
262 were enrolled to redeem vouchers. The intervention was launched on July 21, August 17, and
263 September 22, 2015 in Bokoli, Kiminini, and Ndivisi sub-counties western Kenya, respectively
264 and continued until May 5, 2017.

265 *Demand side factors for use of malaria diagnostic testing*

266 In total, 3719 households were approached, 1877 had an eligible fever in the last four weeks, 56
267 did not know if there was an eligible fever, 0 refused and 1738 completed the survey with no
268 missing answers (92.6%). The community-based demand analysis relies on 1738 complete
269 surveys from participants in randomly selected households in intervention clusters with an acute
270 illness in the last four weeks (**Table 1**). Community members were interviewed at 12 or 18 months
271 (47.2% and 52.8% respectively) following the initiation of the intervention. 55% (n= 948) of
272 participants reported having a malaria test for their recent illness while 38.4% (n=364/948) of
273 those tested reported having had the test at CHW. Among all surveyed participants, 41% (n= 710)
274 were male and 59% (n= 1028) were female. 21.2% (n= 368) of the recent fevers were aged <5
275 years, 43.3% (n= 752) were 5 -17 years while 36% (n= 618) were 18> years. Only 21.4% (n=
276 371) of the participants had completed their secondary education while 45% (n= 778) had
277 completed their primary education. On average, the distance to the nearest public health facility
278 was approximately 2km while the nearest drug shop was approximately 1.3km away.

279 **Table 1: Characteristics of community survey participants who reported malaria test in 12**
280 **months and 18 months.**

Participant characteristics	(n)	%

For peer review only - http://bmjopen.bmj.com/site/about/guidelines.xhtml

(N=1738)		
Tested at any location	948	54.6
Tested at CHW	364	20.0
Patient gender		
Male	710	40.9
Female	1028	59.1
Patient age		
Under 5	368	21.1
5-17 y	752	43.3
18+y	618	35.6
Education level of respondent		
Not completed primary	589	33.9
Completed primary	778	44.8
Completed secondary	371	21.3
Survey timepoint		
12 months	821	47.2
18 months	917	52.8
Household characteristics (N=1738)	Mean	Standard deviation
Distance to the nearest public health facility, km	2.1	1.2
Distance to nearest drug shop, km	1.3	0.7

Demand-side factors that were associated with uptake of malaria testing for their most recent illness are examined (**Table 2**). Respondents who delayed seeking care beyond the first 24 hours had higher odds of receiving a test. Being aware of your local CHW increased the odds of having a test from any source, facility or CHW (Adj. OR=1.50, 95% CI: 1.10-2.04). Respondents from the wealthiest households were 53% more likely to have a test (Adj. OR=1.53 95%CI: 1.14-2.06). Being an adult at least 18 years old was negatively associated to taking a test (Adj. OR=0.59, 95% CI:0.43-0.82). Distance to a health facility greater than 2km (Adj. OR=0.86, 95% CI:0.67-0.11) and use of ITN (Adj. OR=1.27, 95%CI:0.93-1.74) were not significantly associated with testing uptake.

For peer review only - http://bmjopen.bmj.com/site/about/guidelines.xhtml

291 **Table 2. Association between participant characteristics and malaria diagnostic testing.**
292 **Adjusted and unadjusted odds ratios for receiving a malaria diagnostic test, either**
293 **microscopy or mRDT, from any source for the most recent febrile illness.**

	Univariate analysis		Multivariate analysis	
Participant characteristics (N=1738)	Unadjusted Odds Ratio (95%CI)	P-value	Adjusted Odds Ratio (95%CI)	P-value
Time to seek care (*Reference=same day*)				
Next day	**1.21[1.05,1.42]**	**0.01**	**1.23[1.06,1.43]**	**0.01**
After two days	1.01[0.78,1.32]	0.94	1.13[0.86,1.48]	0.38
More than two days	0.94[0.73,1.22]	0.66	1.12[0.87,1.44]	0.37
Use an ITN *(Reference=does not use an ITN)*	1.37[0.99,1.92]	0.06	1.27[0.93,1.74]	0.13
Distance to public health facility >2km (*Reference = <2km*)	0.83[0.64,1.06]	0.14	0.86[0.67,1.11]	0.26
Aware of a CHW in their village *(Reference=is not aware of a CHW in their village)*	**1.43[1.01,2.05]**	**0.04**	**1.50[1.10,2.04]**	**0.01**
Patient age (*Reference=1-4y*)			-	-
5 to 17y	0.76[0.54,1.08]	0.12	0.79[0.56,1.12]	0.18
18+y	**0.59[0.43,0.81]**	**<0.01**	**0.59[0.43,0.82]**	**<0.01**
Patient is male (*Reference = female*)	1.02[0.86,1.23]	0.78	0.94[0.80,1.10]	0.43
Respondent education level (*Reference=none*)			-	-
Completed primary	1.12[0.93,1.36]	0.23	1.08[0.87,1.33]	0.48
Completed secondary	**1.41[1.04,1.90]**	**0.03**	1.25[0.93,1.70]	0.14
Wealth quintile (*Reference=0-20th*)			-	-
20 - 40th	1.04[0.68,1.62]	0.83	1.02[0.65,1.59]	0.94
40 - 60th	1.14[0.86,1.50]	0.36	1.08[0.82,1.43]	0.59
60 - 80th	1.10[0.79,1.53]	0.56	1.03[0.73,1.46]	0.86
>80th	**1.59[1.15,2.21]**	**<0.01**	**1.53[1.14,2.06]**	**<0.01**

294 * This table refers to those who reported getting a test from any source

295

For peer review only - http://bmjopen.bmj.com/site/about/guidelines.xhtml

296

297 The analysis further focused on those who were tested for malaria by a CHW **(Table 3).** Out of
298 948 participants who reported having a test, 38.4% reported having taken a test from a CHW
299 (364/948). It is noted that wealthier participants prefer being tested at the health facility (Adj. OR
300 for testing at a CHW=0.32, 95%CI:0.17-0.60), and those with education above secondary
301 school have lower odds of taking a test at the CHW (Adj. OR=0.68, 95%CI:0.44-1.07). Delaying
302 testing beyond two days also reduced the odds of testing by a CHW. On the other hand, school-
303 aged children between 5-17 years were more than twice as likely to be tested by a CHW
304 compared to those below 5 years old (Adj. OR=2.39, 95%CI: 1.43-4.01).

305 **Table 3: Association between patient characteristics and having an mRDT from a CHW**
306 **before taking any treatment.**

	Univariate analysis		Multivariate analysis	
Participant Characteristics of those who took a test from CHW (n=948)	Unadjusted Odds Ratio (95%CI)	P-value	Adjusted Odds Ratio (95%CI)	P-value
Time to malaria test (*Reference=same day*)			-	-
Next day	0.96[0.71,1.31]	0.80	0.96[0.69,1.33]	0.81
After two days	0.73[0.44,1.21]	0.22	0.68[0.42,1.10]	0.12
More than two days	0.81[0.49,1.34]	0.41	0.87[0.50,1.52]	0.62
Use an ITN *(Reference=does not use an ITN)*	**0.57[0.41,0.78]**	**<0.01**	0.77[0.55,1.06]	0.11
Distance to public health facility>2km (*Reference = <2km*)	1.32[0.80,2.20]	0.28	1.29[0.82,2.03]	0.27
Patient age (*Reference=1-4y*)			-	-
5 to 17y	**2.43[1.49,3.95]**	**<0.01**	**2.39[1.43,4.01]**	**<0.01**
18+y	0.98[0.67,1.43]	0.91	1.09[0.72,1.66]	0.68
Patient is male (*Reference = female*)	1.23[0.98,1.54]	0.07	1.02[0.82,1.26]	0.85

For peer review only - http://bmjopen.bmj.com/site/about/guidelines.xhtml

Respondent education level (*Reference=none*)			-	-
Completed primary	0.86[0.69,1.09]	0.22	0.96[0.75,1.22]	0.74
Completed secondary	**0.45[0.29,0.68]**	**<0.01**	**0.68[0.44,1.07]**	**0.10**
Wealth quintile (*Reference=0-20th*)			-	-
20 - 40th	1.13[0.65,1.94]	0.671	1.11[0.63,1.96]	0.72
40 - 60th	0.83[0.63,1.09]	0.175	0.79[0.58,1.05]	0.11
60 - 80th	0.67[0.47,0.96]	0.028	0.66[0.45,0.97]	0.03
>80th	**0.26[0.13,0.53]**	**<0.001**	**0.32[0.17,0.62]**	**<0.01**

* This table refers to those who reported getting a test from a CHW

To understand how individual perceptions about malaria testing and treatment, malaria risk, and illness severity affected uptake of testing or testing at a CHW, the analysis focused on the final survey at 18 months survey **(Table 4)**. After adjusting for demographic characteristics, patient perceptions were differentially related to testing overall and testing specifically with a CHW. Both confidence in AL treatment (Adj. OR=2.75, 95% CI: .1.54-4.92) and confidence in the accuracy of an mRDT performed by a CHW (Adj. OR=2.43, 95% CI: 1.12-5.27) were strongly positively associated with testing at a CHW. Those who reported their illness as severe were more likely to be tested (Adj. OR=2.37, 95% CI: 1.58-3.58) but had a lower odd of testing at a CHW (Adj. OR=0.44, 95% CI: 0.22-0.87). Confidence in an mRDT result, either positive or negative was not significantly associated with receiving a test for a recent illness, but those who reported a high confidence in a negative mRDT result had substantially higher odds of testing with a CHW than in a facility, although this did not reach statistical significance at the 95% level (Adj OR=1.58, 95%CI: 0.89-2.83). Finally, those who reported a moderate perceived prevalence in their village (ie 4-7 fevers out of 10 would be due to malaria) or high perceived prevalence (ie 8-10 out of 10 fevers would be due to malaria) had increasingly higher odds of receiving a test from a CHW

For peer review only - http://bmjopen.bmj.com/site/about/guidelines.xhtml

(medium prevalence Adj. OR=2.52, 95%CI: 1.26-5.06, high prevalence Adj. OR=2.70, 95% CI: 1.17-6.22).

Table 4: Relationship between participant beliefs and perceptions and uptake of testing from any source or from a CHW. Models are adjusted for patient age, gender, household wealth quintile and the education level of the respondent in addition to the variables in the table.

	Univariate analysis		Multivariate analysis	
**Had a malaria test (N=703)**	Unadjusted Odds Ratio (95%CI)	P-value	Adjusted Odds Ratio (95%CI)	P-value
Confidence in a positive mRDT result	1.02[0.67,1.54]	0.93	0.87[0.53,1.41]	0.57
Confidence in a negative mRDT result	1.34[0.87,2.03]	0.19	1.24[0.72,2.14]	0.43
Confidence in AL treatment	1.27[0.94,1.71]	0.12	1.06[0.81,1.40]	0.63
Trust an mRDT by a CHW as much as one at a facility	0.85[0.55,1.32]	0.48	**0.66[0.44,0.97]**	**0.03**
Illness is severe	**2.36[1.64,3.39]**	**<0.001**	**2.37[1.58,3.58]**	**<0.001**
Prevalence (*Reference= low*)			-	-
Medium	1.51[0.89,2.57]	0.13	1.48[0.85,2.58]	0.16
High	1.12[0.70,1.80]	0.64	1.05[0.63,1.77]	0.85
	Univariate analysis		Multivariate analysis	
**Had a test with a CHW (N=428)**	Unadjusted Odds Ratio (95%CI)	P-value	Adjusted Odds Ratio (95%CI)	P-value
Confidence in a positive mRDT result	1.45[0.89,2.34]	0.13	1.04[0.64,1.71]	0.86
Confidence in a negative mRDT result	1.61[0.99,2.64]	0.06	1.58[0.89,2.83]	0.12
Confidence in AL treatment	**2.67[1.52,4.70]**	**<0.01**	**2.75[1.54,4.92]**	**<0.01**
Trust an mRDT by a CHW as much as one at a facility	**2.91[1.29,6.58]**	**0.01**	**2.43[1.12,5.27]**	**0.03**
Illness is severe	0.60[0.35,1.03]	0.06	**0.44[0.22,0.87]**	**0.01**
Prevalence (*Reference= low*)			-	-
Medium	**2.87[1.48,5.56]**	**<0.01**	**2.52[1.26,5.06]**	**0.01**
High	**2.85[1.31,6.23]**	**0.01**	**2.70[1.17,6.22]**	**0.02**
Confidence in a positive mRDT result	1.45[0.89,2.34]	0.13	1.04[0.64,1.71]	0.86

For peer review only

Diagnostic testing by CHWs

In total, 32,404 mRDTs were conducted by the CHWs over the intervention period and 33.7% (n= 10,870) percent were positive. All mRDT interpretations were counterchecked by the study team. Those with a positive test received a voucher for a discounted quality-assured AL. These vouchers were redeemed at a participating outlet by 93.9% of voucher recipients. There was no instances of stock out of mRDTs among our CHWs. To explored factors that may be associated with the CHWs ability to conduct mRDTs, we examined the association between testing volume, defined as the mean number of mRDTs conducted per month, and the different CHW demographic characteristics (**Table 5**). Of the 244 trained CHWs, 147 (60.3%) were above 40 years of age, 72 (29.5%) were male. There were 107 (43.8%) CHWs who had not completed secondary education.

Table 5: Association between mean number of tests performed per month and CHW characteristics

Mean number of tests per month	4.60 (SD=3.07)	**Univariate Analysis**		**Multivariate Analysis**	
		**N = 244**		**N = 244**	
CHW characteristics	n (sample proportion)	Coeff (95%CI)	P-value	Coeff (95%CI)	P-value
Male	72 (0.295)	-0.30 [-1.28,0.68]	0.52	-0.28 [-1.24,0.68]	0.54
Age<br><40 years *(Reference)*	97 (0.397)	-	-	-	-
> = 40 years	147 (0.603)	-0.21 [-1.06,0.63]	0.60	-0.42 [-1.12,0.28]	0.23
Married	202 (0.828)	0.37 [-0.57,1.30]	0.42	0.22 [-0.92,1.35]	0.69

For peer review only

For peer review only - http://bmjopen.bmj.com/site/about/guidelines.xhtml

(Reference = unmarried/divorced)					
Education<br>Below secondary (*Reference*)	107 (0.438)	-	-	-	-
Completed Secondary	137 (0.562)	0.45 [-0.33,1.23]	0.24	0.23 [-0.40,0.87]	0.44
Formal Employment *(Reference = no formal employment)*	33 (0.135)	**1.13 [-0.01,2.28]**	**0.05**	**1.37 [0.05,2.70]**	**0.04**
Previously trained in mRDT	46 (0.188)	-0.46 [-1.64,0.72]	0.42	**-1.40 [-2.44, -0.37]**	**0.01**
Households[1]<br><50 (*Reference*)	134 (0.549)	-	-	-	-
50-100	66 (0.271)	**2.14[0.63,3.64]**	**<0.01**	**1.73[0.70,2.74]**	**<0.01**
>100	44 (0.180)	1.18[-0.40,2.76]	0.13	**1.49[0.74,2.24]**	**<0.01**
Cluster-level test positivity[2]<br>Low (≦25%; *Reference)*	141 (0.588)	-	-	**-**	**-**
High (>25%)	103 (0.422)	**2.54[0.97,4.10]**	**<0.01**	**2.14[1.05,3.22]**	**<0.01**

[1] Number of households for which the CHW reported being responsible

[2] Percentage of mRDTs conducted by the CHWs in that cluster which turned positive over the study period.

In a univariate and multivariate analysis, formal employment, previous mRDT training, number of households and mRDT-positivity rate at the cluster-level were associated with volume of tests done. Sex, age, being married and education level were not significantly associated with testing volume. CHWs who were formally employed in some other capacity alongside their CHW voluntary work performed on average 1.37 more tests per month (Adj coefficient=1.37, 95% CI: CI: 0.05-2.70) but those who reported being trained previously on mRDTs performed 1.4 fewer tests per month (Adj coefficient=1.4, 95%CI: -2.44,-0.37). CHWs serving areas with a high proportion of positive tests (proportion mRDT +ve >25%) tested on average 2 more clients per month than those in lower prevalence areas (Adj coefficient= 2.14, 95%CI: 0.63-3.64). CHWs who were responsible for at least 50 households tested more clients (Adj coefficient=1.73, 95%CI:

For peer review only

For peer review only - http://bmjopen.bmj.com/site/about/guidelines.xhtml

0.70,2.74), although this did not increase further with when the number of households exceeded 100 (Adj coefficient = 1.49, 95%CI: 0.74-2.24).

A univariate and multivariate analyses to test the association between testing volume and CHW perceptions about their role at the midpoint survey (**Table 6**). High client score was significantly associated with higher testing volume performed by CHWs who perceived that their clients trusted their tests (Adj. coefficient=1.37, 95%CI: 0.11-2.62). The CHWs competency at conducting all 20 steps of the mRDT process (mRDT score), the number of different activities to which CHWs were committed, whether they named mRDT testing as their most important activity, or whether they cited extrinsic motivation (money, airtime, or other) as important reasons for continuing their work were all not significantly associated with average tests performed per month.

Table 6: Association between testing volume and CHW perceptions about their role at the midpoint survey. Multivariate odds ratios are adjusted for age, gender, education level, formal employment. The analysis includes a random sample of CHWs selected for midpoint evaluation.

	n (sample proportion)	**Univariate Analysis**		**Multivariate Analysis[1]**	
CHW characteristics	N=70	Coeff (95%CI)	P-value	Coeff (95%CI)	P-value
mRDT Score[2] (*Reference=Low*)	-	-	-	-	-
High	54 (0.771)	0.19[-1.25,1.64]	0.78	0.11[-1.34,1.57]	0.87
Number of different activities last month (*Reference=<=3*)	-	-	-	-	-
> 3 activities	19 (0.271)	0.35[-1.56,2.26]	0.70	-0.45[-2.04,1.15]	0.56
Most important activity is malaria testing	48 (0.686)	0.88[-0.25,2.01]	0.12	0.07[-1.16,1.32]	0.90
Cited extrinsic motivators as important[3]	28 (0.400)	0.33[-1.52,2.17]	0.71	0.67[-0.92,2.25]	0.38
Client Score[4] (*Reference= Low*)	-	-	-	-	-
High	48 (0.686)	**1.46[0.10,2.83]**	**0.04**	**1.37[0.11,2.62]**	**0.04**
Years of experience as a CHW (*Reference = <=5y*)	-	-	-	-	-

For peer review only - http://bmjopen.bmj.com/site/about/guidelines.xhtml

>5 years	38 (0.543)	-0.35[-2.68,1.98]	0.75	-0.51[-2.56,1.55]	0.61

[1] Multivariable models are also adjusted for CHW demographic factors (age, gender, education, employment, and cluster-level prevalence)
[2]mRDT score - CHW scored > 17 correct steps on a 20-step checklist for mRDT preparation and interpretation conducted 6 months after training
[3] CHW cited money, non-monetary incentives or desire for recognition as important motivators for their role as a CHW
[4] Client score was derived from four questions where the CHW reported how confident clients were in the testing they provided.

For peer

Discussion

This study adds to the evidence that it is feasible for CHWs to offer malaria testing using mRDTs in the community. With the use of CHWs, large-scale malaria testing intervention can reach the underserved population who cannot afford to visit the health facilities for care and those who seek care in the retail sector. The existence of CHWs with the ability to test for malaria using mRDT provides a great opportunity, however, various factors may affect the demand and supply of testing using mRDT by CHWs.

Here we examine the outcomes of a community-based malaria testing to understand how to improve intervention design and maximize the impact of such programs. The analysis was organized around the target group (community members) and their uptake or demand for the diagnostic testing intervention, and the supply of testing by the CHWs responsible for delivering the intervention. We were able to separate the determinants of uptake of malaria diagnostic testing generally from the determinants of uptake of malaria rapid diagnostic test performed by a

For peer review only - http://bmjopen.bmj.com/site/about/guidelines.xhtml

CHW. These contrasting but complementary analyses helped us identify bottlenecks to intervention coverage at the community level.

Analysis of community survey data revealed that increased demand for malaria diagnostic testing before treating a fever is positively associated to education level and wealth, but lower among adults, findings which are consistent with other published studies (14–17). We also find that being aware of the local CHW increased demand for testing. This is similar to other studies that have evaluated the contribution of CHWs to population health-seeking behavior(16,18). In the context of our program, this is especially important because the testing was initiated by the community member seeking out a CHW rather than a door-to-door campaign initiated by the CHW. Malaria testing by a CHW provides an opportunity for reducing health disparities by ensuring the poor and underserved population get access to testing and are able to make an informed choice on how to manage their illness. Our results show that community members with a lower socioeconomic score were more likely to receive a test from a CHW. Malaria testing by the CHW was free and this finding therefore aligns well with the intention of the intervention. Testing by a CHW was also more common among those with high confidence in the reliability of a test done by a CHW and also among those with high confidence in artemether-lumefantrine, the treatment offered through the CHW testing program. Taken together, these results suggest that the community awareness of and perception of the CHW is key in improving demand. In contrast, although testing was high among those who considered their illness to be very severe, these individuals were less likely to be tested by a CHW. This result is appropriate and consistent with the goals of the program which included immediate referral of more severe cases.

From the perspective of CHWs delivering malaria testing, a strong association was not observed between demographic characteristics and testing volume, but we did note that CHWs with

For peer review only - http://bmjopen.bmj.com/site/about/guidelines.xhtml

concurrent formal employment tested more patients on average than those without which could indicate greater visibility or mobility in the community. It was also observed that the monthly testing volume increased when the number of households under a CHWs purview exceeded 50, but did not increase further when households exceeded 100, suggesting a threshold effect whereby more households created more demand for testing but the ability to meet the demand is saturated after some threshold beyond 100 households. Finally, we report that testing rates were higher in clusters with high mRDT-positivity rates, possible reflecting a greater awareness of malarial illness or higher demand for malaria services in areas with higher malaria burden. This could also reflect higher confidence in a test among CHWs or communities members who have experience with some tests turning positive as has been seen in the healthcare setting (19).

A deeper analysis of CHW perception of the intervention was undertaken in a smaller random sample of participating CHWs. We show that testing volume is not affected by competing CHW-related priorities, but is associated with how they perceive their clients' confidence in testing and the likelihood of the client accepting the mRDT result and advice. This aligns well with the picture from the demand-side where we observe that confidence in an mRDT performed by a CHW is strongly associated to testing with a CHW.

This study had several limitations. First, we are not able to estimate the effect of free testing on the uptake of testing. Considering the study utilized diagnosis dependent subsidies to test the rational use of AL, a similar study that deploys free testing in the absence of a conditional voucher would be required to resolve this. Similarly, it could be important to compare the effect of an intervention that delivered both the test and the treatment through the CHW rather than using a voucher redeemable in the private sector. Second, although the survey data has provided valuable insight on understanding drivers to demand and supply of malaria testing, seeking

For peer review only - http://bmjopen.bmj.com/site/about/guidelines.xhtml

provider (CHW) and patient perspectives could provide a richer understanding of the facilitating factors and barriers to scaling up such an intervention, particularly the ways in which preferences and changes in behavior may influence uptake of the intervention. Finally, though our survey design used repeated cross-sectional sampling, it is possible that the same household and/or febrile individual was surveyed at multiple survey time points and any resulting association was not explicitly modeled in our regression analysis.

Conclusion

In summary, scale up of health interventions through CHWs is feasible and effective at reaching the poorest households. However, in order to maximize the impact of community-based interventions, it is important to recognize factors that may restrict both delivery and demand for such services. It is critical to not only create awareness of CHW services but foster trust in both the CHWs ability to deliver services and the health intervention itself. Engendering confidence in the CHWs proficiency is likely to have a synergistic effect on both demand for services and commitment to delivering services.

Declarations

Ethics approval and consent to participate: Ethical approval was granted by Moi University Institutional Research and Ethics Committee (*Formal Approval No. 0001403*) and Duke University Institutional Review Board (*Pro00063384*). Written informed consent was obtained from participants during household interviews and from CHWs prior to collecting midpoint evaluation data. All study methods were carried out in accordance with ethical guidelines and regulations set out by both Moi University and Duke Ethical Review Boards.

For peer review only

Consent to publish: Not applicable

Availability of data and materials: The datasets used and/or analysed during the current study are available from the corresponding author on reasonable request.

Competing interests: The authors declare that they have no competing interests

Funding: This study was supported from the National Institute of Allergy and Infectious Diseases of the National Institutes of Health (US) (grant number R01AI110478). The funders had no role in study design, data collection and analysis, decision to publish, or preparation of this manuscript. The content is solely the responsibility of the authors and does not necessarily represent the official views of the National Institute of Allergy and Infectious Diseases or the National Institutes of Health.

Authors' contributions:

Joseph Kipkoech – Conceptualization, writing original draft, revision and editing final draft

Josephine Malinga – Data curation, writing original draft, revision and editing final draft

Edna Sang - writing original draft, writing original draft, revision and editing final draft

George Ambani – Data collection, writing original draft, revision and editing final draft

Lucy Abel - Data collection, writing original draft, revision and editing final draft

Erick Nalianya - Data collection, writing original draft, revision and editing final draft

Jane Namae - Data collection, writing original draft, revision and editing final draft

For peer review only - http://bmjopen.bmj.com/site/about/guidelines.xhtml

For peer review only

Matthew Boyce - Review and editing final draft

Jeremiah Laktabai – Conceptualization, review and editing final draft

Diana Menya - Conceptualization, review and editing final draft

Wendy Prudhomme-O'Meara – Conceptualization, funding acquisition, writing original draft, revision and editing final draft

Acknowledgements: This study could not have been carried out without the exceptional attention to detail and commitment of our field team that include: I. Khaoya, L. Marango, E. Mukeli, L. Nukewa, E. Wamalwa, and A. Wekesa. We are grateful to the study participants and the community health workers as well as the local community leadership that supported this work. We would also like to acknowledge the partnership and support of our Health Management Team colleagues in each subcounty and in particular V. Kiplagat, A. Kyalo, S. Malenya, and P. Musita.

Supplemental material

Supplemental file 1: Community survey tool used to collect information about recent fevers among participating households

Supplemental file 2: Survey tool used at the midpoint CHW evaluation.

For peer review only

For peer review only - http://bmjopen.bmj.com/site/about/guidelines.xhtml

References

1. World Health Organization. World malaria report 2020: 20 years of global progress and challenges. Geneva; 2020.

2. World Health Organization, Global Malaria Programme. Guidelines for the treatment of malaria. Geneva;. 2015. Available from: http://www.ncbi.nlm.nih.gov/books/NBK294440/ (accessed Jan 20, 2021)

3. World Health Organization. World malaria report 2019. Geneva; 2019.

4. WHO Guidelines for malaria. Geneva: World Health Organization; 2021. Available from: https://www.ncbi.nlm.nih.gov/books/NBK568494/

5. ACTwatch Group, Musuva A, Ejersa W, Kiptui R, Memusi D, Abwao E. The malaria testing and treatment landscape in Kenya: results from a nationally representative survey among the public and private sector in 2016. Malar J. 2017 21;16(1):494.

6. Prudhomme O'Meara W, Menya D, Laktabai J, Platt A, Saran I, Maffioli E, et al. Improving rational use of ACTs through diagnosis-dependent subsidies: Evidence from a cluster-randomised controlled trial in western Kenya. PLoS Med. 2018;15(7):e1002607.

7. Cohen J, Dupas P, Schaner S. Price Subsidies, Diagnostic Tests, and Targeting of Malaria Treatment: Evidence from a Randomised Controlled Trial. Am Econ Rev. 2015 Feb;105(2):609–45.

8. Briggs MA, Kalolella A, Bruxvoort K, Wiegand R, Lopez G, Festo C, et al. Prevalence of Malaria Parasitemia and Purchase of Artemisinin-Based Combination Therapies (ACTs)

For peer review only

among Drug Shop Clients in Two Regions in Tanzania with ACT Subsidies. PLOS ONE. 2014 Apr 14;9(4):e94074.

9. Mbonye AK, Lal S, Cundill B, Hansen KS, Clarke S, Magnussen P. Treatment of fevers prior to introducing rapid diagnostic tests for malaria in registered drug shops in Uganda. Malar J. 2013 Apr 16;12(1):131.

10. World Health Organization, Special Programme for Research and Training in Tropical Diseases, Foundation for Innovative New Diagnostics, Centers for Disease Control (U.S.). Malaria rapid diagnostic test performance: results of WHO product testing of malaria mRDTs : 4 (2012). [Internet]. Geneva, Switzerland: World Health Organization on behalf of the Special Programme for Research and Training in Tropical Diseases; 2012 [cited 2021 Jun 7]. Available from: http://apps.who.int/iris/bitstream/10665/77748/1/9789241504720_eng.pdf

11. Laktabai J, Lesser A, Platt A, Maffioli E, Mohanan M, Menya D, et al. Innovative public-private partnership to target subsidised antimalarials: a study protocol for a cluster randomised controlled trial to evaluate a community intervention in Western Kenya. BMJ Open. 2017 20;7(3):e013972.

12. Winn LK, Lesser A, Menya D, Baumgartner JN, Kipkoech Kirui J, Saran I, et al. Motivation and satisfaction among community health workers administering rapid diagnostic tests for malaria in Western Kenya. J Glob Health. 2018 Jun;8(1):010401.

For peer review only

For peer review only - http://bmjopen.bmj.com/site/about/guidelines.xhtml

13. Burnham KP, Anderson DR, Huyvaert KP. AIC model selection and multimodel inference in behavioral ecology: some background, observations, and comparisons. Behav Ecol Sociobiol. 2011 Jan 1;65(1):23–35.

14. Chibwana AI, Mathanga DP, Chinkhumba J, Campbell CH. Socio-cultural predictors of health-seeking behaviour for febrile under-five children in Mwanza-Neno district, Malawi. Malar J. 2009 Sep 24;8(1):219.

15. Cohen J, Cox A, Dickens W, Maloney K, Lam F, Fink G. Determinants of malaria diagnostic uptake in the retail sector: qualitative analysis from focus groups in Uganda. Malar J. 2015 Dec;14(1):89.

16. Kisia J, Nelima F, Otieno DO, Kiilu K, Emmanuel W, Sohani S, et al. Factors associated with utilization of community health workers in improving access to malaria treatment among children in Kenya. Malar J. 2012 Jul 30;11:248.

17. Mukanga D, Tibenderana JK, Peterson S, Pariyo GW, Kiguli J, Waiswa P, et al. Access, acceptability and utilization of community health workers using diagnostics for case management of fever in Ugandan children: a cross-sectional study. Malar J. 2012 May 24;11(1):121.

18. Win Han Oo, Gold L, Moore K, Agius PA, Fowkes FJI. The impact of community-delivered models of malaria control and elimination: a systematic review. Malar J. 2019 Aug 6;18(1):269.

For peer review only

For peer review only - http://bmjopen.bmj.com/site/about/guidelines.xhtml

557 19. Faust C, Zelner J, Brasseur P, Vaillant M, Badiane M, Cisse M, et al. Assessing Drivers of Full Adoption of Test and Treat policy for Malaria in Senegal. Am J Trop Med Hyg. 2015 Jul 8;93(1):159–67.

For peer review only

For peer review only - http://bmjopen.bmj.com/site/about/guidelines.xhtml

Coupon R01 CHV Participation Assessment

April 13, 2016

Note on administration: This survey is intended to be administered orally by an enumerator to a CHV.

I. Background Information

1) Sub-county:
    1. Bungoma East
    2. Kiminini

2) Name of CU that the CHV serves: ________________________

3) CHV's gender:
    1. Male
    2. Female

4) Age: ________ Years

5) Highest school level:
    1. None
    2. Some Primary (not completed Class 8)
    3. Primary completed
    4. Primary completed and some secondary (secondary not completed)
    5. Secondary completed
    6. College
    7. University

6) For how long have you served as a CHV?
    1. < 1
    2. > 1-3 Years
    3. > 3-5 Years
    4. > 5 Years

7) Apart from serving as a CHV, what other occupation(s) or role(s) do you have?

For peer review only

1. *Write-in response:* ________________________
2. None

8) How many trainings for CHV activities of any kind have you had since the RDT training for this malaria project?
    1. None
    2. One
    3. Two
    4. Three
    5. Four
    6. Five
    7. More than five

9) How long ago was your most recent training for CHV activities of any kind?
    1. Within the past week
    2. Between one week and one month ago
    3. Between one and three months ago
    4. Between three months and 1 year ago
    5. More than one year ago

10) What was the topic of your most recent training?
    1. Malaria (this project)
    2. Malaria (another project)
    3. Maternal health
    4. Polio campaign
    5. Other vaccination program
    6. Other child health topic not mentioned above

    7. Other: _______

II. Self-description of Role

11) In the past month, what activities have you carried out as a CHV overall?
(Enumerator: check all that are mentioned; do not read the list)
    1. Malaria testing
    2. Provide vouchers for malaria drugs
    3. Mosquito net distribution
    4. Provide other malaria-related services/counseling

For peer review only

5. Polio campaign participation
6. Other vaccination campaign(s)
7. Child health services not mentioned above
8. Maternal health services not mentioned above
9. Giving health education to households
10. None
11. Other: ______________________________
____
____

12) How many times have you met with your CHEW in the past 3 months?
1. Once
2. Twice
3. Three times
4. More than three times
5. None

13) What activities do you perform specifically for the malaria project?
(Enumerator: check all that are mentioned; do not read the list)
1. Perform malaria RDTs
2. Provide vouchers for malaria drugs
3. Provide malaria drugs
4. Provide referrals
5. Provide counseling / advice
6. Other: ____________________________________

14) For how many clients have you performed an RDT in the past month?
1. 1 client
2. 2-5 clients
3. 5 clients and above

15) What are the requirements for a client to receive an RDT under this malaria project?
(Enumerator: check all that are mentioned; do not read the list)
1. No requirements – anyone who asks can be tested
2. Must live in my CU
3. Has a documented fever (i.e., as measured by me)

For peer review only

4. Has a documented fever <u>or</u> reports a history of recent fever
5. Has a documented fever <u>or</u> reports a history of recent fever <u>or</u> reports any symptoms of malaria
6. Consent must be provided by the client (or by the parent/guardian of a minor)
7. Must not be visibly pregnant

8. Other: ___________________________________

16) What motivates you to participate in this malaria project?
1. Personal desire
2. Previous experience
3. Family
4. Organization (partner and GOK)
5. Monetary incentive (money given to group after 6 months and transport to attend supervision and supplies replenishment)
6. In-kind incentive (e.g., airtime for your phone)
7. Support from CHEWs
8. Support from link facilities in attending to clients referred for care
9. Other:____________________________

III. Satisfaction with Role

17) What are the bad things about participating in this project?
(Enumerator: check all that are mentioned; do not read the list)

(Open-ended response): ____

____

____

18) What are the good things about participating in this project?
(Enumerator: check all that are mentioned; do not read the list)

(Open-ended response): ____

____

For peer review only

<u>Please indicate your level of agreement with the following statements with regards to the malaria project **only.**</u>

Question	Strongly Agree	Somewhat Agree	50-50	Somewhat Disagree	Strongly Disagree
19) Overall, I am confident in my ability to perform my role in this malaria project.					
20) I receive enough supervision in my work from the malaria project study staff.					
21) I believe the clients that test positive for malaria follow the advice that I give them					
22) I believe the clients that test negative for malaria follow the advice that I give them					
23) The people in my community appreciate my work on the malaria project.					
24) I have gained more recognition in my community since I began testing for malaria.					
25) I am proud of the work that I do in the malaria project.					
26) I have gained valuable knowledge as a result of my role in this malaria project.					
27) Overall, I think the malaria project is an important activity for me to be involved in.					
28) Overall, I enjoy carrying out my role in this malaria project.					
29) I would like to continue with my role in this malaria project.					

For peer review only

30) *If the CHV answers "50-50", somewhat disagree" or "slightly disagree" to continuing their role in the project*, why?
(Enumerator: check all that are mentioned; do not read the list)

1. Requires too much time
2. Requires too much effort
3. Don't receive enough appreciation
4. Don't receive compensation
5. Frustrated that they can't provide drugs directly to clients
6. Conflicts with other CHV activities
7. Conflicts with non-CHV activities / responsibilities
8. Other ____

IV. Logistics & Challenges of Role

A. Implementation

i. Community awareness & trust of services

Question	Always aware	Usually aware	Sometimes aware	Rarely aware	Never aware
31) Of all of the clients that you provide services to for any reason, how often are the clients already aware that you provide RDT testing?					
32) Of the clients that visit you with a fever or other symptoms of malaria, how often are these clients already aware that you provide RDT testing?					
33) Of the clients that visit you with a fever or other symptoms of malaria, how often are these clients already aware that you provide vouchers for malaria drugs?					

34) How do you think your clients learn about the malaria services (testing and vouchers) that you provide?
(Enumerator: check all that are mentioned; do not read the list)

1. From me
2. From family members
3. From neighbors / friends
4. From village leaders
5. From health facility staff
6. From malaria project staff

7. Other: ____________________________

Please indicate your level of agreement with the following statements.

Question	Strongly Agree	Somewhat Agree	50-50	Somewhat Disagree	Strongly Disagree
35) The malaria RDT that I do can be trusted just as much as a malaria RDT done in a health facility.					
36) The clients that test positive for malaria trust the results of the test					
37) The clients that test negative for malaria trust the results of the test					
38) People I test for malaria believe I am actually testing for HIV.					

For peer review only

39) Do any of your clients refuse an RDT when you offer it?
1. Yes
2. No

40) *If Yes to above,* What are the reasons that you think these clients refuse?
(Enumerator: check all that are mentioned; do not read the list)
3. They don't think it's necessary
4. They don't trust me
5. They don't trust the test
6. They are already convinced they have malaria
7. They plan to buy antimalarial drugs regardless of test result
8. They don't want to be pricked
9. They think it may really be an HIV test

10. Other: ______________________

ii. Logistics & Challenges

41) What are all of the ways that clients find you to receive a malaria RDT?
(Enumerator: check all that are mentioned; do not read the list)
1. Clients visit me at my home
2. Clients visit me at a health facility

3. Clients visit me at church
4. I visit clients at their homes
5. Client visit me at another public location
6. Client visit me at another private location
7. Clients call my mobile phone
8. The study staff contacts me
9. The health facility contacts me

10. Other:______________________

42) Where do you provide testing for malaria? *(Enumerator: check all that are mentioned; do not read the list)*
1. At my home
2. At a health facility
3. At church
4. At client's home
5. At another public location
6. At another private location
7. At CHV Office
8. In Church
9. In Barazas (public meeting)

10. Other:______________________

43) How often do you lack all of the supplies you need to perform the malaria services of testing and providing vouchers when necessary?
1. Always
2. Sometimes
3. 50-50
4. Rarely
5. Never

44) Which, if any, supplies related to the malaria services you perform for this project have you had a stock-out of in the past month?
(Enumerator: check all that are mentioned; do not read the list)
1. RDTs
2. Buffer
3. Lancets
4. Alcohol swabs
5. Cotton wool
6. Gloves
7. Safety disposal box
8. Yellow waste bags
9. Plastic (zip-loc) bags
10. Thermometer
11. Thermometer batteries

12. Pen
13. Encounter forms
14. Voucher forms

15. Other:____________________________

45) How would prefer your consumption of malaria project supplies be monitored?
1. CHV counts the supplies physically
2. CHV references filled encounter forms
3. CHV uses inventory card to document consumption of supplies
4. CHV relies on the supervision team to monitor the supplies

46) What are the main challenges and barriers you face in implementing the malaria project? *(Enumerator: check all that are mentioned; do not read the list)*
1. Community is unaware that I offer malaria RDTs
2. Community is unaware that I offer vouchers for malaria drugs
3. The clients don't always trust the test results I give them
4. The clients are unhappy that I can't provide the malaria drugs to them directly
5. Transport
6. Not enough training
7. Not enough supervision from study staff
8. Not enough supervision from CHEWs
9. Lack of inventory systems to monitor supplies
10. Competing activities within the community
11. Other roles by other NGOs interfere with Malaria project
12. My other main occupation does not give me time to participate in Malaria project
13. Very few clients coming for test
14. Nature of the community not willing to interact with CHVs
15. Cultural practices e.g use of herbal medicine, seeking help from traditional healers
16. Level of education of community member (the less educated don't understand the importance of malaria test and the very educated not willing to be tested by the community health volunteer
17. Lack of mobilization at the community
18. Family relationships are affected
19. Other: __________________________________

47) Does frequent supervision improve your ability to provide quality malaria testing?
1. Yes
2. No

48) *If yes above*, how frequently would you prefer supervision to be carried out?
1. Once a week
2. Once every two weeks
3. Once a month
4. Once every two months

49) What is the main purpose of supervision activities conducted by the malaria project team?
(Enumerator: check only one)
1. To monitor whether I make any mistakes
2. To provide additional training
3. To ensure data quality

50) What would help you perform your role in the malaria project better?
(Enumerator: check all that are mentioned; do not read the list)
1. More training
2. More supervision from study team
3. More supervision from CHEWs
4. More regular restocking of supplies
5. Raising community awareness about the project
6. Compensation for time spent
7. Sharing my CHV duties with another CHV
8. Other: ________________________________

iii. Time required

51) On average, how long does it take you to complete a visit with a patient complaining of malaria symptoms?

____________________ Minutes

52) In the last week, what portion of your CHV activities have been related to the malaria project?
1. Less than half
2. About half
3. More than half

53) How many hours do you think you spend on the malaria project activities in an average week (including seeing clients, filling forms, meeting with malaria study staff, etc.)?

____________________ Hours

54) How many hours do you think you spend on all of your other CHV activities and responsibilities in an average week (including seeing clients, reporting, restocking, trainings, etc.)?

____________________ Hours

55) Do you think the total time you spend on all CHV activities has decreased, increased, or stayed about the same since you began the malaria project activities?

For peer review only

1. Decreased
2. Stayed about the same
3. Increased

B. Relation to other CHV activities

56) Has participating in the malaria project had an effect on your other CHV activities?
    1. Yes
    2. No

57) If yes, in what ways:
(Enumerator: check all that are mentioned; do not read the list)
    1. Improved skills applicable to other CHV activities
    2. More clients visit me for non-malaria services
    3. Less time to complete other CHV activities

    4. Other: ____________________

58) Please indicate to what extent you agree with the statement: Due to my involvement with the malaria project, I now provide more non-malaria health services than before the malaria project.
    1. Strongly agree – I provide much more non-malaria health services than before
    2. Somewhat agree
    3. Neither agree nor disagree – it has stayed the same
    4. Somewhat disagree
    5. Strongly disagree – I provide much less non-malaria health services than before

(Only ask the next 2 questions if "strongly agree" or "somewhat agree" above):

59) If "strongly agree" or "somewhat agree" above: What are the other (non-malaria) services you provide more of?
(Enumerator: check all that are mentioned; do not read the list)
    1. Maternal health services
    2. Vaccinations
    3. Providing health information
    4. *… (Other typical CHV activities to include in this list?)*

    5. Other: ___________________________

60) To whom do you provide more non-malaria services than before the malaria project started? (select all that apply):
(Enumerator: check all that are mentioned; do not read the list)
    1. Men
    2. Women

For peer review only

3. Children

61) Please indicate to what extent you agree with the statement: There are other CHV activities or services I would normally have performed in the last month that I did not because the malaria project took too much of my time.
1. Strongly agree
2. Somewhat agree
3. 50-50
4. Somewhat disagree
5. Strongly disagree

62) If yes, what activities / services?
(Enumerator: check all that are mentioned; do not read the list)
1. Maternal health services
2. Vaccinations
3. Providing health information
4. … (Other typical CHV activities to include in this list?)

5. Other: ___________________________

63) Please indicate to what extent you agree with the statement: There are other responsibilities outside of my work as a CHV that I would normally have performed in the last month that I did not because my work as a CHV took too much of my time.
1. Strongly agree
2. Somewhat agree
3. 50-50
4. Somewhat disagree
5. Strongly disagree

64) *If "strongly agree" or "somewhat agree" above:* What responsibilities outside of your role as a CHV have been affected? (childcare, other volunteering, church, education/study, family, etc.)
(Enumerator: check all that are mentioned; do not read the list)
1. Childcare
2. Family responsibilities
3. Other work
4. Other volunteering / community activities
5. Religious activities

6. Other: ___________________________

65) What, if an, relationships have been affected by your work as a CHV on this malaria project? *(Enumerator: check all that are mentioned; do not read the list)*
1. Children
2. Spouse
3. Siblings
4. Parents
5. Other family members not mentioned above

For peer review only

6. Neighbors
7. Friends
8. Other: ___________________________

66) What do you think are the most important services you provide as a CHV?
(Enumerator: check all that are mentioned; do not read the list)

1. Malaria testing
2. Provide vouchers for malaria drugs
3. Mosquito net distribution
4. Provide other malaria-related services/counseling
5. Polio campaign participation
6. Other vaccination campaign(s)
7. Child health services not mentioned above
8. Maternal health services not mentioned above
9. Giving health education to households
10. None
11. Other: ___________________________
____
____

67) What do you think is the most important service you provide as a CHV?
(Enumerator: Only allow one answer)

1. Malaria testing
2. Provide vouchers for malaria drugs
3. Mosquito net distribution
4. Provide other malaria-related services/counseling
5. Polio campaign participation
6. Other vaccination campaign(s)
7. Child health services not mentioned above
8. Maternal health services not mentioned above
9. Giving health education to households
10. None
11. Other: ___________________________
____
____

68) In order to monitor the quality of the tests done, some CHVs in this project were randomly chosen to use a device called a DekiReader that automatically reads the RDT cassette and displays the results. Did you use this device?

1. Yes
2. No
3. I was selected but I couldn't use it (too difficult)
4. Not sure

If 1 or 3, ask next 3 questions:

69) To what extent would you agree with the statement, "The DekiReader was easy to use correctly"?

1. Strongly agree
2. Somewhat agree
3. 50-50
4. Somewhat disagree
5. Strongly disagree

70) To what extent would you agree with the statement, "The Deki Reader helped me improve my ability to perform RDTs correctly"?
    1. Strongly agree
    2. Somewhat agree
    3. 50-50
    4. Somewhat disagree
    5. Strongly disagree

71) If you had the option to use the Deki Reader for every test as part of your routine work, would you want to have it or not?
    1. Yes
    2. No
    3. Don't Know / Unsure

72) Do you have any concerns or suggestions to improve the implementation of the malaria project?
    (Open-ended response): ____

    ____

    ____

    ____

Thank you for your participation and feedback!

For peer review only

Checklist for performing Rapid Diagnostic Testing correctly

Name of CHV ____________________ Community Unit ___________________

Name of observer____________________________ Date _____________________

No.	Task	Task Category	Correctly done No = 0 Yes =1	Observer's comments
**A.**	**RDT preparation**			
1.	Assembles RDT packet, buffer, swab, lancet			
2.	Read expiration date			
3.	Remove contents of test packet			
4.	Write patient's name on cassette			
**B.**	**Patient preparation**			
5.	Identify patient's details and record patient's name and date on the RDT cassette			
6.	Explain procedure to patient. Provide reassurance, as needed			
7.	Wear gloves	Safety		
**C.**	**Blood collection + dispensing**			
8.	Select 4th finger of left hand for blood collection			
9.	Clean finger with alcohol swab and allow it to dry	Safety		
10.	Prick finger firmly with sterile lancet	Safety		
11.	Discard lancet in sharps bin immediately after pricking finger. Do not set it down first	Safety		
12.	DO NOT squeeze finger excessively			
13.	Collect an adequate volume of blood with pipette	Accuracy		
14.	Dispense blood in correct well	Accuracy		
15.	Discards the pipette in the sharps box	Safety		
16.	Dispose of gloves, wrappers, alcohol swab, loop, desiccant and cassette in non-sharps container	Safety		
**D.**	**RDT procedure and reading results**			
17.	Dispense correct volume of buffer	Accuracy		
18.	Wait for twenty (20) minutes	Accuracy		
19.	Verify internal test control			
20.	Read results correctly	Accuracy		
**E.**	**Recording results**			
21.	Records date and time correctly on encounter form	Treatment		
**F.**	**Deliver the results**			
22.	Explain to the patient the results and what they mean	Treatment		

For peer review only

A. *Household survey instrument* (Note: the survey will be administered electronically)

1. Date of interview (DD/MM/YY):

☐☐ / ☐☐ / 20 ☐☐

2. Name of interviewer: ____________

3. Interviewer ID: ☐☐

CU – dropdown menu

Demographics

4. Location: ____________

5. Sub-location: ____________

6. Village name: ____________

Eligibility criteria

7. Did any member of the household have fever or malaria-like illness in the last four weeks? *If no, stop.*
   1. Yes
   2. No
   3. Don't Know

8. How long ago did the most recent fever start?

☐☐ Days

INSTRUCTION: if the ill individual is 18 or older and not present, stop and save the interview now; recording the time and date saved on your notebook; return to the saved interview if the ill adult is present on a return visit, use the recorded time and date to locate the form.

9. Are you still ill today?
   1. Yes
   2. No

Patient information

10. Gender of patient:

For peer review only - http://bmjopen.bmj.com/site/about/guidelines.xhtml

For peer review only

1. Male
2. Female

11. Relationship of patient to household head
    1. Head
    2. Spouse of head
    3. Child
    4. Parent
    5. Grandparent
    6. Grandchild
    7. Aunt /Uncle
    8. Brother / Sister
    9. Cousin
    10. Mother
    11. Father
    12. Employee
    13. OTHER____________________

12. Age of patient: [|] Years (if less than 18, skip to respondent information, question 15)

13. Highest school level:
    1. None
    2. Some Primary (not completed Class 8)
    3. Primary completed
    4. Primary completed and some secondary (secondary not completed)
    5. Secondary completed
    6. College
    7. University

14. Occupation
    1. Farming/Livestock keeping
    2. Home maker
    3. Student
    4. Paid employee
    5. Casual employee
    6. Self-employed (not agriculture, livestock) without employees
    7. Self-employed (not agriculture, livestock) with employees
    8. Unpaid family helper in a business
    9. Not working
    10. Unable to work/disabled
    11. Other (Specify)

Respondent (Answer if age of patient is <18 years. If patient is >18 years, skip to question 19.)

15. Age of respondent

For peer review only - http://bmjopen.bmj.com/site/about/guidelines.xhtml

☐☐ Years

16. Highest school level:
    1. None
    2. Some Primary (not completed Class 8)
    3. Primary completed
    4. Primary completed and some secondary (secondary not completed)
    5. Secondary completed
    6. College
    7. University

17. Gender of respondent:
    1. Male
    2. Female

18. Relationship of respondent to child:
    1. Parent
    2. Aunt/Uncle
    3. Grandparent
    4. Sibling
    5. Other

Endpoints *Note: The wording of the questions in this section are directed at a respondent 18 or over; when administered as an electronic survey, there will be two parallel sets of questions that branch based on whether a child or adult was the patient.*

19. How severe was the illness?
    1. Very severe
    2. Severe
    3. Moderately severe
    4. Mild
    5. Very mild

20. Did you take any action for your illness? *Note: taking drugs at home is an action. If "Don't remember" or "No", skip to COVARIATES section, question 56.)*
    1. Yes
    2. No
    3. Don't remember

21. What did you do FIRST for your illness? *Only one answer*
    1. Contact CHW

For peer review only - http://bmjopen.bmj.com/site/about/guidelines.xhtml

2. Go to a government health centre
3. Go to a government dispensary
4. Go to a government hospital
5. Go to a private clinic or mission facility
6. Go to a shop or pharmacy
7. Take some drugs at home
8. visit traditional healers
9. visit religious/cultural healers
10. OTHER: ____________

22. What were the MAIN reasons for deciding to do that? *Multiple answers OK*
    1. I already had the drugs at home
    2. It is the nearest place (facility or drug shop)
    3. The services are free
    4. Money- this was most affordable
    5. Wanted to see a Doctor
    6. Illness very serious
    7. Illness not very serious
    8. Time was limited / this was the fastest
    9. Good quality service
    10. There are no drugs in the facility
    11. There is laboratory testing
    12. OTHER: ____________

23. How long after the illness began did you do that?
    1. same day
    2. next day
    3. after two days
    4. more than two days later
    5. OTHER: ____________

24. Did you take any other action? *If no, skip to question 26.*
    1. Yes
    2. No
    3. Don't remember

25. If yes, what did you do? *Allow multiple answers*

    1. Contact CHW
    2. Go to a government health centre
    3. Go to a government dispensary
    4. Go to a government hospital
    5. Go to a private clinic or mission facility
    6. Go to a shop or pharmacy

For peer review only - http://bmjopen.bmj.com/site/about/guidelines.xhtml

7. Take some drugs at home
8. visit traditional healers
9. visit religious/cultural healers
10. OTHER: ___________

26. How long did you take to fully recover?

[|] Days (99=not yet recovered)

27. Did you have any laboratory test done during your illness? *PROBE. Ask about blood, stool or urine taken to help find out about the illness*

1. Yes
2. No
3. Don't remember

28. Did you have a malaria test? *If no or don't remember, skip to question 42.*

1. Yes
2. No
3. Don't remember
4. Don't know

29. How long after the start of the illness did you have a malaria test (the first test, if more than one)?

1. same day
2. next day
3. after two days
4. OTHER: ___________

30. Was it a microscopy test or a rapid diagnostic test? (If both, record in 'OTHER')

1. RDT
2. microscopy
3. Don't remember or don't know
4. OTHER (specify): _____________

31. Where was the malaria test done?

1. CHW (go to Question 32, else skip to 35.)
2. shop or pharmacy
3. private clinic/hospital
4. government dispensary
5. government hospital
6. government health center
7. private lab
8. OTHER: ___________

For peer review only - http://bmjopen.bmj.com/site/about/guidelines.xhtml

32. If a CHW did the test, where was it done?
    1. In my village
    2. In another village but the same CU (in which the survey is being conducted)
    3. Another CU (write name: ___________)
    4. Don't know/don't remember

33. Did the CHW give you a voucher to purchase a drug?
    1. Yes (Ask question 34 else skip to 35.)
    2. No
    3. Don't know/don't remember

34. Did you use the voucher to purchase a drug?
    1. Yes
    2. No
    3. Don't know/ don't remember

35. How much did the malaria test cost (Ksh.)? If more than one test, list both
    ______________**Free text**

36. What were the results of the malaria test? (If more than one test, use OTHER)
    1. Positive
    2. Negative
    3. Don't remember / Don't know
    4. OTHER________________

37. How likely is it that the result of your malaria test was correct?
    1. Very likely
    2. Likely
    3. 50-50
    4. Unlikely
    5. Very unlikely
    6. Don't know
    7. OTHER (use if have different beliefs about multiple tests)

38. Record observed for test? *Interviewer question (this can include study forms)*
    1. Yes
    2. No (Skip to question 42.)

If yes (record observed), interviewer should answer the next 3 questions:

39. Where does the record report the test was done? (multiple answers possible)

For peer review only - http://bmjopen.bmj.com/site/about/guidelines.xhtml

1. CHW
2. shop or pharmacy
3. private clinic/hospital
4. government dispensary
5. government hospital
6. government health center
7. private lab
8. Not specified / Cannot be determined
9. OTHER: ____________

40. What type of malaria test does the record report was done? (if more than one test, use OTHER)

1. RDT
2. microscopy
3. Not specified / Cannot be determined
4. OTHER (specify): ______________

41. What were the results of the malaria test according to the record? (if more than one test, use OTHER)

1. Positive
2. Negative
3. Not specified / Cannot be determined
4. OTHER ____________________

42. How likely is it that the illness was malaria?
1. Very likely
2. Likely
3. 50-50
4. Unlikely
5. Very unlikely
6. Don't know
7. No response

43. Were any drugs taken for the illness? *If no or don't remember, skip to question 55.*
1. Yes
2. No
3. Don't remember

44. Which medicines did you take? (Multiple answers possible) SKIP PATTERN: If 1 or 4 (ACT), continue with next question. Else, skip to 53

1. Coartem/Artefan/other AL
2. Quinine

For peer review only - http://bmjopen.bmj.com/site/about/guidelines.xhtml

For peer review only

3. SP/Fansidar/Metakelfin/Moladar
4. Other ACT (e.g Duocotexin)
5. Amoxyl
6. Septrin
7. Ciprofloxacin/Norfloxaccin
8. Panadol/Brufen/Hedex/Painkiller/Action/Maramoja
9. Don't know/Don't Remember
10. Other (specify: __________)

45. Packaging observed for ACT? *Interviewer question. If YES, ask question 46. If NO, skip to question 47.*
   1. Yes
   2. No

46. If ACT package observed, what brand? *Interviewer question*.
   1. Coartem (AL)
   2. Aretefan (AL)
   3. Duocotexin
   4. P-Alaxin
   5. AL (IPCA brand)
   6. AQ AS
   7. Lonart (AL)
   8. Artequick
   9. Lumartem (AL)
   10. Combiart (AL)
   11. L-artem (AL)
   12. OTHER: ___________

47. How long after the start of the illness did you start taking the ACT?
   1. same day
   2. next day
   3. after two days
   4. OTHER: ___________

48. Did you start taking the ACT before or after the malaria test? *Only ask this if YES to 28.*
   1. Before
   2. After
   3. Don't remember
   4. Not Applicable

If respondent answered "1-Coartem/Artefan/other AL" to question 44, ask questions 49-52, else skip to question 53.

49. If AL, how many pills were given? *Only ask if 44 = 1 (AL ONLY)*

For peer review only - http://bmjopen.bmj.com/site/about/guidelines.xhtml

1. 6
2. 12
3. 18
4. 24
5. Don't know/remember
6. OTHER: ___________

50. If AL, did you / patient take all of the tablets? *Only ask if 44 = 1 (AL ONLY)*

1. Yes
2. No
3. Not sure
4. Don't remember
5. Not yet—still taking them

51. Where was the AL obtained? MULTIPLE RESPONSES POSSIBLE
(If the drugs were already in the home at the onset of illness, record this but find out from where they were originally obtained) Only ask if 44 = 1, 4 ie any ACT.

1. Used drugs at home
2. Pharmacy, chemist or duka la dawa with a prescription
3. Pharmacy, chemist or duka la dawa; no prescription
4. CHW
5. Neighbor
6. Private health facility
7. Government health facility
8. general store
9. OTHER: _______________
10. Don't remember/don't know

52. Did you get AL at the first place you sought treatment? *Only ask if 44 = 1,4 (ACT ONLY)*

1. Yes
2. No
3. Yes and another place
4. Don't know/don't remember

53. How likely do you think the malaria drugs you took were effective? (ask if respondent answered 1, 2, 3 or 4 to question 44.)

1. Very likely
2. Likely
3. 50-50
4. Unlikely

For peer review only

For peer review only - http://bmjopen.bmj.com/site/about/guidelines.xhtml

5. Very unlikely
6. Don't know
7. No response

54. How much did you spend for all the drugs you purchased for this illness? (-1 should be used for 'I don't know/don't remember')

1. ___________________________

55. How much did you spend in total to take care of this illness, including testing, drugs, transportation, admission fees, registration and consultation fees? (-1 should be used for 'I don't know/don't remember')

1. ____________________________

Covariates *THESE QUESTIONS DIRECTED AT RESPONDENT, EVEN IF NOT THE PATIENT*

56. GPS coordinates: _____ (PLACED AT THE END OF THE ELECTRONIC SURVEY)

57. Does your household have any mosquito nets? (*If no or don't know, skip to question 60.*)

1. Yes
2. No
3. DK

58. Did THE PATIENT sleep under the net last night?

1. Yes
2. Has a net for sleeping space, but did not use it last night.
3. Does not have net for sleeping space
4. Do not Remember/Don't Know

59. Was the net pre-treated with insecticide when you got it?

1. Yes
2. No
3. Not Applicable (no net for sleeping space)
4. Do not Remember/Don't Know

60. Is there a CHW in your village that provides any services or health education? *If no or do not know skip to question 66.*

1. Yes
2. No
3. Don't Know

61. Which services does the CHW provide? *Multiple responses possible*

1. Information

For peer review only - http://bmjopen.bmj.com/site/about/guidelines.xhtml

2. Referral
3. Polio immunization
4. Advice
5. Testing for HIV
6. Testing for malaria
7. Testing for diabetes
8. Testing for hypertension
9. Testing for pregnancy
10. Coupon for antimalarial drug
11. Bednets
12. Water treatment (ie Lifestraw)
13. Other (specify) ___________________________

62. Does your CHW provide malaria diagnostic testing?
    1. Yes
    2. No
    3. Don't know/Don't remember

63. Does your CHW provide a discount on a malaria drug in case the test is positive for malaria?
    1. Yes
    2. No
    3. Don't know/Don't remember

64. Have you ever contacted a CHW near you in case of illness for yourself or someone in your household?
    1. Yes
    2. No (*skip to question 66*)
    3. DR/DK (*skip to question 66*)

65. What did the CHW do? (multiple responses possible)
    1. Refer
    2. Give medicine
    3. Accompany to shop
    4. Give advice
    5. Perform a malaria test
    6. Give a coupon for an antimalarial drug
    7. Other (specify)____________________

66. Do you know what a rapid diagnostic test for malaria is? *Show example of cassette*
    1. Yes
    2. No → skip to 71

67. Have you ever had an RDT test for malaria?
    1. Yes
    2. No
    3. Don't know/don't remember

68. Do you know anyone besides one of your household members who has had an RDT?
    1. Yes
    2. No

For peer review only - http://bmjopen.bmj.com/site/about/guidelines.xhtml

3. Don't know/don't remember

69. If you have fever and your malaria RDT is negative, how likely is it that the test is correct?

1. Very likely
2. Likely
3. 50-50
4. Unlikely
5. Very unlikely
6. Don't know
7. No response

70. If you have fever and your malaria RDT is positive, how likely is it that the test is correct?

1. Very likely
2. Likely
3. 50-50
4. Unlikely
5. Very unlikely
6. Don't know
7. No response

71. Is malaria common in your village

1. Yes
2. No
3. Don't know/not sure

72. If ten people (watu) from your village have fever, how many of them are likely to have malaria? ______________(number 0-10 or 99 for don't know)

73. If you have malaria, and you take AL, how likely is it that you will be completely better in 3 days?

1. Very likely
2. Likely
3. 50-50
4. Unlikely
5. Very unlikely
6. Don't know
7. No response

SES Questions
(Source:pilot)

74. Does your household have the following items

1. Electricity
2. Television
3. Refrigerator
4. Radio

For peer review only - http://bmjopen.bmj.com/site/about/guidelines.xhtml

5. Mobile phone
6. Motorcycle
7. car/truck
8. Bank account
9. None

75. How many of the following livestock does your household have
    1. Cows
    2. Sheep
    3. Goats
    4. Pigs
    5. Donkeys

For peer review only

76. What kind of toilet does your household have
    1. Flush or pour flush toilet
    2. VIP/Ventilated improved pit
    3. Pit latrine WITH slab
    4. Pit latrine without slab
    5. Composting toilet
    6. Bucket toilet
    7. No facility/bush/field
    8. Other

77. Main source of drinking water for your household
    1. Piped water/public tap/borehole
    2. Unprotected well
    3. Protected well
    4. Protected spring
    5. Unprotected spring
    6. Rain Water
    7. River Water
    8. Other:______________

78. Main material of the floor in your house
    1. Earthen
    2. Cement
    3. Floor tiles
    4. Wood planks
    5. Polished wood
    6. Other

79. Main material of the walls in your house
    1. Stone
    2. Brick
    3. Timber
    4. Iron Sheet
    5. Mud

For peer review only - http://bmjopen.bmj.com/site/about/guidelines.xhtml

6. Wood
7. Cement
8. Other

80. Main material of the roof of your house
    1. Iron sheets
    2. Roof tiles
    3. Grass thatched
    4. Wood

81. How many acres/hectares/feet of land for farming does your household own
    1. None
    2. Acres: ____________
    3. Hectares: ________
    4. Square feet(xx by xx):_________

For peer review only

For peer review only - http://bmjopen.bmj.com/site/about/guidelines.xhtml

STROBE Statement—Checklist of items that should be included in reports of ***cross-sectional studies***

	Item No	Recommendation	Page No
**Title and abstract**	1	(*a*) Indicate the study's design with a commonly used term in the title or the abstract	1
		(*b*) Provide in the abstract an informative and balanced summary of what was done and what was found	2-3
**Introduction**			
Background/rationale	2	Explain the scientific background and rationale for the investigation being reported	4-5
Objectives	3	State specific objectives, including any prespecified hypotheses	5
**Methods**			
Study design	4	Present key elements of study design early in the paper	6
Setting	5	Describe the setting, locations, and relevant dates, including periods of recruitment, exposure, follow-up, and data collection	6-7
Participants	6	(*a*) Give the eligibility criteria, and the sources and methods of selection of participants	8
Variables	7	Clearly define all outcomes, exposures, predictors, potential confounders, and effect modifiers. Give diagnostic criteria, if applicable	10
Data sources/ measurement	8*	For each variable of interest, give sources of data and details of methods of assessment (measurement). Describe comparability of assessment methods if there is more than one group	*9-10*
Bias	9	Describe any efforts to address potential sources of bias	
Study size	10	Explain how the study size was arrived at	8
Quantitative variables	11	Explain how quantitative variables were handled in the analyses. If applicable, describe which groupings were chosen and why	10
Statistical methods	12	(*a*) Describe all statistical methods, including those used to control for confounding	11-12
		(*b*) Describe any methods used to examine subgroups and interactions	
		(*c*) Explain how missing data were addressed	
		(*d*) If applicable, describe analytical methods taking account of sampling strategy	11
		(*e*) Describe any sensitivity analyses	
**Results**			
Participants	13*	(a) Report numbers of individuals at each stage of study—eg numbers potentially eligible, examined for eligibility, confirmed eligible, included in the study, completing follow-up, and analysed	14
		(b) Give reasons for non-participation at each stage	
		(c) Consider use of a flow diagram	
Descriptive data	14*	(a) Give characteristics of study participants (eg demographic, clinical, social) and information on exposures and potential confounders	14
		(b) Indicate number of participants with missing data for each variable of interest	
Outcome data	15*	Report numbers of outcome events or summary measures	15, Table 1

For peer review only

For peer review only - http://bmjopen.bmj.com/site/about/guidelines.xhtml

Main results	16	(*a*) Give unadjusted estimates and, if applicable, confounder-adjusted estimates and their precision (eg, 95% confidence interval). Make clear which confounders were adjusted for and why they were included	Table 2-6
		(*b*) Report category boundaries when continuous variables were categorized	Tables
		(*c*) If relevant, consider translating estimates of relative risk into absolute risk for a meaningful time period	
Other analyses	17	Report other analyses done—eg analyses of subgroups and interactions, and sensitivity analyses	
**Discussion**			
Key results	18	Summarise key results with reference to study objectives	18
Limitations	19	Discuss limitations of the study, taking into account sources of potential bias or imprecision. Discuss both direction and magnitude of any potential bias	20
Interpretation	20	Give a cautious overall interpretation of results considering objectives, limitations, multiplicity of analyses, results from similar studies, and other relevant evidence	21
Generalisability	21	Discuss the generalisability (external validity) of the study results	20
**Other information**			
Funding	22	Give the source of funding and the role of the funders for the present study and, if applicable, for the original study on which the present article is based	22

*Give information separately for exposed and unexposed groups.

Note: An Explanation and Elaboration article discusses each checklist item and gives methodological background and published examples of transparent reporting. The STROBE checklist is best used in conjunction with this article (freely available on the Web sites of PLoS Medicine at http://www.plosmedicine.org/, Annals of Internal Medicine at http://www.annals.org/, and Epidemiology at http://www.epidem.com/). Information on the STROBE Initiative is available at www.strobe-statement.org.

For peer review only

BMJ Open

Supply and demand-side factors influencing uptake of malaria testing services in the community: lessons for scale-up from a post-hoc analysis of a cluster-randomised, community-based trial in western Kenya.

Journal:	*BMJ Open*
Manuscript ID	bmjopen-2022-070482.R2
Article Type:	Original research
Date Submitted by the Author:	08-May-2023
Complete List of Authors:	Kirui, Joseph; Academic Model Providing Access to Healthcare, AMPATH<br>Malinga, Josephine; Duke Global Health Institute<br>Sang, Edna; Duke Global Inc, Kenya<br>Ambani, George; Academic Model Providing Access to Healthcare<br>Abel, Lucy; Academic Model Providing Access to Healthcare<br>Nalianya, Erick; Duke Global Inc, Kenya<br>Namae, Jane; Moi University College of Health Sciences<br>Boyce, Matthew; Georgetown University<br>Laktabai, Jeremiah; Academic Model Providing Access to Healthcare; Moi University College of Health Sciences<br>Menya, Diana; Moi University School of Public Health<br>O'Meara, Wendy; Duke Global Health Institute, ; Moi University,
**Primary Subject Heading**:	Global health
Secondary Subject Heading:	Health services research, Infectious diseases
Keywords:	Public health < INFECTIOUS DISEASES, Community child health < PAEDIATRICS, International health services < HEALTH SERVICES ADMINISTRATION & MANAGEMENT

SCHOLARONE™
Manuscripts

I, the Submitting Author has the right to grant and does grant on behalf of all authors of the Work (as defined in the below author licence), an exclusive licence and/or a non-exclusive licence for contributions from authors who are: i) UK Crown employees; ii) where BMJ has agreed a CC-BY licence shall apply, and/or iii) in accordance with the terms applicable for US Federal Government officers or employees acting as part of their official duties; on a worldwide, perpetual, irrevocable, royalty-free basis to BMJ Publishing Group Ltd ("BMJ") its licensees and where the relevant Journal is co-owned by BMJ to the co-owners of the Journal, to publish the Work in this journal and any other BMJ products and to exploit all rights, as set out in our licence.

The Submitting Author accepts and understands that any supply made under these terms is made by BMJ to the Submitting Author unless you are acting as an employee on behalf of your employer or a postgraduate student of an affiliated institution which is paying any applicable article publishing charge ("APC") for Open Access articles. Where the Submitting Author wishes to make the Work available on an Open Access basis (and intends to pay the relevant APC), the terms of reuse of such Open Access shall be governed by a Creative Commons licence – details of these licences and which Creative Commons licence will apply to this Work are set out in our licence referred to above.

Other than as permitted in any relevant BMJ Author's Self Archiving Policies, I confirm this Work has not been accepted for publication elsewhere, is not being considered for publication elsewhere and does not duplicate material already published. I confirm all authors consent to publication of this Work and authorise the granting of this licence.

For peer review only

Supply and demand-side factors influencing uptake of malaria testing services in the community: lessons for scale-up from a post-hoc analysis of a cluster-randomised, community-based trial in western Kenya.

Authors and affiliations:

Joseph Kipkoech[1*], Josephine Malinga[2], Edna Sang[3], George Ambani[1], Lucy Abel[1], Erick Nalianya[3], Jane Namae[4], Matthew Boyce[5], Jeremiah Laktabai[1,4], Diana Menya[6], Wendy Prudhomme O'Meara[2,6]

[1]Academic Model Providing Access to Healthcare, Eldoret, Kenya

[2]Duke Global Health Institute, Durham, North Carolina, USA.

[3]Duke Global Inc, Kenya.

[4]School of Medicine, Moi University College of Health Sciences, Eldoret Kenya.

[5]Georgetown University, Washington DC, USA.

[6]School of Public Health, Moi University College of Health Sciences, Eldoret, Kenya.

[*]Corresponding author

Keywords: Community-based programs, demand and supply, health interventions, malaria, diagnostic testing, Community Health Workers

List of abbreviations: Artemisinin Combination Therapy (ACT), Artemether Lumefantrine (AL) Community Health Extension Worker(CHEW), Community Health Worker (CHW), Community Unit (CU), Global Positioning System (GPS), Ministry of Health (MOH), Malaria Rapid Diagnostic Test (mRDT), World Health Organization (WHO)

For peer review only

For peer review only - http://bmjopen.bmj.com/site/about/guidelines.xhtml

Corresponding Author: Joseph Kipkoech, Email: josepheddykipkoech@gmail.com

Abstract

Objectives: Maximizing the impact of community-based programs requires understanding how the supply of, and demand for, the intervention interact at the point of delivery.

Design: We present a post-hoc analysis from a large-scale community health worker (CHW) study designed to increase the uptake of malaria diagnostic testing.

Setting: Respondents were identified during a household survey in western Kenya between July 2016 and April 2017.

Participants: Household members with fever in the last four weeks were interviewed at 12 and 18-months post-implementation. We collected monthly testing data from 244 participating CHWs and conducted semi-structured interviews with a random sample of 70 CHWs.

Primary and secondary outcome measures: The primary outcome measure was diagnostic testing before treatment for a recent fever. The secondary outcomes were receiveing a test from a CHW and tests done per month by each CHW.

Results: 55%(n=948/1738) reported having a malaria diagnostic test for their recent illness of which 39% were tested by a CHW. Being aware of a local CHW(Adj.OR=1.50, 95%CI:1.10-2.04) and belonging to the wealthiest households(vs. least wealthy) was associated with higher testing(Adj.OR=1.53, 95%CI:1.14-2.06). Wealthier households were *less* likely to receive their test from a CHW compared to poorer households(Adj.OR=0.32, 95%CI:0.17-0.62). Confidence

For peer review only - http://bmjopen.bmj.com/site/about/guidelines.xhtml

in artemether-lumefantrine(AL) to cure malaria(Adj.OR=2.75, 95%CI:1.54-4.92) and perceived accuracy of an mRDT(Adj.OR=2.43, 95%CI:1.12-5.27) were positively associated with testing by a CHW. Specific CHW attributes were associated with higher monthly number of tests included formal employment, serving more than 50 households(vs<50) and serving areas with a higher test positivity. On demand-side, confidence of the respondent in a test performed by a CHW was strongly associated with higher mRDT testing rates.

Conclusion: Scale-up of community-based malaria testing is feasible and effective in increasing uptake among the poorest households. To maximize impact, it is important to recognize factors that may restrict delivery and demand for such services.

Strengths and limitations of this study

- This analysis draws on a large multidimensional dataset describing uptake of community-based services and delivery of services in the context of a cluster-randomised implementation trial
- These data include population groups that were successfully reached with the intervention as well as those that may require alternative approaches in order to achieve adequate coverage of diagnostic testing before treatment with antimalarials.
- Triangulating data from the community (intervention recipient) and Community Health Workers (intervention delivery) allowed us to examine strengths and shortcomings from two unique perspectives
- As a secondary analysis of trial data, we were limited in the scope of the data available and not able to fully explore the interaction between the CHWs and households nor the complex determinants of health seeking behavior.

For peer review only

For peer review only - http://bmjopen.bmj.com/site/about/guidelines.xhtml

Introduction

Despite decades of control efforts, malaria remains a major public health problem. In 2019, an estimated 229 million cases of malaria occurred worldwide. Most malaria cases in 2019 were in the World Health Organization (WHO) African Region (215 million or 94%).[1] In the same year, there were an estimated 409 000 deaths from malaria globally, with Africa again accounting for 94%. [1] Children under 5 years are the most vulnerable group affected by malaria and in 2019, 67% of malaria deaths were in this group.[1]

Prompt malaria diagnosis either by microscopy or rapid diagnostic tests (mRDTs) is recommended by WHO for all patients with suspected malaria before they are given treatment.[2] Already, 95 countries have adopted the policy of testing all patients with suspected malaria before being treated, and this number is increasing. Diagnostic testing is free of charge in the public sector of almost 90 countries. [3] Early and accurate diagnosis is essential both for effective management of the disease and for strong malaria surveillance. Parasite-based diagnostic testing significantly reduces illness and death by enabling health providers to swiftly distinguish between malarial and non-malarial fevers and select the most appropriate treatment. [4]

The Kenya National Control Program adopted the Test and Treat policy in 2010 and this has been cascaded to the community level. The mRDTs are recommended for parasitological diagnosis in public health facilities where microscopy is not available and through Community Health Workers in some regions. Despite these efforts, most people in rural Kenya still opt for self-treatment and seek care in the retail health sector. [5,6] In our study setting, mRDT are not available in pharmacies and chemists, therefore, patients who purchase antimalarials in the retail sector receive no parasitological diagnosis before treatment. Studies have shown that targeting of antimalarials to true malaria cases in the retail sector is very low: as few as 20% of people purchasing

For peer review only

antimalarials are suffering from malaria [7,8] and as high as 70% of people with malaria do not purchase an ACT [9].

The deployment of mRDTs through trained community health workers could improve access to diagnosic testing for populations with limited access to health facilities. In a large, cluster-randomised trial[6], we evaluated an intervention that targeted individuals who seek care in the retail sector by offering free community-based malaria diagnostic testing through CHWs. Following testing, those with a positive test result received a voucher to obtain first-line antimalarials at a nearby pharmacy at a heavily subsidized price. The intervention demonstrated a significant improvement in testing before treatment.

Ideally, there should be adequate demand for the commodity or opportunity, and supply should be able to meet demand. In our cluster randomized trial, despite improvements in malaria testing before treatment of fever, we still observed that only 55 percent of those with recent illness reported having a malaria test before taking drugs and 21% still purchased antimalarials over-the-counter without a test[6], representing substantial scope for further improving the reach and impact of this intervention. We predicted that the intervention could have led to higher testing rates and sought to understand whether the impact may have been limited by either demand for, or supply of, testing or both.

Here we report a secondary analysis of trial data collected at 12 and 18 months after the intiatiion of our cluster-randomised trial of community-based diagnostic testing in western Kenya[6]. We sought to understand what factors might increase or decrease demand for testing from the community, compared to factors that may have limited supply of testing by CHWs. A comparison of constraints on the demand-side and supply-side could help us understand the conditions under which the intervention operated well or was hindered by a mismatch in supply

For peer review only - http://bmjopen.bmj.com/site/about/guidelines.xhtml

and demand. The results will be instrumental for improving the design of community-based interventions delivered at scale.

Methods

Description of the main trial

This is a post-hoc analysis of data collected within intervention clusters during a large community based cluster randomised trial. The main results of the trial have been reported elsewhere (6). The purpose of the parent trial was to encourage clients to receive testing for malaria before seeking treatment. This was accomplished by training and equipping community health workers (CHWs) to perform rapid-diagnostic tests (mRDTs) for malaria and to provide clients who test positive for malaria with a voucher that allows them access to subsidized high-quality antimalarial drugs through participating pharmacies.[6] The trial was conducted in 32 community clusters in western Kenya (population approximately 160,000). Eligible clusters had retail outlets selling AL and existing community health worker (CHW) programs and were randomly assigned 1:1 to control and intervention arms. In intervention areas (comprised of 16 community units), CHWs were available in their villages to perform malaria rapid diagnostic tests (mRDTs) on demand for any individual >1 year of age experiencing a malaria-like illness. The mRDT-positive individuals received a voucher for a discount on a quality-assured AL, redeemable at a participating retail medicine outlet. In control cluster (composed of 16 community units), CHWs offered a standard package of health education, prevention, and referral services. We conducted 4 population-based surveys—at baseline, 6 months, 12 months, and 18 months—of a random sample of households with fever in the last 4 weeks to evaluate predefined, individual-level trial outcomes.

For peer review only

For peer review only - http://bmjopen.bmj.com/site/about/guidelines.xhtml

In Kenya, CHWs work on a volunteer basis and there are no formal education requirements.The ability to read and write are criteria for selection by the MOH. The role of CHWs as defined by the MOH is to promote good health through providing health education, basic treatments (such as first-aid), and referrals to health care facilities. The CHWs report to Community Health Extension Workers (CHEWs) who are employees of MOH and are trained community health nurses mandated to supervise the activities of CHWs in the community units. In intervention areas, existing CHWs were trained to carry out the intervention. 272 CHWs participated in offering testing, a median of 18 CHWs per cluster (range: 10-24).

CareStart HRP2-based malaria mRDTs were used for testing. CareStart HRP2 detects *Plasmodium falciparum* which is the main species of parasite responsible for the majority of malaria cases in Kenya.[10] In addition to malaria mRDT, the study team provided CHWs with the necessary supplies to conduct the mRDT testing. CHWs were free to perform tests in the location(s) of their choice (e.g., in their own homes, at clients' homes, etc.), as long as the location allowed for proper and sufficiently private performance of the procedure.

The CHWs tested febrile patients at community level and issued AL vouchers to patients with positive results. This CHW model differs from other settings implementing community case management of malaria where the CHW is permitted to both perform the mRDT and provide antimalarials directly to the client. The voucher redemption period was three days where by after that the Medicine shops were not allowed to collect the voucher. Patients with positive malaria results bought AL from medicine shops by redeeming their vouchers and leaving the voucher at the shop. The study team collected the redeemed vouchers from the medicine shops on a bi-weekly basis and reimbursed the difference in costs.

For peer review only

For peer review only - http://bmjopen.bmj.com/site/about/guidelines.xhtml

Community surveys

A community-based survey was conducted to collect individual-level study outcomes based on a population-based survey sampling strategy. This was a repeated cross-sectional household survey at 4 time points: baseline (pre-intervention), 6 months, 12 months, and 18 months post baseline. The survey was conducted in all the 32 community units, 16 Intervention and 16 control. Households were randomly selected by systematic random sampling and one person with fever in the last four weeks was interviewed in any household. If more than one eligible fever was found in a household, one participant would be selected based on alphabetical order of the given names. Details of community surveys sample size calculation are described elsewhere.[11] Briefly, the target sample size of eligible individuals with a fever in the last 4 weeks was 640 per arm (i.e., 40 per CU) at each time point, which—assuming 22.2% of households would have an eligible member—would require contacting 5,766 households. The starting point (index house) and sampling interval were different in each survey round in order to minimize repeat surveys across multiple rounds. We restrict our analysis here to the 12 and 18 month surveys in order to evaluate the effects of interest in the time-period when the intervention effect could be measured.[6]

Basic demographic information about the participants and the households were collected. Socioeconomic scores were calculated from an asset index and then grouped into quintiles as described previously. Euclidean distance from a household to the nearest health facility was calculated from GPS coordinates. In the final survey, participants beliefs about their illness, malaria risk, diagnostic testing, and treatment were elicited by asking them about the severity of their illness, their confidence in positive test result and a negative test result, and how certain they were that they would recover from malaria after treatment with AL. “Perceived prevalence” was defined as the proportion of fevers that the respondent believed would be due to malaria among

For peer review only - http://bmjopen.bmj.com/site/about/guidelines.xhtml

10 people with fever from their village. High perceived prevalence was assigned to those who responded 8-10 out of 10, medium prevalence to those who responded 4-7 out of 10 and low to those who responded 0-3 out of 10. The survey tool can be found in supplemental materials.

Midpoint CHW survey

As part of the process evaluation, at the midpoint of this study, interviews were conducted with a random sample of 70 CHWs (median of 4 per CU) who were trained to conduct mRDTs (intervention CUs). The primary objective of these interviews were to determine the CHWs' satisfaction with their role in the malaria project and the feasibility of their continued involvement. Secondary objectives included eliciting the main challenges the CHWs have faced in implementing the study, determining unanticipated burdens on the CHWs' other activities (including their responsibilities as CHWs outside of the study as well as their non-CHW responsibilities) and identifying changes in the types of services they provide and the recipients of their services. [12]

Likert-scale questions were also asked, in which the interviewers read a statement aloud and the CHWs were asked to respond how much they agreed with the statement on a five-point scale ("strongly agree", "somewhat agree," "neutral," "somewhat disagree," "strongly disagree"). For this analysis, several variables were derived from the survey data. The client score was constructed as a composite score based on four likert scale questions – whether CHWs agreed that; clients trust the results of a positive mRDT by the CHW, they trust a negative mRDT, they follow the CHWs advice if the test was positive, they follow advice if the test was negative. A response of 'strongly agree'=2, somewhat agree='1' and neutral/somewhat disagree/strongly, disagree=0. If the sum of these four responses was 6 or greater, the score was 'high'. If less than 6, the score was 'low'. The survey tool can be found in supplemental materials.

For peer review only - http://bmjopen.bmj.com/site/about/guidelines.xhtml

These same CHWs also performed an mRDT under observation of the study supervision team and were scored on each step using a 20-step checklist that classified steps as relating to safety, accuracy, and treatment. The checklist was developed by the study team based on previous research studies to guide training and support supervision. Those who completed at least 17 of 20 steps correctly had a 'high' score for mRDT performance and <17 had a 'low' score.

Demand and supply

To investigate factors that might have influenced the success of the intervention, the analysis was divided into demand and supply factors. On the demand-side, the analysis investigates factors that might influence the community member's outcome of having a test, first from any source and then from the CHW. Only respondents with complete data on the variables of interest were included. On the supply-side, the analysis investigates the factors associated with the testing output (i.e. supply) at the CHW level, measured as the monthly number of tests conducted by each CHW. These factors include; socio-demographic characteristics of the CHW, number of households under the CHWs care, years of experience of the CHW, the number of other activities the CHW engages in under their role as a CHW (under the assumption that an increasing number of activites may compete for their time, the CHW provided a list of activities and these were summed together), the perception of the intervention by the CHW, the skill of the CHW in conducting mRDTs and the overall prevalence of malaria in their community.

Outcome measures

The primary outcome is the uptake of testing in the intervention clusters, defined as the proportion of fevers in the previous 4 weeks that received a diagnostic test from any source. The second

For peer review only - http://bmjopen.bmj.com/site/about/guidelines.xhtml

primary outcome is the uptake of testing from a community health worker among those who received a diagnostic test from any source.

The secondary outcome was the testing volume, defined as the mean monthly number of tests performed by each CHW. Monthly number of tests per CHW was available from program data collected at monthly supervision visits.

Statistical analysis

A mixed-effects logistic regression analysis was used to evaluate the primary outcome measures on the uptake of testing and investigate risk factors that might have contributed to the outcomes of the intervention reported previously.[6] We adopt a mixed effects logistic regression to account for clustering at the level of the CU . The outcomes were evaluated only for the CUs in the intervention arm and summaries provided.

For the primary outcome measures to evaluate the uptake of testing overall and from a CHW, two logistic models were generated; the first model included all individuals who reported fever in the last 4 weeks (ie all eligible participants enrolled in the survey). The second model includes a subset of those in the first model, specifically those who were tested at any source. For both models, we accounted for clustering within the CU in the univariate analysis and we accounted for clustering, and individual-level covariates like respondent's age, gender, level of educational, occupation, socio-economic status, geographic proximity to health care provider, the time they waited after falling ill before they sought a test, the level of knowledge and opinions about malaria and malaria diagnosis in the multivariate analysis.

The logistic regression model is as shown below:

For peer review only - http://bmjopen.bmj.com/site/about/guidelines.xhtml

$$log\left(\frac{p_{ij}}{1-p_{ij}}\right) = \beta_0 + \beta_1 X_{1j} + \ldots + \beta_m X_{mj} + u_j$$

where p_{ij} is the probability of the outcome for each individual i in cluster j, β_0 is the model intercept, β_{1-m} are the coefficients for each potential risk factor X_{1-m}, and u_j represents the random effects for cluster j. The structures for both models are the same, however, for model 1, any test=1 and no test=0 and for or model 2, tested at CHW=1 and tested elsewhere=0.

Fixed effects are measured using an Odds Ratio (OR) and included the 95% confidence intervals. Independent categorical variables were screened for multicollinearity with a Cramér's V statistic. In case two variables showed signs of correlation (V > 0.7), the variable least associated with the response variable was omitted.[13]

For the secondary outcome measure of CHW testing, a mixed-effects linear regression model was fitted to account for clustering at the CU. This model investigates the outcome of mean RDTs performed each month by the specific CHW and includes potential CHW-level covariates like CHW age, marital status, education level, number of households for which they were responsible, previous training on malaria case management or mRDTs, years of experience as a CHW, and formal employment status.

The linear regression model is as shown below:

$$Y_{ij} = \beta_0 + \beta_1 X_{1j} + \ldots + \beta_m X_{mj} + u_j + \varepsilon_{ij}$$

where Y_{ij} is the outcome for each individual i in cluster j, β_0 is the model intercept, β_{1-m} are the co-efficients for each potential risk factor X_{1-m}, u_j represents the random effects for cluster j, and ε_{ij} is the error term.

For peer review only - http://bmjopen.bmj.com/site/about/guidelines.xhtml

All statistical analysis was done in STATA version 15 (StataCorp).

Patient and public involvement

No patients involved

For peer review only

For peer review only - http://bmjopen.bmj.com/site/about/guidelines.xhtml

Results

A total of 272 CHWs in 16 Intervention CUs were trained to perform mRDTs and 36 retail shops were enrolled to redeem vouchers. The intervention was launched on July 21, August 17, and September 22, 2015 in Bokoli, Kiminini, and Ndivisi sub-counties western Kenya, respectively and continued until May 5, 2017.

Demand-side factors for use of malaria diagnostic testing

In total, 3719 households were approached, 1877 had an eligible fever in the last four weeks, 56 did not know if there was an eligible fever, 0 refused and 1738 completed the survey with no missing answers (92.6%). The community-based demand analysis relies on 1738 complete surveys from participants in randomly selected households in intervention clusters with an acute illness in the last four weeks (**Table 1**). Community members were interviewed at 12 or 18 months (47.2% and 52.8% respectively) following the initiation of the intervention. 55% (n= 948) of participants reported having a malaria test for their recent illness while 38.4% (n=364/948) of those tested reported having had the test at CHW. Among all surveyed participants, 41% (n= 710) were male and 59% (n= 1028) were female. 21.2% (n= 368) of the recent fevers were aged <5 years, 43.3% (n= 752) were 5 -17 years while 36% (n= 618) were 18> years. Only 21.4% (n= 371) of the participants had completed their secondary education while 45% (n= 778) had completed their primary education. Overall 42.8% lived more than 2km from their nearest public health facility.

Demand-side factors that were associated with uptake of malaria testing for their most recent illness are examined (**Table 1**). Respondents who delayed seeking care until the day after they fell ill had a higher odds of receiving a test than those who sought care on the same day, but there

For peer review only - http://bmjopen.bmj.com/site/about/guidelines.xhtml

was no difference between those who sought care the same day and those who waited until the second day. Being aware of your local CHW increased the odds of having a test from any source, facility or CHW (Adj. OR=1.50, 95% CI: 1.10-2.04). Respondents from the wealthiest households were 53% more likely to have a test compared to the least wealthy households (Adj. OR=1.53 95%CI: 1.14-2.06). Being an adult at least 18 years old was negatively associated to taking a test (Adj. OR=0.59, 95% CI:0.43-0.82). Distance to a health facility greater than 2km (Adj. OR=0.86, 95% CI:0.67-0.11) and use of ITN (Adj. OR=1.27, 95%CI:0.93-1.74) were not significantly associated with testing uptake.

Table 1. Participant characteristics at the 12 and 18 month survey and association between participant characteristics and malaria diagnostic testing. Adjusted and unadjusted odds ratios for receiving a malaria diagnostic test, either microscopy or mRDT, from any source for the most recent febrile illness.

	Sample characteristics	Univariate analysis		Multivariate analysis	
	(N=1738)	Unadjusted Odds Ratio (95%CI)	P-value	Adjusted Odds Ratio (95%CI)	P-value
Outcome=tested by any source	948/1738 (54.6%)				
Time to seek care (*Reference=same day*)	775 (44.4%)				
Next day	559 (32.3%)	**1.21[1.05,1.42]**	**0.01**	**1.23[1.06,1.43]**	**0.01**
The second day	223 (12.8%)	1.01[0.78,1.32]	0.94	1.13[0.86,1.48]	0.38
More than two days	182 (10.5%)	0.94[0.73,1.22]	0.66	1.12[0.87,1.44]	0.37
Use an ITN *(Reference=does not use an ITN)*	1387 (79.8%)	1.37[0.99,1.92]	0.06	1.27[0.93,1.74]	0.13
Distance to public health facility >2km (*Reference = <2km*)	837 (48.2%)	0.83[0.64,1.06]	0.14	0.86[0.67,1.11]	0.26

For peer review only - http://bmjopen.bmj.com/site/about/guidelines.xhtml

Aware of a CHW in their village *(Reference=is not aware of a CHW in their village)*	1424 (81.9)	**1.43[1.01,2.05]**	**0.04**	**1.50[1.10,2.04]**	**0.01**
Patient age (*Reference=1-4y*)	368 (21.1%)			-	-
5 to 17y	752 (43.3%)	0.76[0.54,1.08]	0.12	0.79[0.56,1.12]	0.18
18+y	618 (35.6%)	**0.59[0.43,0.81]**	**<0.01**	**0.59[0.43,0.82]**	**<0.01**
Patient is male (*Reference = female*)	710 (40.9%)	1.02[0.86,1.23]	0.78	0.94[0.80,1.10]	0.43
Respondent education level (*Reference=not completed primary*)	589 (33.9%)			-	-
Completed primary	778 (44.8%)	1.12[0.93,1.36]	0.23	1.08[0.87,1.33]	0.48
Completed secondary	371 (21.3%)	**1.41[1.04,1.90]**	**0.03**	1.25[0.93,1.70]	0.14
Wealth quintile (*Reference=0-20th*)	466 (26.8%)			-	-
20 - 40th	222 (12.8%)	1.04[0.68,1.62]	0.83	1.02[0.65,1.59]	0.94
40 - 60th	365 (21.0%)	1.14[0.86,1.50]	0.36	1.08[0.82,1.43]	0.59
60 - 80th	355 (20.4%)	1.10[0.79,1.53]	0.56	1.03[0.73,1.46]	0.86
>80th	330 (19.0%)	**1.59[1.15,2.21]**	**<0.01**	**1.53[1.14,2.06]**	**<0.01**

* This table refers to those who reported getting a test from any source

The analysis further focused on those who were tested for malaria by a CHW **(Table 2).** Out of 948 participants who reported having a test, 38.4% reported having taken a test from a CHW (364/948). In 76% of the cases tested by the CHW, the participant reported contacting the CHW as the first (n=277/364) action taken for illness while 11% reported taking drugs at home and then contacting the CHW. It is noted that wealthier participants prefer being tested at the health facility compared to the least wealthy households (Adj. OR for testing at a CHW=0.32, 95%CI:0.17-0.60), and those who completed secondary school have lower odds of taking a test

For peer review only - http://bmjopen.bmj.com/site/about/guidelines.xhtml

at the CHW although this comparison did not reach statistical significance in the adjusted model (Adj. OR=0.68, 95%CI:0.44-1.07). On the other hand, school-aged children between 5-17 years were more than twice as likely to be tested by a CHW compared to those below 5 years old (Adj. OR=2.39, 95%CI: 1.43-4.01).

Table 2: Association between patient characteristics and having an mRDT from a CHW before taking any treatment. In this analysis, awareness of a CHW in the village was not included as a covariate because 100% of individuals who had a test by a CHW were also aware of the CHW in the village. Of note, 73.5% of those tested elsewhere were also aware of a CHW in their village.

	Univariate analysis		Multivariate analysis	
Participant Characteristics of those who took a test from CHW (n=948)	Unadjusted Odds Ratio (95%CI)	P-value	Adjusted Odds Ratio (95%CI)	P-value
Time to malaria test (*Reference=same day*)			-	-
Next day	0.96[0.71,1.31]	0.80	0.96[0.69,1.33]	0.81
After two days	0.73[0.44,1.21]	0.22	0.68[0.42,1.10]	0.12
More than two days	0.81[0.49,1.34]	0.41	0.87[0.50,1.52]	0.62
Use an ITN *(Reference=does not use an ITN)*	**0.57[0.41,0.78]**	**<0.01**	0.77[0.55,1.06]	0.11
Distance to public health facility>2km (*Reference = <2km*)	1.32[0.80,2.20]	0.28	1.29[0.82,2.03]	0.27
Patient age (*Reference=1-4y*)			-	-
5 to 17y	**2.43[1.49,3.95]**	**<0.01**	**2.39[1.43,4.01]**	**<0.01**
18+y	0.98[0.67,1.43]	0.91	1.09[0.72,1.66]	0.68
Patient is male (*Reference = female*)	1.23[0.98,1.54]	0.07	1.02[0.82,1.26]	0.85
Respondent education level (*Reference=none*)			-	-
Completed primary	0.86[0.69,1.09]	0.22	0.96[0.75,1.22]	0.74
Completed secondary	**0.45[0.29,0.68]**	**<0.01**	0.68[0.44,1.07]	0.10

For peer review only - http://bmjopen.bmj.com/site/about/guidelines.xhtml

Wealth quintile (*Reference=0-20th*)			-	-
20 - 40th	1.13[0.65,1.94]	0.671	1.11[0.63,1.96]	0.72
40 - 60th	0.83[0.63,1.09]	0.175	0.79[0.58,1.05]	0.11
60 - 80th	**0.67[0.47,0.96]**	**0.028**	**0.66[0.45,0.97]**	**0.03**
>80th	**0.26[0.13,0.53]**	**<0.001**	**0.32[0.17,0.62]**	**<0.01**

* This table refers to those who reported getting a test from a CHW

To understand how individual perceptions about malaria testing and treatment, malaria risk, and illness severity affected uptake of testing or testing at a CHW, the analysis focused on the final survey at 18 months survey **(Table 3)**. After adjusting for demographic characteristics, patient perceptions were differentially related to testing overall and testing specifically with a CHW. Both confidence in AL treatment (Adj. OR=2.75, 95% CI: .1.54-4.92) and confidence in the accuracy of an mRDT performed by a CHW (Adj. OR=2.43, 95% CI: 1.12-5.27) were strongly positively associated with testing at a CHW. Those who reported their illness as severe were more likely to be tested (Adj. OR=2.37, 95% CI: 1.58-3.58) but had a lower odd of testing at a CHW (Adj. OR=0.44, 95% CI: 0.22-0.87). Confidence in an mRDT result, either positive or negative was not significantly associated with receiving a test for a recent illness, but those who reported a high confidence in a negative mRDT result had substantially higher odds of testing with a CHW than in a facility, although this did not reach statistical significance at the 95% level (Adj OR=1.58, 95%CI: 0.89-2.83). Finally, those who reported a moderate perceived prevalence in their village (ie 4-7 fevers out of 10 would be due to malaria) or high perceived prevalence (ie 8-10 out of 10 fevers would be due to malaria) had increasingly higher odds of receiving a test from a CHW (medium prevalence Adj. OR=2.52, 95%CI: 1.26-5.06, high prevalence Adj. OR=2.70, 95% CI: 1.17-6.22).

For peer review only - http://bmjopen.bmj.com/site/about/guidelines.xhtml

Table 3: Relationship between participant beliefs and perceptions and uptake of testing from any source or from a CHW. Models are adjusted for patient age, gender, household wealth quintile and the education level of the respondent in addition to the variables in the table.

	Univariate analysis		Multivariate analysis	
**Had a malaria test (N=703)**	Unadjusted Odds Ratio (95%CI)	P-value	Adjusted Odds Ratio (95%CI)	P-value
Confidence in a positive mRDT result	1.02[0.67,1.54]	0.93	0.87[0.53,1.41]	0.57
Confidence in a negative mRDT result	1.34[0.87,2.03]	0.19	1.24[0.72,2.14]	0.43
Confidence in AL treatment	1.27[0.94,1.71]	0.12	1.06[0.81,1.40]	0.63
Trust an mRDT by a CHW as much as one at a facility	0.85[0.55,1.32]	0.48	**0.66[0.44,0.97]**	**0.03**
Illness is severe	**2.36[1.64,3.39]**	**<0.001**	**2.37[1.58,3.58]**	**<0.001**
Prevalence (*Reference= low*)			-	-
Medium	1.51[0.89,2.57]	0.13	1.48[0.85,2.58]	0.16
High	1.12[0.70,1.80]	0.64	1.05[0.63,1.77]	0.85
	Univariate analysis		Multivariate analysis	
**Had a test with a CHW (N=428)**	Unadjusted Odds Ratio (95%CI)	P-value	Adjusted Odds Ratio (95%CI)	P-value
Confidence in a positive mRDT result	1.45[0.89,2.34]	0.13	1.04[0.64,1.71]	0.86
Confidence in a negative mRDT result	1.61[0.99,2.64]	0.06	1.58[0.89,2.83]	0.12
Confidence in AL treatment	**2.67[1.52,4.70]**	**<0.01**	**2.75[1.54,4.92]**	**<0.01**
Trust an mRDT by a CHW as much as one at a facility	**2.91[1.29,6.58]**	**0.01**	**2.43[1.12,5.27]**	**0.03**
Illness is severe	0.60[0.35,1.03]	0.06	**0.44[0.22,0.87]**	**0.01**
Prevalence (*Reference= low*)			-	-
Medium	**2.87[1.48,5.56]**	**<0.01**	**2.52[1.26,5.06]**	**0.01**
High	**2.85[1.31,6.23]**	**0.01**	**2.70[1.17,6.22]**	**0.02**
Confidence in a positive mRDT result	1.45[0.89,2.34]	0.13	1.04[0.64,1.71]	0.86

For peer review only - http://bmjopen.bmj.com/site/about/guidelines.xhtml

Diagnostic testing by CHWs

In total, 32,404 mRDTs were conducted by the CHWs over the intervention period and 33.7% (n= 10,870) percent were positive. All mRDT interpretations were counterchecked by the study team. Those with a positive test received a voucher for a discounted quality-assured AL. These vouchers were redeemed at a participating outlet by 93.9% of voucher recipients. There was no instances of stock out of mRDTs among our CHWs. To explored factors that may be associated with the CHWs ability to conduct mRDTs, we examined the association between testing volume, defined as the mean number of mRDTs conducted per month, and the different CHW demographic characteristics (**Table 4**). Of the 244 trained CHWs with complete data, 147 (60.3%) were above 40 years of age, 72 (29.5%) were male. There were 107 (43.8%) CHWs who had not completed secondary education.

Table 4: Association between mean number of tests performed per month and CHW characteristics. 272 CHWs were trained, of which 28 were missing one or more of marital status, employment status, previous RDT training, or education level. The 244 with complete data are reported here.

Mean number of tests per month	4.60 (SD=3.07)	**Univariate Analysis**		**Multivariate Analysis**	
		**N = 244**		**N = 244**	
CHW characteristics	n (sample proportion)	Coeff (95%CI)	P-value	Coeff (95%CI)	P-value
Male	72 (0.295)	-0.30 [-1.28,0.68]	0.52	-0.28 [-1.24,0.68]	0.54
Age<br><40 years *(Reference)*	97 (0.397)	-	-	-	-
> = 40 years	147 (0.603)	-0.21 [-1.06,0.63]	0.60	-0.42 [-1.12,0.28]	0.23
Married *(Reference = unmarried/divorced)*	202 (0.828)	0.37 [-0.57,1.30]	0.42	0.22 [-0.92,1.35]	0.69
Education	107 (0.438)	-	-	-	-

For peer review only - http://bmjopen.bmj.com/site/about/guidelines.xhtml

Below secondary (*Reference)*					
Completed Secondary	137 (0.562)	0.45 [-0.33,1.23]	0.24	0.23 [-0.40,0.87]	0.44
Formal Employment *(Reference = no formal employment)*	33 (0.135)	**1.13 [0.01,2.28]**	**0.05**	**1.37 [0.05,2.70]**	**0.04**
Previously trained in mRDT	46 (0.188)	-0.46 [-1.64,0.72]	0.42	**-1.40 [-2.44, -0.37]**	**0.01**
Households[1] <50 *(Reference)*	134 (0.549)	-	-	-	-
50-100	66 (0.271)	**2.14[0.63,3.64]**	**<0.01**	**1.73[0.70,2.74]**	**<0.01**
>100	44 (0.180)	1.18[-0.40,2.76]	0.13	**1.49[0.74,2.24]**	**<0.01**
Cluster-level test positivity[2] Low (≦25%; *Reference)*	141 (0.588)	-	-	**-**	**-**
High (>25%)	103 (0.422)	**2.54[0.97,4.10]**	**<0.01**	**2.14[1.05,3.22]**	**<0.01**

[1] Number of households for which the CHW reported being responsible

[2] Percentage of mRDTs conducted by the CHWs in that cluster which turned positive over the study period.

In a multivariate analysis, formal employment, previous mRDT training, number of households and mRDT-positivity rate at the cluster-level were associated with volume of tests done. Sex, age, being married and education level were not significantly associated with testing volume. CHWs who were formally employed in some other capacity alongside their CHW voluntary work performed on average 1.37 more tests per month (Adj coefficient=1.37, 95% CI: CI: 0.05-2.70) but those who reported being trained previously on mRDTs performed 1.4 fewer tests per month (Adj coefficient=1.4, 95%CI: -2.44-0.37). CHWs serving areas with a high proportion of positive tests (proportion mRDT +ve >25%) tested on average 2 more clients per month than those in lower prevalence areas (Adj coefficient= 2.14, 95%CI: 1.05-3.22). CHWs who were responsible for at least 50 households tested more clients (Adj coefficient=1.73, 95%CI: 0.702.74), although this did not increase further with when the number of households exceeded 100 (Adj coefficient = 1.49, 95%CI: 0.74-2.24).

For peer review only - http://bmjopen.bmj.com/site/about/guidelines.xhtml

A univariate and multivariate analyses to test the association between testing volume and CHW perceptions about their role at the midpoint survey (**Table 5**). High client score was significantly associated with higher testing volume performed by CHWs who perceived that their clients trusted their tests (Adj. coefficient=1.37, 95%CI: 0.11-2.62). The CHWs competency at conducting all 20 steps of the mRDT process (mRDT score), the number of different activities to which CHWs were committed, whether they named mRDT testing as their most important activity, or whether they cited extrinsic motivation (money, airtime, or other) as important reasons for continuing their work were all not significantly associated with average tests performed per month.

Table 5: Association between mean number of tests per month and CHW perceptions about their role at the midpoint survey. Multivariate odds ratios are adjusted for age, gender, education level, formal employment. The analysis includes a random sample of CHWs selected for midpoint evaluation.

	n (sample proportion)	**Univariate Analysis**		**Multivariate Analysis[1]**	
CHW characteristics	N=70	Coeff (95%CI)	P-value	Coeff (95%CI)	P-value
mRDT Score[2] (*Reference=Low*)	-	-	-	-	-
High	54 (0.771)	0.19[-1.25,1.64]	0.78	0.11[-1.34,1.57]	0.87
Number of different activities last month (*Reference=<=3*)	-	-	-	-	-
> 3 activities	19 (0.271)	0.35[-1.56,2.26]	0.70	-0.45[-2.04,1.15]	0.56
Most important activity is malaria testing[3]	48 (0.686)	0.88[-0.25,2.01]	0.12	0.07[-1.16,1.32]	0.90
Cited extrinsic motivators as important[4]	28 (0.400)	0.33[-1.52,2.17]	0.71	0.67[-0.92,2.25]	0.38
Client Score[5] (*Reference= Low*)	-	-	-	-	-
High	48 (0.686)	**1.46[0.10,2.83]**	**0.04**	**1.37[0.11,2.62]**	**0.04**
Years of experience as a CHW (*Reference = <=5y*)	-	-	-	-	-
>5 years	38 (0.543)	-0.35[-2.68,1.98]	0.75	-0.51[-2.56,1.55]	0.61

For peer review only - http://bmjopen.bmj.com/site/about/guidelines.xhtml

[1] Multivariable models are also adjusted for CHW demographic factors (age, gender, education, employment, and cluster-level prevalence)
[2]mRDT score - CHW scored > 17 correct steps on a 20-step checklist for mRDT preparation and interpretation conducted 6 months after training
[3]Equals one if malaria testing was cited as their most important activity or 0 if they cited any other activity as most important

[4] Equals 1 if the CHW cited money, or 0 if the CHW only cited non-monetary incentives or desire for recognition as important motivators for their role as a CHW
[5] Client score was derived from four questions where the CHW reported how confident clients were in the testing they provided.

Discussion

This study adds to the evidence that it is feasible for CHWs to offer malaria testing using mRDTs in the community. With the use of CHWs, large-scale malaria testing intervention can reach the underserved population who cannot afford to visit the health facilities for care and those who seek care in the retail sector. The existence of CHWs with the ability to test for malaria using mRDT provides a great opportunity, however, various factors may affect the demand and supply of testing using mRDT by CHWs.

Here we examine the outcomes of a community-based malaria testing to understand how to improve intervention design and maximize the impact of such programs. The analysis was organized around the target group (community members) and their uptake or demand for the diagnostic testing intervention, and the supply of testing by the CHWs responsible for delivering the intervention. We were able to separate the determinants of uptake of malaria diagnostic testing generally from the determinants of uptake of malaria rapid diagnostic test performed by a

For peer review only - http://bmjopen.bmj.com/site/about/guidelines.xhtml

CHW. These contrasting but complementary analyses helped us identify bottlenecks to intervention coverage at the community level.

Analysis of community survey data revealed that increased demand for malaria diagnostic testing before treating a fever was lower among adults and is positively associated with wealth, findings which are consistent with other published studies.[14-17] We also find that being aware of the local CHW increased demand for testing from any source. This is similar to other studies that have evaluated the contribution of CHWs to population health-seeking behavior.[16,18] In the context of our program, this is especially important because the testing was most often initiated by the community member seeking out a CHW rather than a door-to-door campaign initiated by the CHW. Malaria testing by a CHW provides an opportunity for reducing health disparities by ensuring the poor and underserved population get access to testing and are able to make an informed choice on how to manage their illness. Our results show that community members with a lower socioeconomic score were more likely to receive a test from a CHW. Malaria testing by the CHW was free and this finding therefore aligns well with the intention of the intervention. Testing by a CHW was also more common among those with high confidence in the reliability of a test done by a CHW and also among those with high confidence in artemether-lumefantrine, the treatment offered through the CHW testing program. Taken together, these results suggest that the community awareness of and perception of the CHW is key in improving demand. In contrast, although testing was high among those who considered their illness to be very severe, these individuals were less likely to be tested by a CHW. This result is appropriate and consistent with the goals of the program which included immediate referral of more severe cases.

From the perspective of CHWs delivering malaria testing, a strong association was not observed between demographic characteristics and testing volume, but we did note that CHWs with

For peer review only - http://bmjopen.bmj.com/site/about/guidelines.xhtml

concurrent formal employment tested more patients on average than those without which could indicate greater visibility or mobility in the community. It was also observed that the monthly testing volume increased when the number of households under a CHWs purview exceeded 50, but we could not detect a significant increase beyond that when households exceeded 100, suggesting a threshold effect whereby more households created more demand for testing but the ability to meet the demand may be saturated after some threshold beyond 100 households. Finally, we report that testing rates were higher in clusters with high mRDT-positivity rates, possible reflecting a greater awareness of malarial illness or higher demand for malaria services in areas with higher malaria burden. This could also reflect higher confidence in a test among CHWs or communities members who have experience with some tests turning positive as has been seen in the healthcare setting. [19]

A deeper analysis of CHW perception of the intervention was undertaken in a smaller random sample of participating CHWs. We show that testing volume is not affected by competing CHW-related priorities, but is associated with how they perceive their clients' confidence in testing and the likelihood of the client accepting the mRDT result and advice. This aligns well with the picture from the demand-side where we observe that confidence in an mRDT performed by a CHW is strongly associated to testing with a CHW.

This study had several limitations. First, we are not able to estimate the effect of free testing on the uptake of testing. Considering the study utilized diagnosis dependent subsidies to test the rational use of AL, a similar study that deploys free testing in the absence of a conditional voucher would be required to resolve this. Similarly, it could be important to compare the effect of an intervention that delivered both the test and the treatment through the CHW rather than using a voucher redeemable in the private sector. Second, although the survey data has provided

For peer review only - http://bmjopen.bmj.com/site/about/guidelines.xhtml

valuable insight on understanding drivers to demand and supply of malaria testing, seeking provider (CHW) and patient perspectives in a qualitative framework could provide a richer understanding of the facilitating factors and barriers to scaling up such an intervention, particularly the ways in which preferences and changes in behavior may influence uptake of the intervention. Finally, though our survey design used repeated cross-sectional sampling, it is possible that the same household and/or febrile individual was surveyed at multiple survey time points and any resulting association was not explicitly modeled in our regression analysis.

Conclusion

In summary, scale up of health interventions through CHWs is feasible and effective at reaching the poorest households. However, in order to maximize the impact of community-based interventions, it is important to recognize factors that may restrict both delivery and demand for such services. It is critical to not only create awareness of CHW services but foster trust in both the CHWs ability to deliver services and the health intervention itself. Engendering confidence in the CHWs proficiency is likely to have a synergistic effect on both demand for services and commitment to delivering services.

Declarations

Ethics approval and consent to participate: Ethical approval was granted by Moi University Institutional Research and Ethics Committee (*Formal Approval No. 0001403*) and Duke University Institutional Review Board (*Pro00063384*). Written informed consent was obtained from participants during household interviews and from CHWs prior to collecting midpoint evaluation

For peer review only - http://bmjopen.bmj.com/site/about/guidelines.xhtml

data. All study methods were carried out in accordance with ethical guidelines and regulations set out by both Moi University and Duke Ethical Review Boards.

Consent to publish: Not applicable

Data availability statement: The datasets used and/or analysed during the current study are available from the corresponding author on reasonable request.

Competing interests: The authors declare that they have no competing interests

Funding: This study was supported from the National Institute of Allergy and Infectious Diseases of the National Institutes of Health (US) (grant number R01AI110478). The funders had no role in study design, data collection and analysis, decision to publish, or preparation of this manuscript. The content is solely the responsibility of the authors and does not necessarily represent the official views of the National Institute of Allergy and Infectious Diseases or the National Institutes of Health.

Contributors:

Joseph Kipkoech – Conceptualization, writing original draft, revision and editing final draft

Josephine Malinga – Data curation, writing original draft, revision and editing final draft

Edna Sang - writing original draft, writing original draft, revision and editing final draft

George Ambani – Data collection, writing original draft, revision and editing final draft

Lucy Abel - Data collection, writing original draft, revision and editing final draft

Erick Nalianya - Data collection, writing original draft, revision and editing final draft

For peer review only - http://bmjopen.bmj.com/site/about/guidelines.xhtml

Jane Namae - Data collection, writing original draft, revision and editing final draft

Matthew Boyce - Review and editing final draft

Jeremiah Laktabai – Conceptualization, review and editing final draft

Diana Menya - Conceptualization, review and editing final draft

Wendy Prudhomme-O'Meara – Conceptualization, funding acquisition, writing original draft, revision and editing final draft

Acknowledgements: This study could not have been carried out without the exceptional attention to detail and commitment of our field team that include: I. Khaoya, L. Marango, E. Mukeli, L. Nukewa, E. Wamalwa, and A. Wekesa. We are grateful to the study participants and the community health workers as well as the local community leadership that supported this work. We would also like to acknowledge the partnership and support of our Health Management Team colleagues in each subcounty and in particular V. Kiplagat, A. Kyalo, S. Malenya, and P. Musita.

Supplemental material

Supplemental file 1: Community survey tool used to collect information about recent fevers among participating households

Supplemental file 2: Survey tool used at the midpoint CHW evaluation.

For peer review only

References

1. World Health Organization. World malaria report 2020: 20 years of global progress and challenges. Geneva; 2020.

2. World Health Organization, Global Malaria Programme. Guidelines for the treatment of malaria. Geneva;. 2015. Available from: http://www.ncbi.nlm.nih.gov/books/NBK294440/ (accessed Jan 20, 2021)

3. World Health Organization. World malaria report 2019. Geneva; 2019.

4. WHO Guidelines for malaria. Geneva: World Health Organization; 2021. Available from: https://www.ncbi.nlm.nih.gov/books/NBK568494/

5. ACTwatch Group, Musuva A, Ejersa W, Kiptui R, Memusi D, Abwao E. The malaria testing and treatment landscape in Kenya: results from a nationally representative survey among the public and private sector in 2016. Malar J. 2017 21;16(1):494.

6. Prudhomme O'Meara W, Menya D, Laktabai J, Platt A, Saran I, Maffioli E, et al. Improving rational use of ACTs through diagnosis-dependent subsidies: Evidence from a cluster-randomised controlled trial in western Kenya. PLoS Med. 2018;15(7):e1002607.

7. Cohen J, Dupas P, Schaner S. Price Subsidies, Diagnostic Tests, and Targeting of Malaria Treatment: Evidence from a Randomised Controlled Trial. Am Econ Rev. 2015 Feb;105(2):609–45.

8. Briggs MA, Kalolella A, Bruxvoort K, Wiegand R, Lopez G, Festo C, et al. Prevalence of Malaria Parasitemia and Purchase of Artemisinin-Based Combination Therapies (ACTs)

For peer review only

among Drug Shop Clients in Two Regions in Tanzania with ACT Subsidies. PLOS ONE. 2014 Apr 14;9(4):e94074.

9. Mbonye AK, Lal S, Cundill B, Hansen KS, Clarke S, Magnussen P. Treatment of fevers prior to introducing rapid diagnostic tests for malaria in registered drug shops in Uganda. Malar J. 2013 Apr 16;12(1):131.

10. World Health Organization, Special Programme for Research and Training in Tropical Diseases, Foundation for Innovative New Diagnostics, Centers for Disease Control (U.S.). Malaria rapid diagnostic test performance: results of WHO product testing of malaria mRDTs : 4 (2012). [Internet]. Geneva, Switzerland: World Health Organization on behalf of the Special Programme for Research and Training in Tropical Diseases; 2012 [cited 2021 Jun 7]. Available from: http://apps.who.int/iris/bitstream/10665/77748/1/9789241504720_eng.pdf

11. Laktabai J, Lesser A, Platt A, Maffioli E, Mohanan M, Menya D, et al. Innovative public-private partnership to target subsidised antimalarials: a study protocol for a cluster randomised controlled trial to evaluate a community intervention in Western Kenya. BMJ Open. 2017 20;7(3):e013972.

12. Winn LK, Lesser A, Menya D, Baumgartner JN, Kipkoech Kirui J, Saran I, et al. Motivation and satisfaction among community health workers administering rapid diagnostic tests for malaria in Western Kenya. J Glob Health. 2018 Jun;8(1):010401.

For peer review only

For peer review only - http://bmjopen.bmj.com/site/about/guidelines.xhtml

13. Burnham KP, Anderson DR, Huyvaert KP. AIC model selection and multimodel inference in behavioral ecology: some background, observations, and comparisons. Behav Ecol Sociobiol. 2011 Jan 1;65(1):23–35.

14. Chibwana AI, Mathanga DP, Chinkhumba J, Campbell CH. Socio-cultural predictors of health-seeking behaviour for febrile under-five children in Mwanza-Neno district, Malawi. Malar J. 2009 Sep 24;8(1):219.

15. Cohen J, Cox A, Dickens W, Maloney K, Lam F, Fink G. Determinants of malaria diagnostic uptake in the retail sector: qualitative analysis from focus groups in Uganda. Malar J. 2015 Dec;14(1):89.

16. Kisia J, Nelima F, Otieno DO, Kiilu K, Emmanuel W, Sohani S, et al. Factors associated with utilization of community health workers in improving access to malaria treatment among children in Kenya. Malar J. 2012 Jul 30;11:248.

17. Mukanga D, Tibenderana JK, Peterson S, Pariyo GW, Kiguli J, Waiswa P, et al. Access, acceptability and utilization of community health workers using diagnostics for case management of fever in Ugandan children: a cross-sectional study. Malar J. 2012 May 24;11(1):121.

18. Win Han Oo, Gold L, Moore K, Agius PA, Fowkes FJI. The impact of community-delivered models of malaria control and elimination: a systematic review. Malar J. 2019 Aug 6;18(1):269.

For peer review only

574 19 Faust C, Zelner J, Brasseur P, Vaillant M, Badiane M, Cisse M, et al. Assessing Drivers of Full Adoption of Test and Treat policy for Malaria in Senegal. Am J Trop Med Hyg. 2015 Jul 8;93(1):159–67.

577

For peer review only

For peer review only - http://bmjopen.bmj.com/site/about/guidelines.xhtml

Coupon R01 CHV Participation Assessment

April 13, 2016

Note on administration: This survey is intended to be administered orally by an enumerator to a CHV.

I. Background Information

1) Sub-county:
    1. Bungoma East
    2. Kiminini

2) Name of CU that the CHV serves: ________________________

3) CHV's gender:
    1. Male
    2. Female

4) Age: ________ Years

5) Highest school level:
    1. None
    2. Some Primary (not completed Class 8)
    3. Primary completed
    4. Primary completed and some secondary (secondary not completed)
    5. Secondary completed
    6. College
    7. University

6) For how long have you served as a CHV?
    1. < 1
    2. > 1-3 Years
    3. > 3-5 Years
    4. > 5 Years

7) Apart from serving as a CHV, what other occupation(s) or role(s) do you have?

For peer review only

1. *Write-in response:* ________________________
2. None

8) How many trainings for CHV activities of any kind have you had since the RDT training for this malaria project?
    1. None
    2. One
    3. Two
    4. Three
    5. Four
    6. Five
    7. More than five

9) How long ago was your most recent training for CHV activities of any kind?
    1. Within the past week
    2. Between one week and one month ago
    3. Between one and three months ago
    4. Between three months and 1 year ago
    5. More than one year ago

10) What was the topic of your most recent training?
    1. Malaria (this project)
    2. Malaria (another project)
    3. Maternal health
    4. Polio campaign
    5. Other vaccination program
    6. Other child health topic not mentioned above

    7. Other: _______

II. Self-description of Role

11) In the past month, what activities have you carried out as a CHV overall?
(Enumerator: check all that are mentioned; do not read the list)
    1. Malaria testing
    2. Provide vouchers for malaria drugs
    3. Mosquito net distribution
    4. Provide other malaria-related services/counseling

For peer review only

5. Polio campaign participation
6. Other vaccination campaign(s)
7. Child health services not mentioned above
8. Maternal health services not mentioned above
9. Giving health education to households
10. None
11. Other: ______________________________
____
____

12) How many times have you met with your CHEW in the past 3 months?
1. Once
2. Twice
3. Three times
4. More than three times
5. None

13) What activities do you perform specifically for the malaria project?
(Enumerator: check all that are mentioned; do not read the list)
1. Perform malaria RDTs
2. Provide vouchers for malaria drugs
3. Provide malaria drugs
4. Provide referrals
5. Provide counseling / advice
6. Other: ______________________________

14) For how many clients have you performed an RDT in the past month?
1. 1 client
2. 2-5 clients
3. 5 clients and above

15) What are the requirements for a client to receive an RDT under this malaria project?
(Enumerator: check all that are mentioned; do not read the list)
1. No requirements – anyone who asks can be tested
2. Must live in my CU
3. Has a documented fever (i.e., as measured by me)

For peer review only

4. Has a documented fever or reports a history of recent fever
5. Has a documented fever or reports a history of recent fever or reports any symptoms of malaria
6. Consent must be provided by the client (or by the parent/guardian of a minor)
7. Must not be visibly pregnant

8. Other: ___________________________________

16) What motivates you to participate in this malaria project?
1. Personal desire
2. Previous experience
3. Family
4. Organization (partner and GOK)
5. Monetary incentive (money given to group after 6 months and transport to attend supervision and supplies replenishment)
6. In-kind incentive (e.g., airtime for your phone)
7. Support from CHEWs
8. Support from link facilities in attending to clients referred for care
9. Other:____________________________

III. Satisfaction with Role

17) What are the bad things about participating in this project?
(Enumerator: check all that are mentioned; do not read the list)

(Open-ended response): ____

____

____

18) What are the good things about participating in this project?
(Enumerator: check all that are mentioned; do not read the list)

(Open-ended response): ____

____

For peer review only

Please indicate your level of agreement with the following statements with regards to the malaria project **only.**

**Question**	**Strongly Agree**	**Somewhat Agree**	**50-50**	**Somewhat Disagree**	**Strongly Disagree**
19) Overall, I am confident in my ability to perform my role in this malaria project.					
20) I receive enough supervision in my work from the malaria project study staff.					
21) I believe the clients that test positive for malaria follow the advice that I give them					
22) I believe the clients that test negative for malaria follow the advice that I give them					
23) The people in my community appreciate my work on the malaria project.					
24) I have gained more recognition in my community since I began testing for malaria.					
25) I am proud of the work that I do in the malaria project.					
26) I have gained valuable knowledge as a result of my role in this malaria project.					
27) Overall, I think the malaria project is an important activity for me to be involved in.					
28) Overall, I enjoy carrying out my role in this malaria project.					
29) I would like to continue with my role in this malaria project.					

For peer review only

30) *If the CHV answers "50-50", somewhat disagree" or "slightly disagree" to continuing their role in the project*, why?
(Enumerator: check all that are mentioned; do not read the list)

1. Requires too much time
2. Requires too much effort
3. Don't receive enough appreciation
4. Don't receive compensation
5. Frustrated that they can't provide drugs directly to clients
6. Conflicts with other CHV activities
7. Conflicts with non-CHV activities / responsibilities
8. Other ____

IV. Logistics & Challenges of Role

A. Implementation

i. Community awareness & trust of services

Question	Always aware	Usually aware	Sometimes aware	Rarely aware	Never aware
31) Of all of the clients that you provide services to for any reason, how often are the clients already aware that you provide RDT testing?					
32) Of the clients that visit you with a fever or other symptoms of malaria, how often are these clients already aware that you provide RDT testing?					
33) Of the clients that visit you with a fever or other symptoms of malaria, how often are these clients already aware that you provide vouchers for malaria drugs?					

34) How do you think your clients learn about the malaria services (testing and vouchers) that you provide?
(Enumerator: check all that are mentioned; do not read the list)

1. From me
2. From family members
3. From neighbors / friends
4. From village leaders
5. From health facility staff
6. From malaria project staff

7. Other: ____________________________

For peer review only

Please indicate your level of agreement with the following statements.

Question	Strongly Agree	Somewhat Agree	50-50	Somewhat Disagree	Strongly Disagree
35) The malaria RDT that I do can be trusted just as much as a malaria RDT done in a health facility.					
36) The clients that test positive for malaria trust the results of the test					
37) The clients that test negative for malaria trust the results of the test					
38) People I test for malaria believe I am actually testing for HIV.					

39) Do any of your clients refuse an RDT when you offer it?
1. Yes
2. No

40) *If Yes to above,* What are the reasons that you think these clients refuse?
(Enumerator: check all that are mentioned; do not read the list)
3. They don't think it's necessary
4. They don't trust me
5. They don't trust the test
6. They are already convinced they have malaria
7. They plan to buy antimalarial drugs regardless of test result
8. They don't want to be pricked
9. They think it may really be an HIV test

10. Other: ______________________

ii. Logistics & Challenges

41) What are all of the ways that clients find you to receive a malaria RDT?
(Enumerator: check all that are mentioned; do not read the list)
1. Clients visit me at my home
2. Clients visit me at a health facility

For peer review only - http://bmjopen.bmj.com/site/about/guidelines.xhtml

3. Clients visit me at church
4. I visit clients at their homes
5. Client visit me at another public location
6. Client visit me at another private location
7. Clients call my mobile phone
8. The study staff contacts me
9. The health facility contacts me

10. Other:______________________

42) Where do you provide testing for malaria? *(Enumerator: check all that are mentioned; do not read the list)*

1. At my home
2. At a health facility
3. At church
4. At client's home
5. At another public location
6. At another private location
7. At CHV Office
8. In Church
9. In Barazas (public meeting)

10. Other:______________________

43) How often do you lack all of the supplies you need to perform the malaria services of testing and providing vouchers when necessary?

1. Always
2. Sometimes
3. 50-50
4. Rarely
5. Never

44) Which, if any, supplies related to the malaria services you perform for this project have you had a stock-out of in the past month?
(Enumerator: check all that are mentioned; do not read the list)

1. RDTs
2. Buffer
3. Lancets
4. Alcohol swabs
5. Cotton wool
6. Gloves
7. Safety disposal box
8. Yellow waste bags
9. Plastic (zip-loc) bags
10. Thermometer
11. Thermometer batteries

For peer review only

12. Pen
13. Encounter forms
14. Voucher forms

15. Other:____________________________

45) How would prefer your consumption of malaria project supplies be monitored?
1. CHV counts the supplies physically
2. CHV references filled encounter forms
3. CHV uses inventory card to document consumption of supplies
4. CHV relies on the supervision team to monitor the supplies

46) What are the main challenges and barriers you face in implementing the malaria project? *(Enumerator: check all that are mentioned; do not read the list)*
1. Community is unaware that I offer malaria RDTs
2. Community is unaware that I offer vouchers for malaria drugs
3. The clients don't always trust the test results I give them
4. The clients are unhappy that I can't provide the malaria drugs to them directly
5. Transport
6. Not enough training
7. Not enough supervision from study staff
8. Not enough supervision from CHEWs
9. Lack of inventory systems to monitor supplies
10. Competing activities within the community
11. Other roles by other NGOs interfere with Malaria project
12. My other main occupation does not give me time to participate in Malaria project
13. Very few clients coming for test
14. Nature of the community not willing to interact with CHVs
15. Cultural practices e.g use of herbal medicine, seeking help from traditional healers
16. Level of education of community member (the less educated don't understand the importance of malaria test and the very educated not willing to be tested by the community health volunteer
17. Lack of mobilization at the community
18. Family relationships are affected
19. Other: ___________________________________

47) Does frequent supervision improve your ability to provide quality malaria testing?
1. Yes
2. No

48) *If yes above*, how frequently would you prefer supervision to be carried out?
1. Once a week
2. Once every two weeks
3. Once a month
4. Once every two months

For peer review only

49) What is the main purpose of supervision activities conducted by the malaria project team?
(Enumerator: check only one)
1. To monitor whether I make any mistakes
2. To provide additional training
3. To ensure data quality

50) What would help you perform your role in the malaria project better?
(Enumerator: check all that are mentioned; do not read the list)
1. More training
2. More supervision from study team
3. More supervision from CHEWs
4. More regular restocking of supplies
5. Raising community awareness about the project
6. Compensation for time spent
7. Sharing my CHV duties with another CHV
8. Other: ________________________________

iii. Time required

51) On average, how long does it take you to complete a visit with a patient complaining of malaria symptoms?

____________________ Minutes

52) In the last week, what portion of your CHV activities have been related to the malaria project?
1. Less than half
2. About half
3. More than half

53) How many hours do you think you spend on the malaria project activities in an average week (including seeing clients, filling forms, meeting with malaria study staff, etc.)?

____________________ Hours

54) How many hours do you think you spend on all of your other CHV activities and responsibilities in an average week (including seeing clients, reporting, restocking, trainings, etc.)?

____________________ Hours

55) Do you think the total time you spend on all CHV activities has decreased, increased, or stayed about the same since you began the malaria project activities?

For peer review only

1. Decreased
2. Stayed about the same
3. Increased

B. <u>Relation to other CHV activities</u>

56) Has participating in the malaria project had an effect on your other CHV activities?
    1. Yes
    2. No

57) If yes, in what ways:
    (Enumerator: check all that are mentioned; do not read the list)
    1. Improved skills applicable to other CHV activities
    2. More clients visit me for non-malaria services
    3. Less time to complete other CHV activities

    4. Other: ____________________

58) Please indicate to what extent you agree with the statement: Due to my involvement with the malaria project, I now provide more non-malaria health services than before the malaria project.
    1. Strongly agree – I provide much more non-malaria health services than before
    2. Somewhat agree
    3. Neither agree nor disagree – it has stayed the same
    4. Somewhat disagree
    5. Strongly disagree – I provide much less non-malaria health services than before

(Only ask the next 2 questions if "strongly agree" or "somewhat agree" above):

59) If "strongly agree" or "somewhat agree" above: What are the other (non-malaria) services you provide more of?
    (Enumerator: check all that are mentioned; do not read the list)
    1. Maternal health services
    2. Vaccinations
    3. Providing health information
    4. *… (Other typical CHV activities to include in this list?)*

    5. Other: ___________________________

60) To whom do you provide more non-malaria services than before the malaria project started? (select all that apply):
    (Enumerator: check all that are mentioned; do not read the list)
    1. Men
    2. Women

For peer review only

3. Children

61) Please indicate to what extent you agree with the statement: There are other CHV activities or services I would normally have performed in the last month that I did not because the malaria project took too much of my time.
   1. Strongly agree
   2. Somewhat agree
   3. 50-50
   4. Somewhat disagree
   5. Strongly disagree

62) If yes, what activities / services?
(Enumerator: check all that are mentioned; do not read the list)
   1. Maternal health services
   2. Vaccinations
   3. Providing health information
   4. *… (Other typical CHV activities to include in this list?)*

   5. Other: ___________________________

63) Please indicate to what extent you agree with the statement: There are other responsibilities outside of my work as a CHV that I would normally have performed in the last month that I did not because my work as a CHV took too much of my time.
   1. Strongly agree
   2. Somewhat agree
   3. 50-50
   4. Somewhat disagree
   5. Strongly disagree

64) *If "strongly agree" or "somewhat agree" above:* What responsibilities outside of your role as a CHV have been affected? (childcare, other volunteering, church, education/study, family, etc.)
(Enumerator: check all that are mentioned; do not read the list)
   1. Childcare
   2. Family responsibilities
   3. Other work
   4. Other volunteering / community activities
   5. Religious activities

   6. Other: ___________________________

65) What, if an, relationships have been affected by your work as a CHV on this malaria project? *(Enumerator: check all that are mentioned; do not read the list)*
   1. Children
   2. Spouse
   3. Siblings
   4. Parents
   5. Other family members not mentioned above

For peer review only

6. Neighbors
7. Friends
8. Other: ___________________________

66) What do you think are the most important services you provide as a CHV?
(Enumerator: check all that are mentioned; do not read the list)

1. Malaria testing
2. Provide vouchers for malaria drugs
3. Mosquito net distribution
4. Provide other malaria-related services/counseling
5. Polio campaign participation
6. Other vaccination campaign(s)
7. Child health services not mentioned above
8. Maternal health services not mentioned above
9. Giving health education to households
10. None
11. Other: ___________________________

____

____

67) What do you think is the most important service you provide as a CHV?
(Enumerator: Only allow one answer)

1. Malaria testing
2. Provide vouchers for malaria drugs
3. Mosquito net distribution
4. Provide other malaria-related services/counseling
5. Polio campaign participation
6. Other vaccination campaign(s)
7. Child health services not mentioned above
8. Maternal health services not mentioned above
9. Giving health education to households
10. None
11. Other: ___________________________

____

____

68) In order to monitor the quality of the tests done, some CHVs in this project were randomly chosen to use a device called a DekiReader that automatically reads the RDT cassette and displays the results. Did you use this device?

1. Yes
2. No
3. I was selected but I couldn't use it (too difficult)
4. Not sure

If 1 or 3, ask next 3 questions:

69) To what extent would you agree with the statement, "The DekiReader was easy to use correctly"?

1. Strongly agree
2. Somewhat agree
3. 50-50
4. Somewhat disagree
5. Strongly disagree

70) To what extent would you agree with the statement, "The Deki Reader helped me improve my ability to perform RDTs correctly"?
    1. Strongly agree
    2. Somewhat agree
    3. 50-50
    4. Somewhat disagree
    5. Strongly disagree

71) If you had the option to use the Deki Reader for every test as part of your routine work, would you want to have it or not?
    1. Yes
    2. No
    3. Don't Know / Unsure

72) Do you have any concerns or suggestions to improve the implementation of the malaria project?
    (Open-ended response): ____

    ____

    ____

    ____

Thank you for your participation and feedback!

For peer review only

Checklist for performing Rapid Diagnostic Testing correctly

Name of CHV ____________________ Community Unit ___________________

Name of observer____________________________ Date _____________________

No.	Task	Task Category	Correctly done No = 0 Yes =1	Observer's comments
	**A. RDT preparation**			
1.	Assembles RDT packet, buffer, swab, lancet			
2.	Read expiration date			
3.	Remove contents of test packet			
4.	Write patient's name on cassette			
	**B. Patient preparation**			
5.	Identify patient's details and record patient's name and date on the RDT cassette			
6.	Explain procedure to patient. Provide reassurance, as needed			
7.	Wear gloves	Safety		
	**C. Blood collection + dispensing**			
8.	Select 4th finger of left hand for blood collection			
9.	Clean finger with alcohol swab and allow it to dry	Safety		
10.	Prick finger firmly with sterile lancet	Safety		
11.	Discard lancet in sharps bin immediately after pricking finger. Do not set it down first	Safety		
12.	DO NOT squeeze finger excessively			
13.	Collect an adequate volume of blood with pipette	Accuracy		
14.	Dispense blood in correct well	Accuracy		
15.	Discards the pipette in the sharps box	Safety		
16.	Dispose of gloves, wrappers, alcohol swab, loop, desiccant and cassette in non-sharps container	Safety		
	**D. RDT procedure and reading results**			
17.	Dispense correct volume of buffer	Accuracy		
18.	Wait for twenty (20) minutes	Accuracy		
19.	Verify internal test control			
20.	Read results correctly	Accuracy		
	**E. Recording results**			
21.	Records date and time correctly on encounter form	Treatment		
	**F. Deliver the results**			
22.	Explain to the patient the results and what they mean	Treatment		

For peer review only

A. *Household survey instrument* (Note: the survey will be administered electronically)

1. Date of interview (DD/MM/YY):

☐☐ / ☐☐ / 20 ☐☐

2. Name of interviewer: ____________

3. Interviewer ID: ☐☐

CU – dropdown menu

Demographics

4. Location: ____________

5. Sub-location: ____________

6. Village name: ____________

Eligibility criteria

7. Did any member of the household have fever or malaria-like illness in the last four weeks? *If no, stop.*
   1. Yes
   2. No
   3. Don't Know

8. How long ago did the most recent fever start?

☐☐ Days

INSTRUCTION: if the ill individual is 18 or older and not present, stop and save the interview now; recording the time and date saved on your notebook; return to the saved interview if the ill adult is present on a return visit, use the recorded time and date to locate the form.

9. Are you still ill today?
   1. Yes
   2. No

Patient information

10. Gender of patient:

For peer review only - http://bmjopen.bmj.com/site/about/guidelines.xhtml

1. Male
2. Female

11. Relationship of patient to household head
    1. Head
    2. Spouse of head
    3. Child
    4. Parent
    5. Grandparent
    6. Grandchild
    7. Aunt /Uncle
    8. Brother / Sister
    9. Cousin
    10. Mother
    11. Father
    12. Employee
    13. OTHER____________________

12. Age of patient: [|] Years (if less than 18, skip to respondent information, question 15)

13. Highest school level:
    1. None
    2. Some Primary (not completed Class 8)
    3. Primary completed
    4. Primary completed and some secondary (secondary not completed)
    5. Secondary completed
    6. College
    7. University
14. Occupation
    1. Farming/Livestock keeping
    2. Home maker
    3. Student
    4. Paid employee
    5. Casual employee
    6. Self-employed (not agriculture, livestock) without employees
    7. Self-employed (not agriculture, livestock) with employees
    8. Unpaid family helper in a business
    9. Not working
    10. Unable to work/disabled
    11. Other (Specify)

Respondent (Answer if age of patient is <18 years. If patient is >18 years, skip to question 19.)

15. Age of respondent

For peer review only - http://bmjopen.bmj.com/site/about/guidelines.xhtml

☐☐ Years

16. Highest school level:
    1. None
    2. Some Primary (not completed Class 8)
    3. Primary completed
    4. Primary completed and some secondary (secondary not completed)
    5. Secondary completed
    6. College
    7. University

17. Gender of respondent:
    1. Male
    2. Female

18. Relationship of respondent to child:
    1. Parent
    2. Aunt/Uncle
    3. Grandparent
    4. Sibling
    5. Other

Endpoints *Note: The wording of the questions in this section are directed at a respondent 18 or over; when administered as an electronic survey, there will be two parallel sets of questions that branch based on whether a child or adult was the patient.*

19. How severe was the illness?
    1. Very severe
    2. Severe
    3. Moderately severe
    4. Mild
    5. Very mild

20. Did you take any action for your illness? *Note: taking drugs at home is an action. If "Don't remember" or "No", skip to COVARIATES section, question 56.)*

    1. Yes
    2. No
    3. Don't remember

21. What did you do FIRST for your illness? *Only one answer*
    1. Contact CHW

For peer review only - http://bmjopen.bmj.com/site/about/guidelines.xhtml

2. Go to a government health centre
3. Go to a government dispensary
4. Go to a government hospital
5. Go to a private clinic or mission facility
6. Go to a shop or pharmacy
7. Take some drugs at home
8. visit traditional healers
9. visit religious/cultural healers
10. OTHER: ____________

22. What were the MAIN reasons for deciding to do that? *Multiple answers OK*
1. I already had the drugs at home
2. It is the nearest place (facility or drug shop)
3. The services are free
4. Money- this was most affordable
5. Wanted to see a Doctor
6. Illness very serious
7. Illness not very serious
8. Time was limited / this was the fastest
9. Good quality service
10. There are no drugs in the facility
11. There is laboratory testing
12. OTHER: ____________

23. How long after the illness began did you do that?
1. same day
2. next day
3. after two days
4. more than two days later
5. OTHER: ____________

24. Did you take any other action? *If no, skip to question 26.*
1. Yes
2. No
3. Don't remember

25. If yes, what did you do? *Allow multiple answers*

1. Contact CHW
2. Go to a government health centre
3. Go to a government dispensary
4. Go to a government hospital
5. Go to a private clinic or mission facility
6. Go to a shop or pharmacy

For peer review only - http://bmjopen.bmj.com/site/about/guidelines.xhtml

7. Take some drugs at home
8. visit traditional healers
9. visit religious/cultural healers
10. OTHER: ___________

26. How long did you take to fully recover?

[|] Days (99=not yet recovered)

27. Did you have any laboratory test done during your illness? *PROBE. Ask about blood, stool or urine taken to help find out about the illness*

1. Yes
2. No
3. Don't remember

28. Did you have a malaria test? *If no or don't remember, skip to question 42.*

1. Yes
2. No
3. Don't remember
4. Don't know

29. How long after the start of the illness did you have a malaria test (the first test, if more than one)?

1. same day
2. next day
3. after two days
4. OTHER: ___________

30. Was it a microscopy test or a rapid diagnostic test? (If both, record in 'OTHER')

1. RDT
2. microscopy
3. Don't remember or don't know
4. OTHER (specify): _____________

31. Where was the malaria test done?

1. CHW (go to Question 32, else skip to 35.)
2. shop or pharmacy
3. private clinic/hospital
4. government dispensary
5. government hospital
6. government health center
7. private lab
8. OTHER: ___________

For peer review only - http://bmjopen.bmj.com/site/about/guidelines.xhtml

32. If a CHW did the test, where was it done?
    1. In my village
    2. In another village but the same CU (in which the survey is being conducted)
    3. Another CU (write name: ___________)
    4. Don't know/don't remember

33. Did the CHW give you a voucher to purchase a drug?
    1. Yes (Ask question 34 else skip to 35.)
    2. No
    3. Don't know/don't remember

34. Did you use the voucher to purchase a drug?
    1. Yes
    2. No
    3. Don't know/ don't remember

35. How much did the malaria test cost (Ksh.)? If more than one test, list both
    ______________**Free text**

36. What were the results of the malaria test? (If more than one test, use OTHER)
    1. Positive
    2. Negative
    3. Don't remember / Don't know
    4. OTHER________________

37. How likely is it that the result of your malaria test was correct?
    1. Very likely
    2. Likely
    3. 50-50
    4. Unlikely
    5. Very unlikely
    6. Don't know
    7. OTHER (use if have different beliefs about multiple tests)

38. Record observed for test? *Interviewer question (this can include study forms)*
    1. Yes
    2. No (Skip to question 42.)

If yes (record observed), interviewer should answer the next 3 questions:

39. Where does the record report the test was done? (multiple answers possible)

For peer review only - http://bmjopen.bmj.com/site/about/guidelines.xhtml

1. CHW
2. shop or pharmacy
3. private clinic/hospital
4. government dispensary
5. government hospital
6. government health center
7. private lab
8. Not specified / Cannot be determined
9. OTHER: ____________

40. What type of malaria test does the record report was done? (if more than one test, use OTHER)

1. RDT
2. microscopy
3. Not specified / Cannot be determined
4. OTHER (specify): ______________

41. What were the results of the malaria test according to the record? (if more than one test, use OTHER)

1. Positive
2. Negative
3. Not specified / Cannot be determined
4. OTHER ____________________

42. How likely is it that the illness was malaria?

1. Very likely
2. Likely
3. 50-50
4. Unlikely
5. Very unlikely
6. Don't know
7. No response

43. Were any drugs taken for the illness? *If no or don't remember, skip to question 55.*

1. Yes
2. No
3. Don't remember

44. Which medicines did you take? (Multiple answers possible) SKIP PATTERN: If 1 or 4 (ACT), continue with next question. Else, skip to 53

1. Coartem/Artefan/other AL
2. Quinine

For peer review only - http://bmjopen.bmj.com/site/about/guidelines.xhtml

3. SP/Fansidar/Metakelfin/Moladar
4. Other ACT (e.g Duocotexin)
5. Amoxyl
6. Septrin
7. Ciprofloxacin/Norfloxaccin
8. Panadol/Brufen/Hedex/Painkiller/Action/Maramoja
9. Don't know/Don't Remember
10. Other (specify: __________)

45. Packaging observed for ACT? *Interviewer question. If YES, ask question 46. If NO, skip to question 47.*
1. Yes
2. No

46. If ACT package observed, what brand? *Interviewer question*.
1. Coartem (AL)
2. Aretefan (AL)
3. Duocotexin
4. P-Alaxin
5. AL (IPCA brand)
6. AQ AS
7. Lonart (AL)
8. Artequick
9. Lumartem (AL)
10. Combiart (AL)
11. L-artem (AL)
12. OTHER: ___________

47. How long after the start of the illness did you start taking the ACT?
1. same day
2. next day
3. after two days
4. OTHER: ___________

48. Did you start taking the ACT before or after the malaria test? *Only ask this if YES to 28.*
1. Before
2. After
3. Don't remember
4. Not Applicable

If respondent answered "1-Coartem/Artefan/other AL" to question 44, ask questions 49-52, else skip to question 53.

49. If AL, how many pills were given? *Only ask if 44 = 1 (AL ONLY)*

For peer review only - http://bmjopen.bmj.com/site/about/guidelines.xhtml

1. 6
2. 12
3. 18
4. 24
5. Don't know/remember
6. OTHER: ___________

50. If AL, did you / patient take all of the tablets? *Only ask if 44 = 1 (AL ONLY)*

1. Yes
2. No
3. Not sure
4. Don't remember
5. Not yet—still taking them

51. Where was the AL obtained? MULTIPLE RESPONSES POSSIBLE *(If the drugs were already in the home at the onset of illness, record this but find out from where they were originally obtained) Only ask if 44 = 1, 4 ie any ACT.*

1. Used drugs at home
2. Pharmacy, chemist or duka la dawa with a prescription
3. Pharmacy, chemist or duka la dawa; no prescription
4. CHW
5. Neighbor
6. Private health facility
7. Government health facility
8. general store
9. OTHER: _______________
10. Don't remember/don't know

52. Did you get AL at the first place you sought treatment? *Only ask if 44 = 1,4 (ACT ONLY)*

1. Yes
2. No
3. Yes and another place
4. Don't know/don't remember

53. How likely do you think the malaria drugs you took were effective? (ask if respondent answered 1, 2, 3 or 4 to question 44.)

1. Very likely
2. Likely
3. 50-50
4. Unlikely

For peer review only - http://bmjopen.bmj.com/site/about/guidelines.xhtml

5. Very unlikely
6. Don't know
7. No response

54. How much did you spend for all the drugs you purchased for this illness? (-1 should be used for 'I don't know/don't remember')
 1. ___________________________
55. How much did you spend in total to take care of this illness, including testing, drugs, transportation, admission fees, registration and consultation fees? (-1 should be used for 'I don't know/don't remember')
 1. ____________________________

Covariates *THESE QUESTIONS DIRECTED AT RESPONDENT, EVEN IF NOT THE PATIENT*

56. GPS coordinates: _____ (PLACED AT THE END OF THE ELECTRONIC SURVEY)

57. Does your household have any mosquito nets? (*If no or don't know, skip to question 60.*)
 1. Yes
 2. No
 3. DK

58. Did THE PATIENT sleep under the net last night?
 1. Yes
 2. Has a net for sleeping space, but did not use it last night.
 3. Does not have net for sleeping space
 4. Do not Remember/Don't Know

59. Was the net pre-treated with insecticide when you got it?
 1. Yes
 2. No
 3. Not Applicable (no net for sleeping space)
 4. Do not Remember/Don't Know

60. Is there a CHW in your village that provides any services or health education? *If no or do not know skip to question 66.*
 1. Yes
 2. No
 3. Don't Know

61. Which services does the CHW provide? *Multiple responses possible*
 1. Information

For peer review only - http://bmjopen.bmj.com/site/about/guidelines.xhtml

2. Referral
3. Polio immunization
4. Advice
5. Testing for HIV
6. Testing for malaria
7. Testing for diabetes
8. Testing for hypertension
9. Testing for pregnancy
10. Coupon for antimalarial drug
11. Bednets
12. Water treatment (ie Lifestraw)
13. Other (specify) ___________________________

62. Does your CHW provide malaria diagnostic testing?
    1. Yes
    2. No
    3. Don't know/Don't remember

63. Does your CHW provide a discount on a malaria drug in case the test is positive for malaria?
    1. Yes
    2. No
    3. Don't know/Don't remember

64. Have you ever contacted a CHW near you in case of illness for yourself or someone in your household?
    1. Yes
    2. No (*skip to question 66*)
    3. DR/DK (*skip to question 66*)

65. What did the CHW do? (multiple responses possible)
    1. Refer
    2. Give medicine
    3. Accompany to shop
    4. Give advice
    5. Perform a malaria test
    6. Give a coupon for an antimalarial drug
    7. Other (specify)____________________

66. Do you know what a rapid diagnostic test for malaria is? *Show example of cassette*
    1. Yes
    2. No → skip to 71

67. Have you ever had an RDT test for malaria?
    1. Yes
    2. No
    3. Don't know/don't remember

68. Do you know anyone besides one of your household members who has had an RDT?
    1. Yes
    2. No

For peer review only - http://bmjopen.bmj.com/site/about/guidelines.xhtml

3. Don't know/don't remember

69. If you have fever and your malaria RDT is negative, how likely is it that the test is correct?

1. Very likely
2. Likely
3. 50-50
4. Unlikely
5. Very unlikely
6. Don't know
7. No response

70. If you have fever and your malaria RDT is positive, how likely is it that the test is correct?

1. Very likely
2. Likely
3. 50-50
4. Unlikely
5. Very unlikely
6. Don't know
7. No response

71. Is malaria common in your village

1. Yes
2. No
3. Don't know/not sure

72. If ten people (watu) from your village have fever, how many of them are likely to have malaria? _____________(number 0-10 or 99 for don't know)

73. If you have malaria, and you take AL, how likely is it that you will be completely better in 3 days?

1. Very likely
2. Likely
3. 50-50
4. Unlikely
5. Very unlikely
6. Don't know
7. No response

SES Questions
(Source:pilot)

74. Does your household have the following items

1. Electricity
2. Television
3. Refrigerator
4. Radio

For peer review only - http://bmjopen.bmj.com/site/about/guidelines.xhtml

5. Mobile phone
6. Motorcycle
7. car/truck
8. Bank account
9. None

75. How many of the following livestock does your household have
    1. Cows
    2. Sheep
    3. Goats
    4. Pigs
    5. Donkeys

76. What kind of toilet does your household have
    1. Flush or pour flush toilet
    2. VIP/Ventilated improved pit
    3. Pit latrine WITH slab
    4. Pit latrine without slab
    5. Composting toilet
    6. Bucket toilet
    7. No facility/bush/field
    8. Other

77. Main source of drinking water for your household
    1. Piped water/public tap/borehole
    2. Unprotected well
    3. Protected well
    4. Protected spring
    5. Unprotected spring
    6. Rain Water
    7. River Water
    8. Other:______________

78. Main material of the floor in your house
    1. Earthen
    2. Cement
    3. Floor tiles
    4. Wood planks
    5. Polished wood
    6. Other

79. Main material of the walls in your house
    1. Stone
    2. Brick
    3. Timber
    4. Iron Sheet
    5. Mud

For peer review only - http://bmjopen.bmj.com/site/about/guidelines.xhtml

6. Wood
7. Cement
8. Other

80. Main material of the roof of your house

1. Iron sheets
2. Roof tiles
3. Grass thatched
4. Wood

81. How many acres/hectares/feet of land for farming does your household own

1. None
2. Acres: ___________
3. Hectares: ________
4. Square feet(xx by xx):_________

For peer review only

For peer review only - http://bmjopen.bmj.com/site/about/guidelines.xhtml

STROBE Statement—Checklist of items that should be included in reports of ***cross-sectional studies***

	**Item No**	**Recommendation**	**Page No**
**Title and abstract**	1	(*a*) Indicate the study's design with a commonly used term in the title or the abstract	1
		(*b*) Provide in the abstract an informative and balanced summary of what was done and what was found	2-3
**Introduction**			
Background/rationale	2	Explain the scientific background and rationale for the investigation being reported	4-5
Objectives	3	State specific objectives, including any prespecified hypotheses	5
**Methods**			
Study design	4	Present key elements of study design early in the paper	6
Setting	5	Describe the setting, locations, and relevant dates, including periods of recruitment, exposure, follow-up, and data collection	6-7
Participants	6	(*a*) Give the eligibility criteria, and the sources and methods of selection of participants	8
Variables	7	Clearly define all outcomes, exposures, predictors, potential confounders, and effect modifiers. Give diagnostic criteria, if applicable	10
Data sources/ measurement	8*	For each variable of interest, give sources of data and details of methods of assessment (measurement). Describe comparability of assessment methods if there is more than one group	*9-10*
Bias	9	Describe any efforts to address potential sources of bias	
Study size	10	Explain how the study size was arrived at	8
Quantitative variables	11	Explain how quantitative variables were handled in the analyses. If applicable, describe which groupings were chosen and why	10
Statistical methods	12	(*a*) Describe all statistical methods, including those used to control for confounding	11-12
		(*b*) Describe any methods used to examine subgroups and interactions	
		(*c*) Explain how missing data were addressed	
		(*d*) If applicable, describe analytical methods taking account of sampling strategy	11
		(*e*) Describe any sensitivity analyses	
**Results**			
Participants	13*	(a) Report numbers of individuals at each stage of study—eg numbers potentially eligible, examined for eligibility, confirmed eligible, included in the study, completing follow-up, and analysed	14
		(b) Give reasons for non-participation at each stage	
		(c) Consider use of a flow diagram	
Descriptive data	14*	(a) Give characteristics of study participants (eg demographic, clinical, social) and information on exposures and potential confounders	14
		(b) Indicate number of participants with missing data for each variable of interest	
Outcome data	15*	Report numbers of outcome events or summary measures	15, Table 1

For peer review only

For peer review only - http://bmjopen.bmj.com/site/about/guidelines.xhtml

Main results	16	(*a*) Give unadjusted estimates and, if applicable, confounder-adjusted estimates and their precision (eg, 95% confidence interval). Make clear which confounders were adjusted for and why they were included	Table 2-6
		(*b*) Report category boundaries when continuous variables were categorized	Tables
		(*c*) If relevant, consider translating estimates of relative risk into absolute risk for a meaningful time period	
Other analyses	17	Report other analyses done—eg analyses of subgroups and interactions, and sensitivity analyses	
**Discussion**			
Key results	18	Summarise key results with reference to study objectives	18
Limitations	19	Discuss limitations of the study, taking into account sources of potential bias or imprecision. Discuss both direction and magnitude of any potential bias	20
Interpretation	20	Give a cautious overall interpretation of results considering objectives, limitations, multiplicity of analyses, results from similar studies, and other relevant evidence	21
Generalisability	21	Discuss the generalisability (external validity) of the study results	20
**Other information**			
Funding	22	Give the source of funding and the role of the funders for the present study and, if applicable, for the original study on which the present article is based	22

*Give information separately for exposed and unexposed groups.

Note: An Explanation and Elaboration article discusses each checklist item and gives methodological background and published examples of transparent reporting. The STROBE checklist is best used in conjunction with this article (freely available on the Web sites of PLoS Medicine at http://www.plosmedicine.org/, Annals of Internal Medicine at http://www.annals.org/, and Epidemiology at http://www.epidem.com/). Information on the STROBE Initiative is available at www.strobe-statement.org.

For peer review only

For peer review only - http://bmjopen.bmj.com/site/about/guidelines.xhtml

BMJ Open

Supply- and demand-side factors influencing uptake of malaria testing services in the community: lessons for scale-up from a post-hoc analysis of a cluster-randomised, community-based trial in western Kenya

Journal:	*BMJ Open*
Manuscript ID	bmjopen-2022-070482.R3
Article Type:	Original research
Date Submitted by the Author:	31-May-2023
Complete List of Authors:	Kirui, Joseph; Academic Model Providing Access to Healthcare, AMPATH<br>Malinga, Josephine; Duke Global Health Institute<br>Sang, Edna; Duke Global Inc, Kenya<br>Ambani, George; Academic Model Providing Access to Healthcare<br>Abel, Lucy; Academic Model Providing Access to Healthcare<br>Nalianya, Erick; Duke Global Inc, Kenya<br>Namae, Jane; Moi University College of Health Sciences<br>Boyce, Matthew; Georgetown University<br>Laktabai, Jeremiah; Academic Model Providing Access to Healthcare; Moi University College of Health Sciences<br>Menya, Diana; Moi University School of Public Health<br>O'Meara, Wendy; Duke Global Health Institute, ; Moi University,
**Primary Subject Heading**:	Global health
Secondary Subject Heading:	Health services research, Infectious diseases
Keywords:	Public health < INFECTIOUS DISEASES, Community child health < PAEDIATRICS, International health services < HEALTH SERVICES ADMINISTRATION & MANAGEMENT

SCHOLARONE™
Manuscripts

I, the Submitting Author has the right to grant and does grant on behalf of all authors of the Work (as defined in the below author licence), an exclusive licence and/or a non-exclusive licence for contributions from authors who are: i) UK Crown employees; ii) where BMJ has agreed a CC-BY licence shall apply, and/or iii) in accordance with the terms applicable for US Federal Government officers or employees acting as part of their official duties; on a worldwide, perpetual, irrevocable, royalty-free basis to BMJ Publishing Group Ltd ("BMJ") its licensees and where the relevant Journal is co-owned by BMJ to the co-owners of the Journal, to publish the Work in this journal and any other BMJ products and to exploit all rights, as set out in our licence.

The Submitting Author accepts and understands that any supply made under these terms is made by BMJ to the Submitting Author unless you are acting as an employee on behalf of your employer or a postgraduate student of an affiliated institution which is paying any applicable article publishing charge ("APC") for Open Access articles. Where the Submitting Author wishes to make the Work available on an Open Access basis (and intends to pay the relevant APC), the terms of reuse of such Open Access shall be governed by a Creative Commons licence – details of these licences and which Creative Commons licence will apply to this Work are set out in our licence referred to above.

Other than as permitted in any relevant BMJ Author's Self Archiving Policies, I confirm this Work has not been accepted for publication elsewhere, is not being considered for publication elsewhere and does not duplicate material already published. I confirm all authors consent to publication of this Work and authorise the granting of this licence.

For peer review only

Supply- and demand-side factors influencing uptake of malaria testing services in the community: lessons for scale-up from a post-hoc analysis of a cluster-randomised, community-based trial in western Kenya

Authors and affiliations:

Joseph Kirui[1*], Josephine Malinga[2], Edna Sang[3], George Ambani[1], Lucy Abel[1], Erick Nalianya[3], Jane Namae[4], Matthew Boyce[5], Jeremiah Laktabai[1,4], Diana Menya[6], Wendy Prudhomme O'Meara[2,6]

[1]Academic Model Providing Access to Healthcare, Eldoret, Kenya.

[2]Duke Global Health Institute, Durham, North Carolina, USA.

[3]Duke Global Inc, Kenya.

[4]School of Medicine, Moi University College of Health Sciences, Eldoret Kenya.

[5]Georgetown University, Washington DC, USA.

[6]School of Public Health, Moi University College of Health Sciences, Eldoret, Kenya.

[*]**Correspondence to:**

Joseph Kirui

Academic Model Providing Access to Healthcare, Eldoret, Kenya

Email: josepheddykipkoech@gmail.com

Keywords: Community-based programs, demand and supply, health interventions, malaria, diagnostic testing, Community Health Workers

For peer review only

List of abbreviations: Artemisinin Combination Therapy (ACT), Artemether Lumefantrine (AL) Community Health Extension Worker(CHEW), Community Health Worker (CHW), Community Unit (CU), Global Positioning System (GPS), Ministry of Health (MOH), Malaria Rapid Diagnostic Test (mRDT), World Health Organization (WHO).

Abstract

Objectives: Maximizing the impact of community-based programs requires understanding how supply of, and demand for, the intervention interact at the point of delivery.

Design: Post-hoc analysis from a large-scale community health worker (CHW) study designed to increase the uptake of malaria diagnostic testing.

Setting: Respondents were identified during a household survey in western Kenya between July 2016 and April 2017.

Participants: Household members with fever in the last four weeks were interviewed at 12 and 18-months post-implementation. We collected monthly testing data from 244 participating CHWs and conducted semi-structured interviews with a random sample of 70 CHWs.

Primary and secondary outcome measures: The primary outcome measure was diagnostic testing before treatment for a recent fever. The secondary outcomes were receiveing a test from a CHW and tests done per month by each CHW.

For peer review only - http://bmjopen.bmj.com/site/about/guidelines.xhtml

Results: 55% (n=948/1738) reported having a malaria diagnostic test for their recent illness of which 38.4% were tested by a CHW. Being aware of a local CHW (Adj.OR=1.50, 95%CI:1.10-2.04) and belonging to the wealthiest households (vs. least wealthy) was associated with higher testing (Adj.OR=1.53, 95%CI:1.14-2.06). Wealthier households were *less* likely to receive their test from a CHW compared to poorer households (Adj.OR=0.32, 95%CI:0.17-0.62). Confidence in artemether-lumefantrine (AL) to cure malaria (Adj.OR=2.75, 95%CI:1.54-4.92) and perceived accuracy of an mRDT (Adj.OR=2.43, 95%CI:1.12-5.27) were positively associated with testing by a CHW. Specific CHW attributes were associated with performing a higher monthly number of tests including formal employment, serving more than 50 households (vs<50) and serving areas with a higher test positivity. On demand-side, confidence of the respondent in a test performed by a CHW was strongly associated with seeking a test from a CHW.

Conclusion: Scale-up of community-based malaria testing is feasible and effective in increasing uptake among the poorest households. To maximize impact, it is important to recognize factors that may restrict delivery and demand for such services.

Strengths and limitations of this study

- This analysis draws on a large multidimensional dataset describing uptake of community-based services (intervention recipeints) and delivery of services (intervention providers) in the context of a cluster-randomised implementation trial, thereby allowing us to examine the strengths and shortcomings of the intervention by triangulating data from two unique perspectives.

For peer review only - http://bmjopen.bmj.com/site/about/guidelines.xhtml

- These data were from a representative population sample which include respondents that were successfully reached with the intervention as well as those who were not tested for malaria before taking antimalarials and may require alternative approaches in order to achieve adequate coverage of diagnostic testing before treatment with antimalarials.
- As a secondary analysis of trial data, we were limited in the scope of the data available and not able to fully explore the interaction between the CHWs and households nor the complex determinants of health seeking behavior.

For peer review only

For peer review only - http://bmjopen.bmj.com/site/about/guidelines.xhtml

Introduction

Despite decades of control efforts, malaria remains a major public health problem. In 2019, an estimated 229 million cases of malaria occurred worldwide, most of which were in the World Health Organization (WHO) African Region (215 million or 94%).[1] In the same year, there were an estimated 409 000 deaths from malaria globally, with Africa again accounting for 94%. [1] Children under 5 years are the most vulnerable group affected by malaria and in 2019, 67% of malaria deaths were in this group.[1]

Prompt malaria diagnosis either by microscopy or rapid diagnostic tests (mRDTs) is recommended by WHO for all patients with suspected malaria before they are given treatment.[2] Already, 95 countries have adopted the policy of testing all patients with suspected malaria before being treated, and this number is increasing. Diagnostic testing is free of charge in the public sector of almost 90 countries. [3] Early and accurate diagnosis is essential both for effective management of the disease and for strong malaria surveillance. Parasite-based diagnostic testing significantly reduces illness and death by enabling health providers to swiftly distinguish between malarial and non-malarial fevers and select the most appropriate treatment. [4]

The Kenya National Control Program adopted the Test and Treat policy in 2010 and this has been cascaded to the community level. The mRDTs are recommended for parasitological diagnosis in public health facilities where microscopy is not available and through Community Health Workers in some regions. Despite these efforts, most people in rural Kenya still opt for self-treatment and seek care in the retail health sector. [5,6] In our study setting, mRDT are not available in pharmacies and chemists, therefore, patients who purchase antimalarials in the retail sector receive no parasitological diagnosis before treatment. Studies have shown that targeting of antimalarials to true malaria cases in the retail sector is very low: as few as 20% of people purchasing

For peer review only

For peer review only - http://bmjopen.bmj.com/site/about/guidelines.xhtml

antimalarials are suffering from malaria [7,8] and as high as 70% of people with malaria do not purchase an ACT [9].

The deployment of mRDTs through trained community health workers could improve access to diagnosic testing for populations with limited access to health facilities. In a large, cluster-randomised trial[6], we evaluated an intervention that targeted individuals who seek care in the retail sector by offering free community-based malaria diagnostic testing through CHWs. Following testing, those with a positive test result received a voucher to obtain first-line antimalarials at a nearby pharmacy at a heavily subsidized price. The intervention demonstrated a significant improvement in testing before treatment.

Ideally, there should be adequate demand for the commodity or opportunity, and supply should be able to meet demand. In our cluster randomized trial, despite improvements in malaria testing before treatment of fever, we still observed that only 55% of those with recent illness reported having a malaria test before taking drugs and 21% still purchased antimalarials over-the-counter without a test[6], representing substantial scope for further improving the reach and impact of this intervention. We predicted that the intervention could have led to higher testing rates and sought to understand whether the impact may have been limited by either demand for, or supply of, testing or both.

Here we report a secondary analysis of trial data collected at 12 and 18 months after the intiatiion of our cluster-randomised trial of community-based diagnostic testing in western Kenya[6]. We sought to understand what factors might increase or decrease demand for testing from the community, compared to factors that may have limited supply of testing by CHWs. A comparison of constraints on the demand-side and supply-side could help us understand the conditions under which the intervention operated well or was hindered by a mismatch in supply

For peer review only - http://bmjopen.bmj.com/site/about/guidelines.xhtml

and demand. The results will be instrumental for improving the design of community-based interventions delivered at scale.

Methods

Description of the main trial

This is a post-hoc analysis of data collected within intervention clusters during a large community based cluster randomised trial. The main results of the trial have been reported elsewhere[6]. The purpose of the parent trial was to encourage clients to receive testing for malaria before seeking treatment. This was accomplished by training and equipping community health workers (CHWs) to perform rapid-diagnostic tests (mRDTs) for malaria and to provide clients who test positive for malaria with a voucher that allows them access to subsidized high-quality antimalarial drugs through participating pharmacies.[6] The trial was conducted in 32 community clusters in western Kenya (population approximately 160,000). Eligible clusters had retail outlets selling AL and existing community health worker (CHW) programs and were randomly assigned 1:1 to control and intervention arms. In intervention areas (comprised of 16 community units), CHWs were available in their villages to perform malaria rapid diagnostic tests (mRDTs) on demand for any individual >1 year of age experiencing a malaria-like illness. The mRDT-positive individuals received a voucher for a discount on a quality-assured AL, redeemable at a participating retail medicine outlet. In control cluster (composed of 16 community units), CHWs offered a standard package of health education, prevention, and referral services. We conducted 4 population-based surveys—at baseline, 6 months, 12 months, and 18 months—of a random sample of households with fever in the last 4 weeks to evaluate predefined, individual-level trial outcomes.

For peer review only - http://bmjopen.bmj.com/site/about/guidelines.xhtml

In Kenya, CHWs work on a volunteer basis and there are no formal education requirements.The ability to read and write are criteria for selection by the MOH. The role of CHWs as defined by the MOH is to promote good health through providing health education, basic treatments (such as first-aid), and referrals to health care facilities. The CHWs report to Community Health Extension Workers (CHEWs) who are employees of MOH and are trained community health nurses mandated to supervise the activities of CHWs in the community units. In intervention areas, existing CHWs were trained to carry out the intervention. 272 CHWs participated in offering testing, a median of 18 CHWs per cluster (range: 10-24).

CareStart HRP2-based malaria mRDTs were used for testing. CareStart HRP2 detects *Plasmodium falciparum* which is the main species of parasite responsible for the majority of malaria cases in Kenya.[10] In addition to malaria mRDT, the study team provided CHWs with the necessary supplies to conduct the mRDT testing. CHWs were free to perform tests in the location(s) of their choice (e.g., in their own homes, at clients' homes, etc.), as long as the location allowed for proper and sufficiently private performance of the procedure.

The CHWs tested febrile patients at community level and issued AL vouchers to patients with positive results. This CHW model differs from other settings implementing community case management of malaria where the CHW is permitted to both perform the mRDT and provide antimalarials directly to the client. The voucher redemption period was three days where by after that the Medicine shops were not allowed to collect the voucher. Patients with positive malaria results bought AL from medicine shops by redeeming their vouchers and leaving the voucher at the shop. The study team collected the redeemed vouchers from the medicine shops on a bi-weekly basis and reimbursed the difference in costs.

For peer review only - http://bmjopen.bmj.com/site/about/guidelines.xhtml

Community surveys

A community-based survey was conducted to collect individual-level study outcomes based on a population-based survey sampling strategy. This was a repeated cross-sectional household survey at 4 time points: baseline (pre-intervention), 6 months, 12 months, and 18 months post baseline. The survey was conducted in all the 32 community units, 16 Intervention and 16 control. Households were randomly selected by systematic random sampling and one person with fever in the last four weeks was interviewed in any household. If more than one eligible fever was found in a household, one participant would be selected based on alphabetical order of the given names. Details of community surveys sample size calculation are described elsewhere.[11] Briefly, the target sample size of eligible individuals with a fever in the last 4 weeks was 640 per arm (i.e., 40 per CU) at each time point, which—assuming 22.2% of households would have an eligible member—would require contacting 5,766 households. The starting point (index house) and sampling interval were different in each survey round in order to minimize repeat surveys across multiple rounds. We restrict our analysis here to the 12 and 18 month surveys in order to evaluate the effects of interest in the time-period when the intervention effect could be measured.[6]

Basic demographic information about the participants and the households were collected. Socioeconomic scores were calculated from an asset index and then grouped into quintiles as described previously. Euclidean distance from a household to the nearest health facility was calculated from GPS coordinates. In the final survey, participants beliefs about their illness, malaria risk, diagnostic testing, and treatment were elicited by asking them about the severity of their illness, their confidence in positive test result and a negative test result, and how certain they were that they would recover from malaria after treatment with AL. "Perceived prevalence" was defined as the proportion of fevers that the respondent believed would be due to malaria among

For peer review only - http://bmjopen.bmj.com/site/about/guidelines.xhtml

10 people with fever from their village. High perceived prevalence was assigned to those who responded 8-10 out of 10, medium prevalence to those who responded 4-7 out of 10 and low to those who responded 0-3 out of 10. The survey tool can be found in supplemental materials.

Midpoint CHW survey

As part of the process evaluation, at the midpoint of this study, interviews were conducted with a random sample of 70 CHWs (median of 4 per CU) who were trained to conduct mRDTs (intervention CUs). The primary objective of these interviews were to determine the CHWs' satisfaction with their role in the malaria project and the feasibility of their continued involvement. Secondary objectives included eliciting the main challenges the CHWs have faced in implementing the study, determining unanticipated burdens on the CHWs' other activities (including their responsibilities as CHWs outside of the study as well as their non-CHW responsibilities) and identifying changes in the types of services they provide and the recipients of their services. [12]

Likert-scale questions were also asked, in which the interviewers read a statement aloud and the CHWs were asked to respond how much they agreed with the statement on a five-point scale ("strongly agree", "somewhat agree," "neutral," "somewhat disagree," "strongly disagree"). For this analysis, several variables were derived from the survey data. The client score was constructed as a composite score based on four likert scale questions – whether CHWs agreed that; clients trust the results of a positive mRDT by the CHW, they trust a negative mRDT, they follow the CHWs advice if the test was positive, they follow advice if the test was negative. A response of 'strongly agree'=2, somewhat agree='1' and neutral/somewhat disagree/strongly, disagree=0. If the sum of these four responses was 6 or greater, the score was 'high'. If less than 6, the score was 'low'. The survey tool can be found in supplemental materials.

For peer review only - http://bmjopen.bmj.com/site/about/guidelines.xhtml

These same CHWs also performed an mRDT under observation of the study supervision team and were scored on each step using a 20-step checklist that classified steps as relating to safety, accuracy, and treatment. The checklist was developed by the study team based on previous research studies to guide training and support supervision. Those who completed at least 17 of 20 steps correctly had a 'high' score for mRDT performance and <17 had a 'low' score.

Demand and supply

To investigate factors that might have influenced the success of the intervention, the analysis was divided into demand and supply factors. On the demand-side, the analysis investigates factors that might influence the community member's outcome of having a test, first from any source and then from the CHW. Only respondents with complete data on the variables of interest were included. On the supply-side, the analysis investigates the factors associated with the testing output (i.e. supply) at the CHW level, measured as the monthly number of tests conducted by each CHW. These factors include; socio-demographic characteristics of the CHW, number of households under the CHWs care, years of experience of the CHW, the number of other activities the CHW engages in under their role as a CHW (under the assumption that an increasing number of activites may compete for their time, the CHW provided a list of activities and these were summed together), the perception of the intervention by the CHW, the skill of the CHW in conducting mRDTs and the overall prevalence of malaria in their community.

Outcome measures

The primary outcome is the uptake of testing in the intervention clusters, defined as the proportion of fevers in the previous 4 weeks that received a diagnostic test from any source. The second

For peer review only - http://bmjopen.bmj.com/site/about/guidelines.xhtml

primary outcome is the uptake of testing from a community health worker among those who received a diagnostic test from any source.

The secondary outcome was the testing volume, defined as the mean monthly number of tests performed by each CHW. Monthly number of tests per CHW was available from program data collected at monthly supervision visits.

Statistical analysis

A mixed-effects logistic regression analysis was used to evaluate the primary outcome measures on the uptake of testing and investigate risk factors that might have contributed to the outcomes of the intervention reported previously.[6] We adopt a mixed effects logistic regression to account for clustering at the level of the CU. The outcomes were evaluated only for the CUs in the intervention arm and summaries provided.

For the primary outcome measures to evaluate the uptake of testing overall and from a CHW, two logistic models were generated. The first model included all individuals who reported fever in the last 4 weeks (ie all eligible participants enrolled in the survey). The second model includes a subset of those in the first model, specifically those who were tested at any source. For both models, we accounted for clustering within the CU in the univariate analysis and we accounted for clustering, and individual-level covariates like respondent's age, gender, level of education, occupation, socio-economic status, geographic proximity to health care provider, the time they waited after falling ill before they sought a test, the level of knowledge and opinions about malaria and malaria diagnosis in the multivariate analysis.

The logistic regression model is as shown below:

For peer review only - http://bmjopen.bmj.com/site/about/guidelines.xhtml

$$log\left(\frac{p_{ij}}{1-p_{ij}}\right) = \beta_0 + \beta_1 X_{1j} + \dots + \beta_m X_{mj} + u_j$$

where p_{ij} is the probability of the outcome for each individual i in cluster j, β_0 is the model intercept, β_{1-m} are the coefficients for each potential risk factor X_{1-m}, and u_j represents the random effects for cluster j. The structures for both models are the same, however, for model 1, any test=1 and no test=0 and for or model 2, tested at CHW=1 and tested elsewhere=0.

Fixed effects are measured using an Odds Ratio (OR) and included the 95% confidence intervals. Independent categorical variables were screened for multicollinearity with a Cramér's V statistic. In case two variables showed signs of correlation (V > 0.7), the variable least associated with the response variable was omitted.[13]

For the secondary outcome measure of CHW testing, a mixed-effects linear regression model was fitted to account for clustering at the CU. This model investigates the outcome of mean RDTs performed each month by the specific CHW and includes potential CHW-level covariates like CHW age, marital status, education level, number of households for which they were responsible, previous training on malaria case management or mRDTs, years of experience as a CHW, and formal employment status.

The linear regression model is as shown below:

$$Y_{ij} = \beta_0 + \beta_1 X_{1j} + \dots + \beta_m X_{mj} + u_j + \varepsilon_{ij}$$

where Y_{ij} is the outcome for each individual i in cluster j, β_0 is the model intercept, β_{1-m} are the co-efficients for each potential risk factor X_{1-m}, u_j represents the random effects for cluster j, and ε_{ij} is the error term.

For peer review only - http://bmjopen.bmj.com/site/about/guidelines.xhtml

All statistical analysis was done in STATA version 15 (StataCorp).

Patient and public involvement

None.

For peer review only

For peer review only - http://bmjopen.bmj.com/site/about/guidelines.xhtml

Results

A total of 272 CHWs in 16 Intervention CUs were trained to perform mRDTs and 36 retail shops were enrolled to redeem vouchers. The intervention was launched on July 21, August 17, and September 22, 2015 in Bokoli, Kiminini, and Ndivisi sub-counties western Kenya, respectively and continued until May 5, 2017.

Demand-side factors for use of malaria diagnostic testing

In total, 3719 households were approached, 1877 had an eligible fever in the last four weeks, 56 did not know if there was an eligible fever, 0 refused and 1738 completed the survey with no missing answers (92.6%). The community-based demand analysis relies on 1738 complete surveys from participants in randomly selected households in intervention clusters with an acute illness in the last four weeks (**Table 1**). Community members were interviewed at 12 or 18 months (47.2% and 52.8% respectively) following the initiation of the intervention. 55% (n= 948) of participants reported having a malaria test for their recent illness while 38.4% (n=364/948) of those tested reported having had the test at CHW. Among all surveyed participants, 41% (n= 710) were male and 59% (n= 1028) were female. 21.2% (n= 368) of the recent fevers were aged <5 years, 43.3% (n= 752) were 5 -17 years while 36% (n= 618) were 18> years. Only 21.4% (n= 371) of the participants had completed their secondary education while 45% (n= 778) had completed their primary education. Overall 42.8% lived more than 2km from their nearest public health facility.

Demand-side factors that were associated with uptake of malaria testing for their most recent illness are examined (**Table 1**). Respondents who delayed seeking care until the day after they fell ill had a higher odds of receiving a test than those who sought care on the same day, but there

For peer review only - http://bmjopen.bmj.com/site/about/guidelines.xhtml

was no difference between those who sought care the same day and those who waited until the second day. Being aware of your local CHW increased the odds of having a test from any source, facility or CHW (Adj. OR=1.50, 95% CI: 1.10-2.04). Respondents from the wealthiest households were 53% more likely to have a test compared to the least wealthy households (Adj. OR=1.53 95%CI: 1.14-2.06). Being an adult at least 18 years old was negatively associated to taking a test (Adj. OR=0.59, 95% CI:0.43-0.82) compared to children 1-4 years. Distance to a health facility greater than 2km (Adj. OR=0.86, 95% CI:0.67-0.11) and use of ITN (Adj. OR=1.27, 95%CI:0.93-1.74) were not significantly associated with testing uptake.

Table 1. Participant characteristics at the 12 and 18 month survey and association between participant characteristics and malaria diagnostic testing. Adjusted and unadjusted odds ratios for receiving a malaria diagnostic test, either microscopy or mRDT, from any source for the most recent febrile illness.

	**Sample characteristics**	**Univariate analysis**		**Multivariate analysis**	
	(N=1738)	Unadjusted odds ratio (95%CI)	P-value	Adjusted odds ratio (95%CI)	P-value
Outcome=tested by any source	948/1738 (54.6%)				
Time to seek care (*Reference=same day*)	775 (44.4%)				
Next day	559 (32.3%)	**1.21[1.05,1.42]**	**0.01**	**1.23[1.06,1.43]**	**0.01**
The second day	223 (12.8%)	1.01[0.78,1.32]	0.94	1.13[0.86,1.48]	0.38
More than two days	182 (10.5%)	0.94[0.73,1.22]	0.66	1.12[0.87,1.44]	0.37
Use an ITN *(Reference=does not use an ITN)*	1387 (79.8%)	1.37[0.99,1.92]	0.06	1.27[0.93,1.74]	0.13
Distance to public health facility >2km (*Reference = <2km*)	837 (48.2%)	0.83[0.64,1.06]	0.14	0.86[0.67,1.11]	0.26
Aware of a CHW in their village	1424 (81.9)	**1.43[1.01,2.05]**	**0.04**	**1.50[1.10,2.04]**	**0.01**

For peer review only - http://bmjopen.bmj.com/site/about/guidelines.xhtml

(Reference=is not aware of a CHW in their village)					
Patient age (*Reference=1-4y*)	368 (21.1%)			-	-
5 to 17y	752 (43.3%)	0.76[0.54,1.08]	0.12	0.79[0.56,1.12]	0.18
18+y	618 (35.6%)	**0.59[0.43,0.81]**	**<0.01**	**0.59[0.43,0.82]**	**<0.01**
Patient is male (*Reference = female*)	710 (40.9%)	1.02[0.86,1.23]	0.78	0.94[0.80,1.10]	0.43
Respondent education level (*Reference=not completed primary*)	589 (33.9%)			-	-
Completed primary	778 (44.8%)	1.12[0.93,1.36]	0.23	1.08[0.87,1.33]	0.48
Completed secondary	371 (21.3%)	**1.41[1.04,1.90]**	**0.03**	1.25[0.93,1.70]	0.14
Wealth quintile (*Reference=0-20th*)	466 (26.8%)			-	-
20 - 40th	222 (12.8%)	1.04[0.68,1.62]	0.83	1.02[0.65,1.59]	0.94
40 - 60th	365 (21.0%)	1.14[0.86,1.50]	0.36	1.08[0.82,1.43]	0.59
60 - 80th	355 (20.4%)	1.10[0.79,1.53]	0.56	1.03[0.73,1.46]	0.86
>80th	330 (19.0%)	**1.59[1.15,2.21]**	**<0.01**	**1.53[1.14,2.06]**	**<0.01**

* This table refers to those who reported getting a test from any source.

For peer review

The analysis further focused on those who were tested for malaria by a CHW **(Table 2).** Out of 948 participants who reported having a test, 38.4% reported having taken a test from a CHW (364/948). In 76% of the cases tested by the CHW, the participant reported contacting the CHW as the first (n=277/364) action taken for illness while 11% reported taking drugs at home and then contacting the CHW. It is noted that wealthier participants prefer being tested at the health facility compared to the least wealthy households (Adj. OR for testing at a CHW=0.32, 95%CI:0.17-0.62), and those who completed secondary school have lower odds of taking a test at the CHW although this comparison did not reach statistical significance in the adjusted model

For peer review only - http://bmjopen.bmj.com/site/about/guidelines.xhtml

315 (Adj. OR=0.68, 95%CI:0.44-1.07). On the other hand, school-aged children between 5-17 years
316 were more than twice as likely to be tested by a CHW compared to those below 5 years old
317 (Adj. OR=2.39, 95%CI: 1.43-4.01).

318 **Table 2. Association between patient characteristics and having an mRDT from a CHW**
319 **before taking any treatment.** In this analysis, awareness of a CHW in the village was not
320 included as a covariate because 100% of individuals who had a test by a CHW were also aware
321 of the CHW in the village. Of note, 73.5% of those tested elsewhere were also aware of a CHW
322 in their village.

	**Univariate analysis**		**Multivariate analysis**	
Participant Characteristics of those who took a test from CHW (n=948)	Unadjusted odds ratio (95%CI)	P-value	Adjusted odds ratio (95%CI)	P-value
Time to malaria test (*Reference=same day*)			-	-
Next day	0.96[0.71,1.31]	0.80	0.96[0.69,1.33]	0.81
The second day	0.73[0.44,1.21]	0.22	0.68[0.42,1.10]	0.12
More than two days	0.81[0.49,1.34]	0.41	0.87[0.50,1.52]	0.62
Use an ITN *(Reference=does not use an ITN)*	**0.57[0.41,0.78]**	**<0.01**	0.77[0.55,1.06]	0.11
Distance to public health facility>2km (*Reference = <2km*)	1.32[0.80,2.20]	0.28	1.29[0.82,2.03]	0.27
Patient age (*Reference=1-4y*)			-	-
5 to 17y	**2.43[1.49,3.95]**	**<0.01**	**2.39[1.43,4.01]**	**<0.01**
18+y	0.98[0.67,1.43]	0.91	1.09[0.72,1.66]	0.68
Patient is male (*Reference = female*)	1.23[0.98,1.54]	0.07	1.02[0.82,1.26]	0.85
Respondent education level (*Reference=none*)			-	-
Completed primary	0.86[0.69,1.09]	0.22	0.96[0.75,1.22]	0.74
Completed secondary	**0.45[0.29,0.68]**	**<0.01**	0.68[0.44,1.07]	0.10
Wealth quintile (*Reference=0-20th*)			-	-
20 - 40th	1.13[0.65,1.94]	0.671	1.11[0.63,1.96]	0.72

For peer review only - http://bmjopen.bmj.com/site/about/guidelines.xhtml

40 - 60th	0.83[0.63,1.09]	0.175	0.79[0.58,1.05]	0.11
60 - 80th	**0.67[0.47,0.96]**	**0.028**	**0.66[0.45,0.97]**	**0.03**
>80th	**0.26[0.13,0.53]**	**<0.001**	**0.32[0.17,0.62]**	**<0.01**

This table refers to those who reported getting a test from a CHW.

To understand how individual perceptions about malaria testing and treatment, malaria risk, and illness severity affected uptake of testing or testing at a CHW, the analysis focused on the final survey at 18 months survey **(Table 3)**. After adjusting for demographic characteristics, patient perceptions were differentially related to testing overall and testing specifically with a CHW. Both confidence in AL treatment (Adj. OR=2.75, 95% CI: .1.54-4.92) and confidence in the accuracy of an mRDT performed by a CHW (Adj. OR=2.43, 95% CI: 1.12-5.27) were strongly positively associated with testing at a CHW. Those who reported their illness as severe were more likely to be tested (Adj. OR=2.37, 95% CI: 1.58-3.58) but had a lower odd of testing at a CHW (Adj. OR=0.44, 95% CI: 0.22-0.87). Confidence in an mRDT result, either positive or negative was not significantly associated with receiving a test for a recent illness, but those who reported a high confidence in a negative mRDT result had substantially higher odds of testing with a CHW than in a facility, although this did not reach statistical significance at the 95% level (Adj OR=1.58, 95%CI: 0.89-2.83). Finally, those who reported a moderate perceived prevalence in their village (ie 4-7 fevers out of 10 would be due to malaria) or high perceived prevalence (ie 8-10 out of 10 fevers would be due to malaria) had increasingly higher odds of receiving a test from a CHW (medium prevalence Adj. OR=2.52, 95%CI: 1.26-5.06, high prevalence Adj. OR=2.70, 95% CI: 1.17-6.22).

For peer review only - http://bmjopen.bmj.com/site/about/guidelines.xhtml

Table 3. Relationship between participant beliefs and perceptions and uptake of testing from any source or from a CHW. Models are adjusted for patient age, gender, household wealth quintile and the education level of the respondent in addition to the variables in the table.

	**Univariate analysis**		**Multivariate analysis**	
**Had a malaria test (N=703)**	Unadjusted odds ratio (95%CI)	P-value	Adjusted odds ratio (95%CI)	P-value
Confidence in a positive mRDT result	1.02[0.67,1.54]	0.93	0.87[0.53,1.41]	0.57
Confidence in a negative mRDT result	1.34[0.87,2.03]	0.19	1.24[0.72,2.14]	0.43
Confidence in AL treatment	1.27[0.94,1.71]	0.12	1.06[0.81,1.40]	0.63
Trust an mRDT by a CHW as much as one at a facility	0.85[0.55,1.32]	0.48	**0.66[0.44,0.97]**	**0.03**
Illness is severe	**2.36[1.64,3.39]**	**<0.001**	**2.37[1.58,3.58]**	**<0.001**
Prevalence (*Reference= low*)			-	-
Medium	1.51[0.89,2.57]	0.13	1.48[0.85,2.58]	0.16
High	1.12[0.70,1.80]	0.64	1.05[0.63,1.77]	0.85
	Univariate analysis		Multivariate analysis	
**Had a test with a CHW (N=428)**	Unadjusted Odds Ratio (95%CI)	P-value	Adjusted Odds Ratio (95%CI)	P-value
Confidence in a positive mRDT result	1.45[0.89,2.34]	0.13	1.04[0.64,1.71]	0.86
Confidence in a negative mRDT result	1.61[0.99,2.64]	0.06	1.58[0.89,2.83]	0.12
Confidence in AL treatment	**2.67[1.52,4.70]**	**<0.01**	**2.75[1.54,4.92]**	**<0.01**
Trust an mRDT by a CHW as much as one at a facility	**2.91[1.29,6.58]**	**0.01**	**2.43[1.12,5.27]**	**0.03**
Illness is severe	0.60[0.35,1.03]	0.06	**0.44[0.22,0.87]**	**0.01**
Prevalence (*Reference= low*)			-	-
Medium	**2.87[1.48,5.56]**	**<0.01**	**2.52[1.26,5.06]**	**0.01**
High	**2.85[1.31,6.23]**	**0.01**	**2.70[1.17,6.22]**	**0.02**
Confidence in a positive mRDT result	1.45[0.89,2.34]	0.13	1.04[0.64,1.71]	0.86

For peer review only - http://bmjopen.bmj.com/site/about/guidelines.xhtml

Diagnostic testing by CHWs

In total, 32,404 mRDTs were conducted by the CHWs over the intervention period and 33.7% (n= 10,870) percent were positive. Those with a positive test received a voucher for a discounted quality-assured AL. These vouchers were redeemed at a participating outlet by 93.9% of voucher recipients. There was no instances of stock out of mRDTs among our CHWs. To explore factors that may be associated with the CHWs ability to conduct mRDTs, we examined the association between testing volume, defined as the mean number of mRDTs conducted per month, and the different CHW demographic characteristics (**Table 4**). Of the 244 trained CHWs with complete data, 147 (60.3%) were above 40 years of age, 72 (29.5%) were male. There were 107 (43.8%) CHWs who had not completed secondary education.

Table 4. Association between mean number of tests performed per month and CHW characteristics. 272 CHWs were trained, of which 28 were missing one or more of marital status, employment status, previous RDT training, or education level. The 244 with complete data are reported here.

Mean number of tests per month	4.60 (SD=3.07)	**Univariate analysis**		**Multivariate analysis**	
		**N = 244**		**N = 244**	
CHW characteristics	n (sample proportion)	Coeff (95%CI)	P-value	Coeff (95%CI)	P-value
Male	72 (0.295)	-0.30 [-1.28,0.68]	0.52	-0.28 [-1.24,0.68]	0.54
Age<br><40 years *(Reference)*	97 (0.397)	-	-	-	-
> = 40 years	147 (0.603)	-0.21 [-1.06,0.63]	0.60	-0.42 [-1.12,0.28]	0.23
Married<br>*(Reference = unmarried/divorced)*	202 (0.828)	0.37 [-0.57,1.30]	0.42	0.22 [-0.92,1.35]	0.69
Education<br>Below secondary (*Reference*)	107 (0.438)	-	-	-	-

For peer review only - http://bmjopen.bmj.com/site/about/guidelines.xhtml

Completed Secondary	137 (0.562)	0.45 [-0.33,1.23]	0.24	0.23 [-0.40,0.87]	0.44
Formal Employment *(Reference = no formal employment)*	33 (0.135)	**1.13 [0.01,2.28]**	**0.05**	**1.37 [0.05,2.70]**	**0.04**
Previously trained in mRDT	46 (0.188)	-0.46 [-1.64,0.72]	0.42	**-1.40 [-2.44, -0.37]**	**0.01**
Households[1] <50 *(Reference)*	134 (0.549)	-	-	-	-
50-100	66 (0.271)	**2.14[0.63,3.64]**	**<0.01**	**1.73[0.70,2.74]**	**<0.01**
>100	44 (0.180)	1.18[-0.40,2.76]	0.13	**1.49[0.74,2.24]**	**<0.01**
Cluster-level test positivity[2] Low (≦25%; *Reference)*	141 (0.588)	-	-	**-**	**-**
High (>25%)	103 (0.422)	**2.54[0.97,4.10]**	**<0.01**	**2.14[1.05,3.22]**	**<0.01**

[1] Number of households for which the CHW reported being responsible.

[2] Percentage of mRDTs conducted by the CHWs in that cluster which turned positive over the study period.

In a multivariate analysis, formal employment, previous mRDT training, number of households and mRDT-positivity rate at the cluster-level were associated with volume of tests done. Sex, age, being married and education level were not significantly associated with testing volume. CHWs who were formally employed in some other capacity alongside their CHW voluntary work performed on average 1.37 more tests per month (Adj coefficient=1.37, 95% CI: CI: 0.05-2.70) but those who reported being trained previously on mRDTs performed 1.4 fewer tests per month (Adj coefficient=1.4, 95%CI: -2.44, -0.37). CHWs serving areas with a high proportion of positive tests (proportion mRDT +ve >25%) tested on average 2 more clients per month than those in lower prevalence areas (Adj coefficient= 2.14, 95%CI: 1.05-3.22). CHWs who were responsible for at least 50 households tested more clients (Adj coefficient=1.73, 95%CI: 0.70-2.74), although this did not increase further with when the number of households exceeded 100 (Adj coefficient = 1.49, 95%CI: 0.74-2.24).

For peer review only - http://bmjopen.bmj.com/site/about/guidelines.xhtml

Univariate and multivariate analyses were conducted to test the association between testing volume and CHW perceptions about their role at the midpoint of the intervention period (**Table 5**). High client score was significantly associated with higher testing volume, indicating that more tests were performed by CHWs who perceived that their clients trusted their tests (Adj. coefficient=1.37, 95%CI: 0.11-2.62). The CHWs competency at conducting all 20 steps of the mRDT process (mRDT score), the number of different activities to which CHWs were committed, whether they named mRDT testing as their most important activity, or whether they cited extrinsic motivation (money, airtime, or other) as important reasons for continuing their work were all not significantly associated with average tests performed per month.

Table 5. Association between mean number of tests per month and CHW perceptions about their role at the midpoint survey. Multivariate odds ratios are adjusted for age, gender, education level, formal employment. The analysis includes a random sample of CHWs selected for midpoint evaluation.

	n (sample proportion)	Univariate analysis		Multivariate analysis	
CHW characteristics	N=70	Coeff (95%CI)	P-value	Coeff (95%CI)	P-value
mRDT Score[1] (*Reference=Low*)	-	-	-	-	-
High	54 (0.771)	0.19[-1.25,1.64]	0.78	0.11[-1.34,1.57]	0.87
Number of different activities last month (*Reference*=<=3)	-	-	-	-	-
> 3 activities	19 (0.271)	0.35[-1.56,2.26]	0.70	-0.45[-2.04,1.15]	0.56
Most important activity is malaria testing[2]	48 (0.686)	0.88[-0.25,2.01]	0.12	0.07[-1.16,1.32]	0.90
Cited extrinsic motivators as important[3]	28 (0.400)	0.33[-1.52,2.17]	0.71	0.67[-0.92,2.25]	0.38
Client Score[4] (*Reference= Low*)	-	-	-	-	-
High	48 (0.686)	**1.46[0.10,2.83]**	**0.04**	**1.37[0.11,2.62]**	**0.04**
Years of experience as a CHW (*Reference = <=5y*)	-	-	-	-	-
>5 years	38 (0.543)	-0.35[-2.68,1.98]	0.75	-0.51[-2.56,1.55]	0.61

For peer review only - http://bmjopen.bmj.com/site/about/guidelines.xhtml

[1]mRDT score - CHW scored > 17 correct steps on a 20-step checklist for mRDT preparation and interpretation conducted 6 months after training.
[2]Equals one if malaria testing was cited as their most important activity or 0 if they cited any other activity as most important.

[3] Equals 1 if the CHW cited money, or 0 if the CHW only cited non-monetary incentives or desire for recognition as important motivators for their role as a CHW.
[4] Client score was derived from four questions where the CHW reported how confident clients were in the testing they provided.

Discussion

This study adds to the evidence that it is feasible for CHWs to offer malaria testing using mRDTs in the community. With the use of CHWs, large-scale malaria testing intervention can reach the underserved population who cannot afford to visit the health facilities for care and those who seek care in the retail sector. The existence of CHWs with the ability to test for malaria using mRDT provides a great opportunity, however, various factors may affect the demand and supply of testing using mRDT by CHWs.

Here we examine the outcomes of a community-based malaria testing to understand how to improve intervention design and maximize the impact of such programs. The analysis was organized around the target group (community members) and their uptake or demand for the diagnostic testing intervention, and the supply of testing by the CHWs responsible for delivering the intervention. We were able to separate the determinants of uptake of malaria diagnostic testing generally from the determinants of uptake of malaria rapid diagnostic test performed by a CHW. These contrasting but complementary analyses helped us identify bottlenecks to intervention coverage at the community level.

For peer review only - http://bmjopen.bmj.com/site/about/guidelines.xhtml

Analysis of community survey data revealed that demand for malaria diagnostic testing before treating a fever was lower among adults and is positively associated with being in the highest wealth quintile (relative to the poorest) , findings which are consistent with other published studies.[14-17] We also find that being aware of the local CHW increased demand for testing from any source. This is similar to other studies that have evaluated the contribution of CHWs to population health-seeking behavior.[16,18] In the context of our program, this is especially important because the testing was most often initiated by the community member seeking out a CHW rather than a door-to-door campaign initiated by the CHW. Malaria testing by a CHW provides an opportunity for reducing health disparities by ensuring the poor and underserved population get access to testing and are able to make an informed choice on how to manage their illness. Our results show that community members with a lower socioeconomic score were more likely to receive a test from a CHW compared to those from the highest wealth quintiles. Malaria testing by the CHW was free and this finding therefore aligns well with the intention of the intervention. Testing by a CHW was also more common among those with high confidence in the reliability of a test done by a CHW and also among those with high confidence in artemether-lumefantrine, the treatment offered through the CHW testing program. Taken together, these results suggest that the community awareness of and perception of the CHW is key in improving demand. In contrast, although testing was high among those who considered their illness to be very severe, these individuals were less likely to be tested by a CHW. This result is appropriate and consistent with the goals of the program which included immediate referral of more severe cases.

From the perspective of CHWs delivering malaria testing, a strong association was not observed between demographic characteristics and testing volume, but we did note that CHWs with concurrent formal employment tested more patients on average than those without which could indicate greater visibility or mobility in the community. It was also observed that the monthly testing

volume increased when the number of households under a CHWs purview exceeded 50, but we could not detect a significant increase beyond that when households exceeded 100, suggesting a threshold effect whereby more households created more demand for testing but the ability to meet the demand may be undermined by time constraints after some threshold beyond 100 households. Finally, we report that testing rates were higher in clusters with high mRDT-positivity rates, possible reflecting a greater awareness of malarial illness or higher demand for malaria services in areas with higher malaria burden. This could also reflect higher confidence in a test among CHWs or communities members who have experience with some tests turning positive as has been seen in the healthcare setting. [19]

A deeper analysis of CHW perception of the intervention was undertaken in a smaller random sample of participating CHWs. We show that testing volume is not affected by competing CHW-related priorities, but is associated with how they perceive their clients' confidence in testing and the likelihood of the client accepting the mRDT result and advice. This aligns well with the picture from the demand-side where we observe that confidence in an mRDT performed by a CHW is strongly associated to testing with a CHW.

This study had several limitations. First, we are not able to estimate the effect of free testing on the uptake of testing. Considering the study utilized diagnosis dependent subsidies to test the rational use of AL, a similar study that deploys free testing in the absence of a conditional voucher would be required to resolve this. Similarly, it could be important to compare the effect of an intervention that delivered both the test and the treatment through the CHW rather than using a voucher redeemable in the private sector. Second, although the survey data has provided valuable insight on understanding drivers to demand and supply of malaria testing, seeking provider (CHW) and patient perspectives in a qualitative framework could provide a richer

For peer review only - http://bmjopen.bmj.com/site/about/guidelines.xhtml

understanding of the facilitating factors and barriers to scaling up such an intervention, particularly the ways in which preferences and changes in behavior may influence uptake of the intervention. Finally, though our survey design used repeated cross-sectional sampling, it is possible that the same household and/or febrile individual was surveyed at multiple survey time points and any resulting association was not explicitly modeled in our regression analysis. Our results are based on a representative, randomly-selected population sample of recent fevers. As a result, our findings could be generalized to other contexts or service delivery programs working through CHWs in communities where care for febrile illness is provided through a heterogeneous landscape of private, public and retail venues.

Conclusion

In summary, scale up of health interventions through CHWs is feasible and effective at reaching the poorest households. However, in order to maximize the impact of community-based interventions, it is important to recognize factors that may restrict both delivery and demand for such services. It is critical to not only create awareness of CHW services but foster trust in both the CHWs ability to deliver services and the health intervention itself. Engendering confidence in the CHWs proficiency is likely to have a synergistic effect on both demand for services and commitment to delivering services.

Declarations

Ethics approval and consent to participate: Ethical approval was granted by Moi University Institutional Research and Ethics Committee (*Formal Approval No. 0001403*) and Duke University Institutional Review Board (*Pro00063384*). Written informed consent was obtained from

For peer review only - http://bmjopen.bmj.com/site/about/guidelines.xhtml

participants during household interviews and from CHWs prior to collecting midpoint evaluation data. All study methods were carried out in accordance with ethical guidelines and regulations set out by both Moi University and Duke Ethical Review Boards.

Consent for publication: Not applicable.

Data availability statement: The datasets used and/or analysed during the current study are available from the corresponding author on reasonable request.

Competing interests: The authors declare that they have no competing interests

Funding: This study was supported from the National Institute of Allergy and Infectious Diseases of the National Institutes of Health (US) (grant number R01AI110478). The funders had no role in study design, data collection and analysis, decision to publish, or preparation of this manuscript. The content is solely the responsibility of the authors and does not necessarily represent the official views of the National Institute of Allergy and Infectious Diseases or the National Institutes of Health.

Contributors:

Joseph Kirui – Conceptualization, writing original draft, revision and editing final draft.

Josephine Malinga – Data curation, writing original draft, revision and editing final draft.

Edna Sang - writing original draft, writing original draft, revision and editing final draft.

George Ambani – Data collection, writing original draft, revision and editing final draft.

Lucy Abel - Data collection, writing original draft, revision and editing final draft.

For peer review only - http://bmjopen.bmj.com/site/about/guidelines.xhtml

Erick Nalianya - Data collection, writing original draft, revision and editing final draft.

Jane Namae - Data collection, writing original draft, revision and editing final draft.

Matthew Boyce - Review and editing final draft.

Jeremiah Laktabai – Conceptualization, review and editing final draft.

Diana Menya - Conceptualization, review and editing final draft.

Wendy Prudhomme-O'Meara – Conceptualization, funding acquisition, writing original draft, revision and editing final draft.

Acknowledgements: This study could not have been carried out without the exceptional attention to detail and commitment of our field team that include: I. Khaoya, L. Marango, E. Mukeli, L. Nukewa, E. Wamalwa, and A. Wekesa. We are grateful to the study participants and the community health workers as well as the local community leadership that supported this work. We would also like to acknowledge the partnership and support of our Health Management Team colleagues in each subcounty and in particular V. Kiplagat, A. Kyalo, S. Malenya, and P. Musita.

Supplemental material

Supplemental file 1: Community survey tool used to collect information about recent fevers among participating households

Supplemental file 2: Survey tool used at the midpoint CHW evaluation

For peer review only

For peer review only - http://bmjopen.bmj.com/site/about/guidelines.xhtml

References

1. World Health Organization. World malaria report 2020: 20 years of global progress and challenges. Geneva; 2020.

2. World Health Organization, Global Malaria Programme. Guidelines for the treatment of malaria. Geneva;. 2015. Available from: http://www.ncbi.nlm.nih.gov/books/NBK294440/ (accessed Jan 20, 2021)

3. World Health Organization. World malaria report 2019. Geneva; 2019.

4. WHO Guidelines for malaria. Geneva: World Health Organization; 2021. Available from: https://www.ncbi.nlm.nih.gov/books/NBK568494/

5. ACTwatch Group, Musuva A, Ejersa W, Kiptui R, Memusi D, Abwao E. The malaria testing and treatment landscape in Kenya: results from a nationally representative survey among the public and private sector in 2016. Malar J. 2017 21;16(1):494.

6. Prudhomme O'Meara W, Menya D, Laktabai J, Platt A, Saran I, Maffioli E, et al. Improving rational use of ACTs through diagnosis-dependent subsidies: Evidence from a cluster-randomised controlled trial in western Kenya. PLoS Med. 2018;15(7):e1002607.

7. Cohen J, Dupas P, Schaner S. Price Subsidies, Diagnostic Tests, and Targeting of Malaria Treatment: Evidence from a Randomised Controlled Trial. Am Econ Rev. 2015 Feb;105(2):609–45.

8. Briggs MA, Kalolella A, Bruxvoort K, Wiegand R, Lopez G, Festo C, et al. Prevalence of Malaria Parasitemia and Purchase of Artemisinin-Based Combination Therapies (ACTs)

For peer review only - http://bmjopen.bmj.com/site/about/guidelines.xhtml

541 among Drug Shop Clients in Two Regions in Tanzania with ACT Subsidies. PLOS ONE. 2014 Apr 14;9(4):e94074.

543 9. Mbonye AK, Lal S, Cundill B, Hansen KS, Clarke S, Magnussen P. Treatment of fevers prior to introducing rapid diagnostic tests for malaria in registered drug shops in Uganda. Malar J. 2013 Apr 16;12(1):131.

546 10. World Health Organization, Special Programme for Research and Training in Tropical Diseases, Foundation for Innovative New Diagnostics, Centers for Disease Control (U.S.). Malaria rapid diagnostic test performance: results of WHO product testing of malaria mRDTs : 4 (2012). [Internet]. Geneva, Switzerland: World Health Organization on behalf of the Special Programme for Research and Training in Tropical Diseases; 2012 [cited 2021 Jun 7]. Available from: http://apps.who.int/iris/bitstream/10665/77748/1/9789241504720_eng.pdf

553 11. Laktabai J, Lesser A, Platt A, Maffioli E, Mohanan M, Menya D, et al. Innovative public-private partnership to target subsidised antimalarials: a study protocol for a cluster randomised controlled trial to evaluate a community intervention in Western Kenya. BMJ Open. 2017 20;7(3):e013972.

557

558 12. Winn LK, Lesser A, Menya D, Baumgartner JN, Kipkoech Kirui J, Saran I, et al. Motivation and satisfaction among community health workers administering rapid diagnostic tests for malaria in Western Kenya. J Glob Health. 2018 Jun;8(1):010401.

For peer review only - http://bmjopen.bmj.com/site/about/guidelines.xhtml

13. Burnham KP, Anderson DR, Huyvaert KP. AIC model selection and multimodel inference in behavioral ecology: some background, observations, and comparisons. Behav Ecol Sociobiol. 2011 Jan 1;65(1):23–35.

14. Chibwana AI, Mathanga DP, Chinkhumba J, Campbell CH. Socio-cultural predictors of health-seeking behaviour for febrile under-five children in Mwanza-Neno district, Malawi. Malar J. 2009 Sep 24;8(1):219.

15. Cohen J, Cox A, Dickens W, Maloney K, Lam F, Fink G. Determinants of malaria diagnostic uptake in the retail sector: qualitative analysis from focus groups in Uganda. Malar J. 2015 Dec;14(1):89.

16. Kisia J, Nelima F, Otieno DO, Kiilu K, Emmanuel W, Sohani S, et al. Factors associated with utilization of community health workers in improving access to malaria treatment among children in Kenya. Malar J. 2012 Jul 30;11:248.

17. Mukanga D, Tibenderana JK, Peterson S, Pariyo GW, Kiguli J, Waiswa P, et al. Access, acceptability and utilization of community health workers using diagnostics for case management of fever in Ugandan children: a cross-sectional study. Malar J. 2012 May 24;11(1):121.

18. Win Han Oo, Gold L, Moore K, Agius PA, Fowkes FJI. The impact of community-delivered models of malaria control and elimination: a systematic review. Malar J. 2019 Aug 6;18(1):269.

For peer review only

For peer review only - http://bmjopen.bmj.com/site/about/guidelines.xhtml

19 Faust C, Zelner J, Brasseur P, Vaillant M, Badiane M, Cisse M, et al. Assessing Drivers of Full Adoption of Test and Treat policy for Malaria in Senegal. Am J Trop Med Hyg. 2015 Jul 8;93(1):159–67.

For peer review only

For peer review only - http://bmjopen.bmj.com/site/about/guidelines.xhtml

A. *Household survey instrument* (Note: the survey will be administered electronically)

1. Date of interview (DD/MM/YY):

   ☐☐ / ☐☐ / 20 ☐☐

2. Name of interviewer: ____________

3. Interviewer ID: ☐☐

CU – dropdown menu

Demographics

4. Location: ____________

5. Sub-location: ____________

6. Village name: ____________

Eligibility criteria

7. Did any member of the household have fever or malaria-like illness in the last four weeks? *If no, stop.*
   1. Yes
   2. No
   3. Don't Know

8. How long ago did the most recent fever start?

   ☐☐ Days

INSTRUCTION: if the ill individual is 18 or older and not present, stop and save the interview now; recording the time and date saved on your notebook; return to the saved interview if the ill adult is present on a return visit, use the recorded time and date to locate the form.

9. Are you still ill today?
   1. Yes
   2. No

Patient information

10. Gender of patient:

For peer review only - http://bmjopen.bmj.com/site/about/guidelines.xhtml

1. Male
2. Female

11. Relationship of patient to household head
    1. Head
    2. Spouse of head
    3. Child
    4. Parent
    5. Grandparent
    6. Grandchild
    7. Aunt /Uncle
    8. Brother / Sister
    9. Cousin
    10. Mother
    11. Father
    12. Employee
    13. OTHER____________________

12. Age of patient: [|] Years (if less than 18, skip to respondent information, question 15)

13. Highest school level:
    1. None
    2. Some Primary (not completed Class 8)
    3. Primary completed
    4. Primary completed and some secondary (secondary not completed)
    5. Secondary completed
    6. College
    7. University

14. Occupation
    1. Farming/Livestock keeping
    2. Home maker
    3. Student
    4. Paid employee
    5. Casual employee
    6. Self-employed (not agriculture, livestock) without employees
    7. Self-employed (not agriculture, livestock) with employees
    8. Unpaid family helper in a business
    9. Not working
    10. Unable to work/disabled
    11. Other (Specify)

Respondent (Answer if age of patient is <18 years. If patient is >18 years, skip to question 19.)

15. Age of respondent

For peer review only - http://bmjopen.bmj.com/site/about/guidelines.xhtml

☐☐ Years

16. Highest school level:
    1. None
    2. Some Primary (not completed Class 8)
    3. Primary completed
    4. Primary completed and some secondary (secondary not completed)
    5. Secondary completed
    6. College
    7. University

17. Gender of respondent:
    1. Male
    2. Female

18. Relationship of respondent to child:
    1. Parent
    2. Aunt/Uncle
    3. Grandparent
    4. Sibling
    5. Other

Endpoints *Note: The wording of the questions in this section are directed at a respondent 18 or over; when administered as an electronic survey, there will be two parallel sets of questions that branch based on whether a child or adult was the patient.*

19. How severe was the illness?
    1. Very severe
    2. Severe
    3. Moderately severe
    4. Mild
    5. Very mild

20. Did you take any action for your illness? *Note: taking drugs at home is an action. If "Don't remember" or "No", skip to COVARIATES section, question 56.)*

    1. Yes
    2. No
    3. Don't remember

21. What did you do FIRST for your illness? *Only one answer*
    1. Contact CHW

For peer review only - http://bmjopen.bmj.com/site/about/guidelines.xhtml

2. Go to a government health centre
3. Go to a government dispensary
4. Go to a government hospital
5. Go to a private clinic or mission facility
6. Go to a shop or pharmacy
7. Take some drugs at home
8. visit traditional healers
9. visit religious/cultural healers
10. OTHER: ____________

22. What were the MAIN reasons for deciding to do that? *Multiple answers OK*
    1. I already had the drugs at home
    2. It is the nearest place (facility or drug shop)
    3. The services are free
    4. Money- this was most affordable
    5. Wanted to see a Doctor
    6. Illness very serious
    7. Illness not very serious
    8. Time was limited / this was the fastest
    9. Good quality service
    10. There are no drugs in the facility
    11. There is laboratory testing
    12. OTHER: ____________

23. How long after the illness began did you do that?
    1. same day
    2. next day
    3. after two days
    4. more than two days later
    5. OTHER: ____________

24. Did you take any other action? *If no, skip to question 26.*
    1. Yes
    2. No
    3. Don't remember

25. If yes, what did you do? *Allow multiple answers*

    1. Contact CHW
    2. Go to a government health centre
    3. Go to a government dispensary
    4. Go to a government hospital
    5. Go to a private clinic or mission facility
    6. Go to a shop or pharmacy

For peer review only - http://bmjopen.bmj.com/site/about/guidelines.xhtml

7. Take some drugs at home
8. visit traditional healers
9. visit religious/cultural healers
10. OTHER: ___________

26. How long did you take to fully recover?

[|] Days (99=not yet recovered)

27. Did you have any laboratory test done during your illness? *PROBE. Ask about blood, stool or urine taken to help find out about the illness*

1. Yes
2. No
3. Don't remember

28. Did you have a malaria test? *If no or don't remember, skip to question 42.*

1. Yes
2. No
3. Don't remember
4. Don't know

29. How long after the start of the illness did you have a malaria test (the first test, if more than one)?

1. same day
2. next day
3. after two days
4. OTHER: ___________

30. Was it a microscopy test or a rapid diagnostic test? (If both, record in 'OTHER')

1. RDT
2. microscopy
3. Don't remember or don't know
4. OTHER (specify): _____________

31. Where was the malaria test done?

1. CHW (go to Question 32, else skip to 35.)
2. shop or pharmacy
3. private clinic/hospital
4. government dispensary
5. government hospital
6. government health center
7. private lab
8. OTHER: ___________

For peer review only - http://bmjopen.bmj.com/site/about/guidelines.xhtml

32. If a CHW did the test, where was it done?
    1. In my village
    2. In another village but the same CU (in which the survey is being conducted)
    3. Another CU (write name: ___________)
    4. Don't know/don't remember

33. Did the CHW give you a voucher to purchase a drug?
    1. Yes (Ask question 34 else skip to 35.)
    2. No
    3. Don't know/don't remember

34. Did you use the voucher to purchase a drug?
    1. Yes
    2. No
    3. Don't know/ don't remember

35. How much did the malaria test cost (Ksh.)? If more than one test, list both
    ______________**Free text**

36. What were the results of the malaria test? (If more than one test, use OTHER)
    1. Positive
    2. Negative
    3. Don't remember / Don't know
    4. OTHER________________

37. How likely is it that the result of your malaria test was correct?
    1. Very likely
    2. Likely
    3. 50-50
    4. Unlikely
    5. Very unlikely
    6. Don't know
    7. OTHER (use if have different beliefs about multiple tests)

38. Record observed for test? *Interviewer question (this can include study forms)*
    1. Yes
    2. No (Skip to question 42.)

If yes (record observed), interviewer should answer the next 3 questions:

39. Where does the record report the test was done? (multiple answers possible)

For peer review only - http://bmjopen.bmj.com/site/about/guidelines.xhtml

1. CHW
2. shop or pharmacy
3. private clinic/hospital
4. government dispensary
5. government hospital
6. government health center
7. private lab
8. Not specified / Cannot be determined
9. OTHER: ____________

40. What type of malaria test does the record report was done? (if more than one test, use OTHER)

1. RDT
2. microscopy
3. Not specified / Cannot be determined
4. OTHER (specify): ______________

41. What were the results of the malaria test according to the record? (if more than one test, use OTHER)

1. Positive
2. Negative
3. Not specified / Cannot be determined
4. OTHER ____________________

42. How likely is it that the illness was malaria?

1. Very likely
2. Likely
3. 50-50
4. Unlikely
5. Very unlikely
6. Don't know
7. No response

43. Were any drugs taken for the illness? *If no or don't remember, skip to question 55.*

1. Yes
2. No
3. Don't remember

44. Which medicines did you take? (Multiple answers possible) SKIP PATTERN: If 1 or 4 (ACT), continue with next question. Else, skip to 53

1. Coartem/Artefan/other AL
2. Quinine

For peer review only - http://bmjopen.bmj.com/site/about/guidelines.xhtml

For peer review only

3. SP/Fansidar/Metakelfin/Moladar
4. Other ACT (e.g Duocotexin)
5. Amoxyl
6. Septrin
7. Ciprofloxacin/Norfloxaccin
8. Panadol/Brufen/Hedex/Painkiller/Action/Maramoja
9. Don't know/Don't Remember
10. Other (specify: __________)

45. Packaging observed for ACT? *Interviewer question. If YES, ask question 46. If NO, skip to question 47.*

1. Yes
2. No

46. If ACT package observed, what brand? *Interviewer question*.

1. Coartem (AL)
2. Aretefan (AL)
3. Duocotexin
4. P-Alaxin
5. AL (IPCA brand)
6. AQ AS
7. Lonart (AL)
8. Artequick
9. Lumartem (AL)
10. Combiart (AL)
11. L-artem (AL)
12. OTHER: ___________

47. How long after the start of the illness did you start taking the ACT?

1. same day
2. next day
3. after two days
4. OTHER: ___________

48. Did you start taking the ACT before or after the malaria test? *Only ask this if YES to 28.*

1. Before
2. After
3. Don't remember
4. Not Applicable

If respondent answered "1-Coartem/Artefan/other AL" to question 44, ask questions 49-52, else skip to question 53.

49. If AL, how many pills were given? *Only ask if 44 = 1 (AL ONLY)*

For peer review only - http://bmjopen.bmj.com/site/about/guidelines.xhtml

1. 6
2. 12
3. 18
4. 24
5. Don't know/remember
6. OTHER: ___________

50. If AL, did you / patient take all of the tablets? *Only ask if 44 = 1 (AL ONLY)*

1. Yes
2. No
3. Not sure
4. Don't remember
5. Not yet—still taking them

51. Where was the AL obtained? MULTIPLE RESPONSES POSSIBLE
(If the drugs were already in the home at the onset of illness, record this but find out from where they were originally obtained) Only ask if 44 = 1, 4 ie any ACT.

1. Used drugs at home
2. Pharmacy, chemist or duka la dawa with a prescription
3. Pharmacy, chemist or duka la dawa; no prescription
4. CHW
5. Neighbor
6. Private health facility
7. Government health facility
8. general store
9. OTHER: _______________
10. Don't remember/don't know

52. Did you get AL at the first place you sought treatment? *Only ask if 44 = 1,4 (ACT ONLY)*

1. Yes
2. No
3. Yes and another place
4. Don't know/don't remember

53. How likely do you think the malaria drugs you took were effective? (ask if respondent answered 1, 2, 3 or 4 to question 44.)

1. Very likely
2. Likely
3. 50-50
4. Unlikely

For peer review only - http://bmjopen.bmj.com/site/about/guidelines.xhtml

5. Very unlikely
6. Don't know
7. No response

54. How much did you spend for all the drugs you purchased for this illness? (-1 should be used for 'I don't know/don't remember')

1. ________________________

55. How much did you spend in total to take care of this illness, including testing, drugs, transportation, admission fees, registration and consultation fees? (-1 should be used for 'I don't know/don't remember')

1. _________________________

For peer review only

Covariates *THESE QUESTIONS DIRECTED AT RESPONDENT, EVEN IF NOT THE PATIENT*

56. GPS coordinates: ____ (PLACED AT THE END OF THE ELECTRONIC SURVEY)

57. Does your household have any mosquito nets? (*If no or don't know, skip to question 60.*)

1. Yes
2. No
3. DK

58. Did THE PATIENT sleep under the net last night?

1. Yes
2. Has a net for sleeping space, but did not use it last night.
3. Does not have net for sleeping space
4. Do not Remember/Don't Know

59. Was the net pre-treated with insecticide when you got it?

1. Yes
2. No
3. Not Applicable (no net for sleeping space)
4. Do not Remember/Don't Know

60. Is there a CHW in your village that provides any services or health education? *If no or do not know skip to question 66.*

1. Yes
2. No
3. Don't Know

61. Which services does the CHW provide? *Multiple responses possible*

1. Information

For peer review only - http://bmjopen.bmj.com/site/about/guidelines.xhtml

2. Referral
3. Polio immunization
4. Advice
5. Testing for HIV
6. Testing for malaria
7. Testing for diabetes
8. Testing for hypertension
9. Testing for pregnancy
10. Coupon for antimalarial drug
11. Bednets
12. Water treatment (ie Lifestraw)
13. Other (specify) ____________________

62. Does your CHW provide malaria diagnostic testing?
1. Yes
2. No
3. Don't know/Don't remember

63. Does your CHW provide a discount on a malaria drug in case the test is positive for malaria?
1. Yes
2. No
3. Don't know/Don't remember

64. Have you ever contacted a CHW near you in case of illness for yourself or someone in your household?
1. Yes
2. No (*skip to question 66*)
3. DR/DK (*skip to question 66*)

65. What did the CHW do? (multiple responses possible)
1. Refer
2. Give medicine
3. Accompany to shop
4. Give advice
5. Perform a malaria test
6. Give a coupon for an antimalarial drug
7. Other (specify)________________

66. Do you know what a rapid diagnostic test for malaria is? *Show example of cassette*
1. Yes
2. No → skip to 71

67. Have you ever had an RDT test for malaria?
1. Yes
2. No
3. Don't know/don't remember

68. Do you know anyone besides one of your household members who has had an RDT?
1. Yes
2. No

For peer review only - http://bmjopen.bmj.com/site/about/guidelines.xhtml

3. Don't know/don't remember

69. If you have fever and your malaria RDT is negative, how likely is it that the test is correct?
    1. Very likely
    2. Likely
    3. 50-50
    4. Unlikely
    5. Very unlikely
    6. Don't know
    7. No response

70. If you have fever and your malaria RDT is positive, how likely is it that the test is correct?
    1. Very likely
    2. Likely
    3. 50-50
    4. Unlikely
    5. Very unlikely
    6. Don't know
    7. No response
71. Is malaria common in your village
    1. Yes
    2. No
    3. Don't know/not sure
72. If ten people (watu) from your village have fever, how many of them are likely to have malaria? _____________(number 0-10 or 99 for don't know)
73. If you have malaria, and you take AL, how likely is it that you will be completely better in 3 days?
    1. Very likely
    2. Likely
    3. 50-50
    4. Unlikely
    5. Very unlikely
    6. Don't know
    7. No response

SES Questions
(Source:pilot)

74. Does your household have the following items
    1. Electricity
    2. Television
    3. Refrigerator
    4. Radio

For peer review only - http://bmjopen.bmj.com/site/about/guidelines.xhtml

5. Mobile phone
6. Motorcycle
7. car/truck
8. Bank account
9. None

75. How many of the following livestock does your household have
    1. Cows
    2. Sheep
    3. Goats
    4. Pigs
    5. Donkeys

76. What kind of toilet does your household have
    1. Flush or pour flush toilet
    2. VIP/Ventilated improved pit
    3. Pit latrine WITH slab
    4. Pit latrine without slab
    5. Composting toilet
    6. Bucket toilet
    7. No facility/bush/field
    8. Other

77. Main source of drinking water for your household
    1. Piped water/public tap/borehole
    2. Unprotected well
    3. Protected well
    4. Protected spring
    5. Unprotected spring
    6. Rain Water
    7. River Water
    8. Other:______________

78. Main material of the floor in your house
    1. Earthen
    2. Cement
    3. Floor tiles
    4. Wood planks
    5. Polished wood
    6. Other

79. Main material of the walls in your house
    1. Stone
    2. Brick
    3. Timber
    4. Iron Sheet
    5. Mud

For peer review only

For peer review only - http://bmjopen.bmj.com/site/about/guidelines.xhtml

6. Wood
7. Cement
8. Other

80. Main material of the roof of your house
    1. Iron sheets
    2. Roof tiles
    3. Grass thatched
    4. Wood

81. How many acres/hectares/feet of land for farming does your household own
    1. None
    2. Acres: ___________
    3. Hectares: _______
    4. Square feet(xx by xx):_________

For peer review only - http://bmjopen.bmj.com/site/about/guidelines.xhtml

Coupon R01 CHV Participation Assessment

April 13, 2016

Note on administration: This survey is intended to be administered orally by an enumerator to a CHV.

I. Background Information

1) Sub-county:
    1. Bungoma East
    2. Kiminini

2) Name of CU that the CHV serves: ________________________

3) CHV's gender:
    1. Male
    2. Female

4) Age: ________ Years

5) Highest school level:
    1. None
    2. Some Primary (not completed Class 8)
    3. Primary completed
    4. Primary completed and some secondary (secondary not completed)
    5. Secondary completed
    6. College
    7. University

6) For how long have you served as a CHV?
    1. < 1
    2. > 1-3 Years
    3. > 3-5 Years
    4. > 5 Years

7) Apart from serving as a CHV, what other occupation(s) or role(s) do you have?

For peer review only

For peer review only - http://bmjopen.bmj.com/site/about/guidelines.xhtml

1. *Write-in response:* ________________________
2. None

8) How many trainings for CHV activities of any kind have you had since the RDT training for this malaria project?
    1. None
    2. One
    3. Two
    4. Three
    5. Four
    6. Five
    7. More than five

For peer review only

9) How long ago was your most recent training for CHV activities of any kind?
    1. Within the past week
    2. Between one week and one month ago
    3. Between one and three months ago
    4. Between three months and 1 year ago
    5. More than one year ago

10) What was the topic of your most recent training?
    1. Malaria (this project)
    2. Malaria (another project)
    3. Maternal health
    4. Polio campaign
    5. Other vaccination program
    6. Other child health topic not mentioned above

    7. Other: _______

II. Self-description of Role

11) In the past month, what activities have you carried out as a CHV overall?
(Enumerator: check all that are mentioned; do not read the list)
    1. Malaria testing
    2. Provide vouchers for malaria drugs
    3. Mosquito net distribution
    4. Provide other malaria-related services/counseling

5. Polio campaign participation
6. Other vaccination campaign(s)
7. Child health services not mentioned above
8. Maternal health services not mentioned above
9. Giving health education to households
10. None
11. Other: ____________________________

____

____

12) How many times have you met with your CHEW in the past 3 months?
1. Once
2. Twice
3. Three times
4. More than three times
5. None

13) What activities do you perform specifically for the malaria project?
(Enumerator: check all that are mentioned; do not read the list)
1. Perform malaria RDTs
2. Provide vouchers for malaria drugs
3. Provide malaria drugs
4. Provide referrals
5. Provide counseling / advice
6. Other: ____________________________________

14) For how many clients have you performed an RDT in the past month?
1. 1 client
2. 2-5 clients
3. 5 clients and above

15) What are the requirements for a client to receive an RDT under this malaria project?
(Enumerator: check all that are mentioned; do not read the list)
1. No requirements – anyone who asks can be tested
2. Must live in my CU
3. Has a documented fever (i.e., as measured by me)

For peer review only

4. Has a documented fever or reports a history of recent fever
5. Has a documented fever or reports a history of recent fever or reports any symptoms of malaria
6. Consent must be provided by the client (or by the parent/guardian of a minor)
7. Must not be visibly pregnant

8. Other: ___________________________________

16) What motivates you to participate in this malaria project?
1. Personal desire
2. Previous experience
3. Family
4. Organization (partner and GOK)
5. Monetary incentive (money given to group after 6 months and transport to attend supervision and supplies replenishment)
6. In-kind incentive (e.g., airtime for your phone)
7. Support from CHEWs
8. Support from link facilities in attending to clients referred for care
9. Other:____________________________

III. Satisfaction with Role

17) What are the bad things about participating in this project?
(Enumerator: check all that are mentioned; do not read the list)

(Open-ended response): ____

____

____

18) What are the good things about participating in this project?
(Enumerator: check all that are mentioned; do not read the list)

(Open-ended response): ____

____

Please indicate your level of agreement with the following statements with regards to the malaria project **only.**

Question	Strongly Agree	Somewhat Agree	50-50	Somewhat Disagree	Strongly Disagree
19) Overall, I am confident in my ability to perform my role in this malaria project.					
20) I receive enough supervision in my work from the malaria project study staff.					
21) I believe the clients that test positive for malaria follow the advice that I give them					
22) I believe the clients that test negative for malaria follow the advice that I give them					
23) The people in my community appreciate my work on the malaria project.					
24) I have gained more recognition in my community since I began testing for malaria.					
25) I am proud of the work that I do in the malaria project.					
26) I have gained valuable knowledge as a result of my role in this malaria project.					
27) Overall, I think the malaria project is an important activity for me to be involved in.					
28) Overall, I enjoy carrying out my role in this malaria project.					
29) I would like to continue with my role in this malaria project.					

For peer review only

30) *If the CHV answers "50-50", somewhat disagree" or "slightly disagree" to continuing their role in the project*, why?
(Enumerator: check all that are mentioned; do not read the list)

1. Requires too much time
2. Requires too much effort
3. Don't receive enough appreciation
4. Don't receive compensation
5. Frustrated that they can't provide drugs directly to clients
6. Conflicts with other CHV activities
7. Conflicts with non-CHV activities / responsibilities
8. Other ____

IV. Logistics & Challenges of Role

A. Implementation

i. Community awareness & trust of services

Question	Always aware	Usually aware	Sometimes aware	Rarely aware	Never aware
31) Of all of the clients that you provide services to for any reason, how often are the clients already aware that you provide RDT testing?					
32) Of the clients that visit you with a fever or other symptoms of malaria, how often are these clients already aware that you provide RDT testing?					
33) Of the clients that visit you with a fever or other symptoms of malaria, how often are these clients already aware that you provide vouchers for malaria drugs?					

34) How do you think your clients learn about the malaria services (testing and vouchers) that you provide?
(Enumerator: check all that are mentioned; do not read the list)

1. From me
2. From family members
3. From neighbors / friends
4. From village leaders
5. From health facility staff
6. From malaria project staff

7. Other: ___________________________

For peer review only

Please indicate your level of agreement with the following statements.

Question	Strongly Agree	Somewhat Agree	50-50	Somewhat Disagree	Strongly Disagree
35) The malaria RDT that I do can be trusted just as much as a malaria RDT done in a health facility.					
36) The clients that test positive for malaria trust the results of the test					
37) The clients that test negative for malaria trust the results of the test					
38) People I test for malaria believe I am actually testing for HIV.					

39) Do any of your clients refuse an RDT when you offer it?
1. Yes
2. No

40) *If Yes to above,* What are the reasons that you think these clients refuse?
(Enumerator: check all that are mentioned; do not read the list)
3. They don't think it's necessary
4. They don't trust me
5. They don't trust the test
6. They are already convinced they have malaria
7. They plan to buy antimalarial drugs regardless of test result
8. They don't want to be pricked
9. They think it may really be an HIV test

10. Other: ______________________

ii. Logistics & Challenges

41) What are all of the ways that clients find you to receive a malaria RDT?
(Enumerator: check all that are mentioned; do not read the list)
1. Clients visit me at my home
2. Clients visit me at a health facility

For peer review only

3. Clients visit me at church
4. I visit clients at their homes
5. Client visit me at another public location
6. Client visit me at another private location
7. Clients call my mobile phone
8. The study staff contacts me
9. The health facility contacts me

10. Other:______________________

42) Where do you provide testing for malaria? *(Enumerator: check all that are mentioned; do not read the list)*
1. At my home
2. At a health facility
3. At church
4. At client's home
5. At another public location
6. At another private location
7. At CHV Office
8. In Church
9. In Barazas (public meeting)

10. Other:______________________

43) How often do you lack all of the supplies you need to perform the malaria services of testing and providing vouchers when necessary?
1. Always
2. Sometimes
3. 50-50
4. Rarely
5. Never

44) Which, if any, supplies related to the malaria services you perform for this project have you had a stock-out of in the past month?
(Enumerator: check all that are mentioned; do not read the list)
1. RDTs
2. Buffer
3. Lancets
4. Alcohol swabs
5. Cotton wool
6. Gloves
7. Safety disposal box
8. Yellow waste bags
9. Plastic (zip-loc) bags
10. Thermometer
11. Thermometer batteries

For peer review only

12. Pen
13. Encounter forms
14. Voucher forms

15. Other:____________________________

45) How would prefer your consumption of malaria project supplies be monitored?
1. CHV counts the supplies physically
2. CHV references filled encounter forms
3. CHV uses inventory card to document consumption of supplies
4. CHV relies on the supervision team to monitor the supplies

46) What are the main challenges and barriers you face in implementing the malaria project? *(Enumerator: check all that are mentioned; do not read the list)*
1. Community is unaware that I offer malaria RDTs
2. Community is unaware that I offer vouchers for malaria drugs
3. The clients don't always trust the test results I give them
4. The clients are unhappy that I can't provide the malaria drugs to them directly
5. Transport
6. Not enough training
7. Not enough supervision from study staff
8. Not enough supervision from CHEWs
9. Lack of inventory systems to monitor supplies
10. Competing activities within the community
11. Other roles by other NGOs interfere with Malaria project
12. My other main occupation does not give me time to participate in Malaria project
13. Very few clients coming for test
14. Nature of the community not willing to interact with CHVs
15. Cultural practices e.g use of herbal medicine, seeking help from traditional healers
16. Level of education of community member (the less educated don't understand the importance of malaria test and the very educated not willing to be tested by the community health volunteer
17. Lack of mobilization at the community
18. Family relationships are affected
19. Other: ___________________________________

47) Does frequent supervision improve your ability to provide quality malaria testing?
1. Yes
2. No

48) *If yes above*, how frequently would you prefer supervision to be carried out?
1. Once a week
2. Once every two weeks
3. Once a month
4. Once every two months

49) What is the main purpose of supervision activities conducted by the malaria project team?
(Enumerator: check only one)
    1. To monitor whether I make any mistakes
    2. To provide additional training
    3. To ensure data quality

50) What would help you perform your role in the malaria project better?
(Enumerator: check all that are mentioned; do not read the list)
    1. More training
    2. More supervision from study team
    3. More supervision from CHEWs
    4. More regular restocking of supplies
    5. Raising community awareness about the project
    6. Compensation for time spent
    7. Sharing my CHV duties with another CHV
    8. Other: ________________________________

iii. Time required

51) On average, how long does it take you to complete a visit with a patient complaining of malaria symptoms?

    ___________________ Minutes

52) In the last week, what portion of your CHV activities have been related to the malaria project?
    1. Less than half
    2. About half
    3. More than half

53) How many hours do you think you spend on the malaria project activities in an average week (including seeing clients, filling forms, meeting with malaria study staff, etc.)?

    ___________________ Hours

54) How many hours do you think you spend on all of your other CHV activities and responsibilities in an average week (including seeing clients, reporting, restocking, trainings, etc.)?

    ___________________ Hours

55) Do you think the total time you spend on all CHV activities has decreased, increased, or stayed about the same since you began the malaria project activities?

For peer review only

1. Decreased
2. Stayed about the same
3. Increased

B. <u>Relation to other CHV activities</u>

56) Has participating in the malaria project had an effect on your other CHV activities?
1. Yes
2. No

57) If yes, in what ways:
(Enumerator: check all that are mentioned; do not read the list)
1. Improved skills applicable to other CHV activities
2. More clients visit me for non-malaria services
3. Less time to complete other CHV activities

4. Other: ____________________

58) Please indicate to what extent you agree with the statement: Due to my involvement with the malaria project, I now provide more non-malaria health services than before the malaria project.
1. Strongly agree – I provide much more non-malaria health services than before
2. Somewhat agree
3. Neither agree nor disagree – it has stayed the same
4. Somewhat disagree
5. Strongly disagree – I provide much less non-malaria health services than before

(Only ask the next 2 questions if "strongly agree" or "somewhat agree" above):

59) If "strongly agree" or "somewhat agree" above: What are the other (non-malaria) services you provide more of?
(Enumerator: check all that are mentioned; do not read the list)
1. Maternal health services
2. Vaccinations
3. Providing health information
4. *… (Other typical CHV activities to include in this list?)*

5. Other: ___________________________

60) To whom do you provide more non-malaria services than before the malaria project started? (select all that apply):
(Enumerator: check all that are mentioned; do not read the list)
1. Men
2. Women

For peer review only

3. Children

61) Please indicate to what extent you agree with the statement: There are other CHV activities or services I would normally have performed in the last month that I did not because the malaria project took too much of my time.

1. Strongly agree
2. Somewhat agree
3. 50-50
4. Somewhat disagree
5. Strongly disagree

62) If yes, what activities / services?
(Enumerator: check all that are mentioned; do not read the list)

1. Maternal health services
2. Vaccinations
3. Providing health information
4. *… (Other typical CHV activities to include in this list?)*

5. Other: ___________________________

63) Please indicate to what extent you agree with the statement: There are other responsibilities outside of my work as a CHV that I would normally have performed in the last month that I did not because my work as a CHV took too much of my time.

1. Strongly agree
2. Somewhat agree
3. 50-50
4. Somewhat disagree
5. Strongly disagree

64) *If "strongly agree" or "somewhat agree" above:* What responsibilities outside of your role as a CHV have been affected? (childcare, other volunteering, church, education/study, family, etc.)
(Enumerator: check all that are mentioned; do not read the list)

1. Childcare
2. Family responsibilities
3. Other work
4. Other volunteering / community activities
5. Religious activities

6. Other: ___________________________

65) What, if an, relationships have been affected by your work as a CHV on this malaria project? *(Enumerator: check all that are mentioned; do not read the list)*

1. Children
2. Spouse
3. Siblings
4. Parents
5. Other family members not mentioned above

For peer review only

6. Neighbors
7. Friends
8. Other: ____________________________

66) What do you think are the most important services you provide as a CHV?
(Enumerator: check all that are mentioned; do not read the list)

1. Malaria testing
2. Provide vouchers for malaria drugs
3. Mosquito net distribution
4. Provide other malaria-related services/counseling
5. Polio campaign participation
6. Other vaccination campaign(s)
7. Child health services not mentioned above
8. Maternal health services not mentioned above
9. Giving health education to households
10. None
11. Other: ____________________________
____
____

67) What do you think is the most important service you provide as a CHV?
(Enumerator: Only allow one answer)

1. Malaria testing
2. Provide vouchers for malaria drugs
3. Mosquito net distribution
4. Provide other malaria-related services/counseling
5. Polio campaign participation
6. Other vaccination campaign(s)
7. Child health services not mentioned above
8. Maternal health services not mentioned above
9. Giving health education to households
10. None
11. Other: ____________________________
____
____

68) In order to monitor the quality of the tests done, some CHVs in this project were randomly chosen to use a device called a DekiReader that automatically reads the RDT cassette and displays the results. Did you use this device?

1. Yes
2. No
3. I was selected but I couldn't use it (too difficult)
4. Not sure

If 1 or 3, ask next 3 questions:

69) To what extent would you agree with the statement, "The DekiReader was easy to use correctly"?

1. Strongly agree
2. Somewhat agree
3. 50-50
4. Somewhat disagree
5. Strongly disagree

70) To what extent would you agree with the statement, "The Deki Reader helped me improve my ability to perform RDTs correctly"?
    1. Strongly agree
    2. Somewhat agree
    3. 50-50
    4. Somewhat disagree
    5. Strongly disagree

71) If you had the option to use the Deki Reader for every test as part of your routine work, would you want to have it or not?
    1. Yes
    2. No
    3. Don't Know / Unsure

72) Do you have any concerns or suggestions to improve the implementation of the malaria project?
    (Open-ended response): ______________________________

    ____

    ____

    ____

Thank you for your participation and feedback!

For peer review only

Checklist for performing Rapid Diagnostic Testing correctly

Name of CHV ____________________ Community Unit ___________________

Name of observer____________________________ Date _____________________

No.	Task	Task Category	Correctly done<br>No = 0<br>Yes =1	Observer's comments
**A.**	**RDT preparation**			
1.	Assembles RDT packet, buffer, swab, lancet			
2.	Read expiration date			
3.	Remove contents of test packet			
4.	Write patient's name on cassette			
**B.**	**Patient preparation**			
5.	Identify patient's details and record patient's name and date on the RDT cassette			
6.	Explain procedure to patient. Provide reassurance, as needed			
7.	Wear gloves	Safety		
**C.**	**Blood collection + dispensing**			
8.	Select 4th finger of left hand for blood collection			
9.	Clean finger with alcohol swab and allow it to dry	Safety		
10.	Prick finger firmly with sterile lancet	Safety		
11.	Discard lancet in sharps bin immediately after pricking finger. Do not set it down first	Safety		
12.	DO NOT squeeze finger excessively			
13.	Collect an adequate volume of blood with pipette	Accuracy		
14.	Dispense blood in correct well	Accuracy		
15.	Discards the pipette in the sharps box	Safety		
16.	Dispose of gloves, wrappers, alcohol swab, loop, desiccant and cassette in non-sharps container	Safety		
**D.**	**RDT procedure and reading results**			
17.	Dispense correct volume of buffer	Accuracy		
18.	Wait for twenty (20) minutes	Accuracy		
19.	Verify internal test control			
20.	Read results correctly	Accuracy		
**E.**	**Recording results**			
21.	Records date and time correctly on encounter form	Treatment		
**F.**	**Deliver the results**			
22.	Explain to the patient the results and what they mean	Treatment		

For peer review only

STROBE Statement—Checklist of items that should be included in reports of ***cross-sectional studies***

	Item No	Recommendation	Page No
**Title and abstract**	1	(*a*) Indicate the study's design with a commonly used term in the title or the abstract	1
		(*b*) Provide in the abstract an informative and balanced summary of what was done and what was found	2-3
**Introduction**			
Background/rationale	2	Explain the scientific background and rationale for the investigation being reported	5-7
Objectives	3	State specific objectives, including any prespecified hypotheses	6
**Methods**			
Study design	4	Present key elements of study design early in the paper	7
Setting	5	Describe the setting, locations, and relevant dates, including periods of recruitment, exposure, follow-up, and data collection	7-8
Participants	6	(*a*) Give the eligibility criteria, and the sources and methods of selection of participants	9-10
Variables	7	Clearly define all outcomes, exposures, predictors, potential confounders, and effect modifiers. Give diagnostic criteria, if applicable	11
Data sources/ measurement	8*	For each variable of interest, give sources of data and details of methods of assessment (measurement). Describe comparability of assessment methods if there is more than one group	*12-13*
Bias	9	Describe any efforts to address potential sources of bias	
Study size	10	Explain how the study size was arrived at	9
Quantitative variables	11	Explain how quantitative variables were handled in the analyses. If applicable, describe which groupings were chosen and why	7-8
Statistical methods	12	(*a*) Describe all statistical methods, including those used to control for confounding	12-13
		(*b*) Describe any methods used to examine subgroups and interactions	
		(*c*) Explain how missing data were addressed	
		(*d*) If applicable, describe analytical methods taking account of sampling strategy	12-13
		(*e*) Describe any sensitivity analyses	
**Results**			
Participants	13*	(a) Report numbers of individuals at each stage of study—eg numbers potentially eligible, examined for eligibility, confirmed eligible, included in the study, completing follow-up, and analysed	15
		(b) Give reasons for non-participation at each stage	
		(c) Consider use of a flow diagram	
Descriptive data	14*	(a) Give characteristics of study participants (eg demographic, clinical, social) and information on exposures and potential confounders	15
		(b) Indicate number of participants with missing data for each variable of interest	
Outcome data	15*	Report numbers of outcome events or summary measures	16, Table 1

For peer review only

Main results	16	(*a*) Give unadjusted estimates and, if applicable, confounder-adjusted estimates and their precision (eg, 95% confidence interval). Make clear which confounders were adjusted for and why they were included	16-24 Table 2-5
		(*b*) Report category boundaries when continuous variables were categorized	Tables
		(*c*) If relevant, consider translating estimates of relative risk into absolute risk for a meaningful time period	
Other analyses	17	Report other analyses done—eg analyses of subgroups and interactions, and sensitivity analyses	
**Discussion**			
Key results	18	Summarise key results with reference to study objectives	24
Limitations	19	Discuss limitations of the study, taking into account sources of potential bias or imprecision. Discuss both direction and magnitude of any potential bias	26-27
Interpretation	20	Give a cautious overall interpretation of results considering objectives, limitations, multiplicity of analyses, results from similar studies, and other relevant evidence	27
Generalisability	21	Discuss the generalisability (external validity) of the study results	27
**Other information**			
Funding	22	Give the source of funding and the role of the funders for the present study and, if applicable, for the original study on which the present article is based	28

*Give information separately for exposed and unexposed groups.

Note: An Explanation and Elaboration article discusses each checklist item and gives methodological background and published examples of transparent reporting. The STROBE checklist is best used in conjunction with this article (freely available on the Web sites of PLoS Medicine at http://www.plosmedicine.org/, Annals of Internal Medicine at http://www.annals.org/, and Epidemiology at http://www.epidem.com/). Information on the STROBE Initiative is available at www.strobe-statement.org.

For peer review only
